# Population Genomics: Whole-Genome Analysis of Polymorphism and Divergence in Drosophila simulans

**DOI:** 10.1371/journal.pbio.0050310

**Published:** 2007-11-06

**Authors:** David J Begun, Alisha K Holloway, Kristian Stevens, LaDeana W Hillier, Yu-Ping Poh, Matthew W Hahn, Phillip M Nista, Corbin D Jones, Andrew D Kern, Colin N Dewey, Lior Pachter, Eugene Myers, Charles H Langley

**Affiliations:** 1 Department of Evolution and Ecology, University of California Davis, Davis, California, United States of America; 2 Center for Population Biology, University of California Davis, Davis, California, United States of America; 3 Genome Sequencing Center, Washington University School of Medicine, St. Louis, Missouri, United States of America; 4 Institute of Molecular and Cellular Biology, National Tsing Hua University, Hsinchu, Taiwan Authority; 5 Research Center for Biodiversity, Academica Sinica, Taipei, Taiwan Authority; 6 Department of Biology, Indiana University, Bloomington, Indiana, United States of America; 7 School of Informatics, Indiana University, Bloomington, Indiana, United States of America; 8 Department of Biology, University of North Carolina, Chapel Hill, North Carolina, United States of America; 9 Carolina Center for Genome Sciences, University of North Carolina, Chapel Hill, North Carolina, United States of America; 10 Center for Biomolecular Science and Engineering, University of California Santa Cruz, Santa Cruz, California, United States of America; 11 Department of Biostatistics and Medical Informatics, University of Wisconsin, Madison, Wisconsin, United States of America; 12 Department of Mathematics, University of California, Berkeley, California, United States of America; 13 Department of Computer Science, University of California, Berkeley, California, United States of America; Duke University, United States of America

## Abstract

The population genetic perspective is that the processes shaping genomic variation can be revealed only through simultaneous investigation of sequence polymorphism and divergence within and between closely related species. Here we present a population genetic analysis of Drosophila simulans based on whole-genome shotgun sequencing of multiple inbred lines and comparison of the resulting data to genome assemblies of the closely related species, D. melanogaster and D. yakuba. We discovered previously unknown, large-scale fluctuations of polymorphism and divergence along chromosome arms, and significantly less polymorphism and faster divergence on the *X* chromosome. We generated a comprehensive list of functional elements in the D. simulans genome influenced by adaptive evolution. Finally, we characterized genomic patterns of base composition for coding and noncoding sequence. These results suggest several new hypotheses regarding the genetic and biological mechanisms controlling polymorphism and divergence across the *Drosophila* genome, and provide a rich resource for the investigation of adaptive evolution and functional variation in D. simulans.

## Author Summary

Population genomics, the study of genome-wide patterns of sequence variation within and between closely related species, can provide a comprehensive view of the relative importance of mutation, recombination, natural selection, and genetic drift in evolution. It can also provide fundamental insights into the biological attributes of organisms that are specifically shaped by adaptive evolution. One approach for generating population genomic datasets is to align DNA sequences from whole-genome shotgun projects to a standard reference sequence. We used this approach to carry out whole-genome analysis of polymorphism and divergence in Drosophila simulans, a close relative of the model system, D. melanogaster. We find that polymorphism and divergence fluctuate on a large scale across the genome and that these fluctuations are probably explained by natural selection rather than by variation in mutation rates. Our analysis suggests that adaptive protein evolution is common and is often related to biological processes that may be associated with gene expression, chromosome biology, and reproduction. The approaches presented here will have broad applicability to future analysis of population genomic variation in other systems, including humans.

## Abstract 3

Low-coverage genome sequences from multiple *Drosophila simulans* strains provide the first comprehensive view of polymorphism and divergence in the fruit fly.

## Introduction

Given the long history of *Drosophila* as a central model system in evolutionary genetics beginning with the origins of empirical population genetics in the 1930s, it is unsurprising that *Drosophila* data have inspired the development of methods to test population genetic theories using DNA variation within and between closely related species [1–4]. These methods rest on the supposition of the neutral theory of molecular evolution that polymorphism and divergence are manifestations of mutation and genetic drift of neutral variants at different time scales [5]. Under neutrality, polymorphism is a “snapshot” of variation, some of which ultimately contributes to species divergence as a result of fixation by genetic drift. Natural selection, however, may cause functionally important variants to rapidly increase or decrease in frequency, resulting in patterns of polymorphism and divergence that deviate from neutral expectations [1,2,6]. A powerful aspect of inferring evolutionary mechanism in this population genetic context is that selection on sequence variants with miniscule fitness effects, which would be difficult or impossible to study in nature or in the laboratory but are evolutionarily important, may cause detectable deviations from neutral predictions. Another notable aspect of these population genetic approaches is that they facilitate inferences about recent selection—which may be manifest as reduced polymorphism or elevated linkage disequilibrium—or about selection that has occurred in the distant past—which may be manifest as unexpectedly high levels of divergence. The application of these conceptual advances to the study of variation in closely related species has resulted in several fundamental advances in our understanding of the relative contributions of mutation, genetic drift, recombination, and natural selection to sequence variation. However, it is also clear that our genomic understanding of population genetics has been hobbled by fragmentary and nonrandom population genetic sampling of genomes. Thus, the full value of genome annotation has not yet been applied to the study of population genetic mechanisms.

Combining whole-genome studies of genetic variation within and between closely related species (i.e., population genomics) with high-quality genome annotation offers several major advantages. For example, we have known for more than a decade that regions of the genome experiencing reduced crossing over in *Drosophila* tend to show reduced levels of polymorphism yet normal levels of divergence between species [7–10]. This pattern can only result from natural selection reducing levels of polymorphism at linked neutral sites, because it violates the neutral theory prediction of a strong positive correlation between polymorphism and divergence [5]. However, we have no general genomic description of the physical scale of variation in polymorphism and divergence in *Drosophila* and how such variation might be related to variation in mutation rates, recombination rates, gene density, natural selection, or other factors. Similarly, although several *Drosophila* genes have been targets of molecular population genetic analysis, in many cases, these genes were not randomly chosen but were targeted because of their putative association with phenotypes thought to have a history of adaptive evolution [11,12]. Such biased data make it difficult to estimate the proportion of proteins diverging under adaptive evolution. In a similar vein, the unique power of molecular population genetic analysis, when used in concert with genome annotation, could fundamentally alter our notions about phenotypic divergence due to natural selection. This is because our current understanding of phenotypic divergence and its causes is based on a small and necessarily highly biased description of phenotypic variation. Alternatively, a comprehensive genomic investigation of adaptive divergence could use genome annotations to reveal large numbers of new biological processes previously unsuspected of having diverged under selection. Here we present a population genomic analysis of D. simulans. D. simulans and D. melanogaster are closely related and split from the outgroup species, D. yakuba, several million years ago [13–15]. The vast majority of D. simulans and D. yakuba euchromatic DNA is readily aligned to D. melanogaster, which permits direct use of D. melanogaster annotation for investigation of polymorphism and divergence and allows reliable inference of D. simulans–D. melanogaster ancestral states over much of the genome. Our analysis uses a draft version of a D. yakuba genome assembly (aligned to the D. melanogaster reference sequence) and a set of light-coverage, whole-genome shotgun data from multiple inbred lines of D. simulans, which were syntenically aligned to the D. melanogaster reference sequence.

## Results/Discussion

### Genomes and Assemblies

Seven lines of D. simulans and one line of D. yakuba were sequenced at the Washington University Genome Sequencing Center (the white paper can be found at http://www.genome.gov/11008080). The D. simulans lines were selected to capture variation in populations from putatively ancestral geographic regions [16], recent cosmopolitan populations, and strains encompassing the three highly diverged mitochondrial haplotypes previously described for the species [17]. These strains have been deposited at the Tucson *Drosophila* Stock Center (http://stockcenter.arl.arizona.edu). A total of 2,424,141 D. simulans traces and 2,245,197 D. yakuba traces from this project have been deposited in the National Center for Biotechnology Information (NCBI) trace archive. D. simulans syntenic assemblies were created by aligning trimmed, uniquely mapped sequence traces from each D. simulans strain to the euchromatic D. melanogaster reference sequence (v4). Two strains from the same population, *sim4* and *sim6*, were unintentionally mixed prior to library construction; reads from these strains were combined to generate a single, deeper, syntenic assembly (see Materials and Methods), which is referred to as *SIM4/6*. The other strains investigated are referred to as *C167.4*, *MD106TS*, *MD199S*, *NC48S*, and *w^501^*. Thus, six (rather than seven) D. simulans syntenic assemblies are the objects of analysis. Details on the fly strains and procedures used to create these assemblies, including the use of sequence quality scores, can be found in Materials and Methods. The coverages (in Mbp) for *C167.4*, *MD106TS*, *MD199S*, *NC48S*, *SIM4/6*, and *w^501^*, are 56.9, 56.3, 63.4, 42.6, 89.8, and 84.8, respectively. A D. yakuba strain *Tai18E2* whole-genome shotgun assembly (v2.0; http://genome.wustl.edu/) generated by the Parallel Contig Assembly Program (PCAP) [18] was aligned to the D. melanogaster reference sequence (Materials and Methods). The main use of the D. yakuba assembly was to infer states of the *D. simulans–D. melanogaster* ancestor. For many analyses, we used divergence estimates for the D. simulans lineage or the D. melanogaster lineage (from the inferred *D. simulans–D. melanogaster* ancestor) rather than the pairwise (i.e., unpolarized) divergence between these species. These lineage-specific estimates are often referred to as “D. simulans divergence,” “D. melanogaster divergence,” or “polarized divergence.”

A total of 393,951,345 D. simulans base pairs and 102,574,197 D. yakuba base pairs were syntenically aligned to the D. melanogaster reference sequence. Several tens of kilobases of repeat-rich sequences near the telomeres and centromeres of each chromosome arm were excluded from our analyses (Materials and Methods). D. simulans genes were conservatively filtered for analysis based on conserved physical organization and reading frame with respect to the D. melanogaster reference sequence gene models (Materials and Methods). We took this conservative approach so as to retain only the highest quality D. simulans data for most inferences. The number of D. simulans genes remaining after filtering was 11,466. Ninety-eight percent of coding sequence (CDS) nucleotides from this gene set are covered by at least one D. simulans allele. The average number of lines sequenced per aligned D. simulans base was 3.90. For several analyses in which heterozygosity and divergence per site were estimated, we further filtered the data so as to retain only genes or functional elements (e.g., untranslated regions [UTRs]) for which the total number of bases sequenced across all lines exceeded an arbitrary threshold (see Materials and Methods). The numbers of genes for which we estimated coding region expected heterozygosity, unpolarized divergence, and polarized divergence were 11,403, 11,439, and 10,150, respectively. Coverage on the *X* chromosome was slightly lower than autosomal coverage, which is consistent with less *X* chromosome DNA than autosomal DNA in mixed-sex DNA preps. Variable coverage required analysis of individual coverage classes (*n* = 1–6) for a given region or feature, followed by estimation and inference weighted by coverage (Materials and Methods). The D. simulans syntenic alignments are available at http://www.dpgp.org/. An alternative D. simulans “mosaic” assembly, which is available at http://www.genome.wustl.edu/, was created independently of the D. melanogaster reference sequence.

### General Patterns of Polymorphism and Divergence

#### Nucleotide variation.

**Figure 1:**
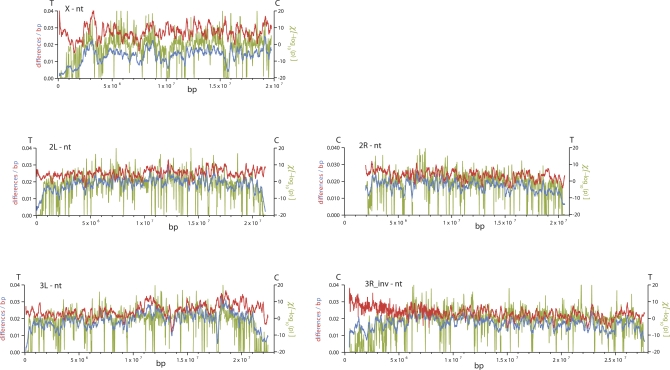
Patterns of Polymorphism and Divergence of Nucleotides along Chromosome Arms Nucleotide π (blue) and *div* on the D. simulans lineage (red) in 150-kbp windows are plotted every 10 kbp. χ[–log(*p*)] (olive) as a measure of deviation (+ or –) in the proportion of polymorphic sites in 30-kbp windows is plotted every 10 kbp (see Materials and Methods). C and T correspond to locations of centromeres and telomeres, respectively. Chromosome arm *3R* coordinates correspond to D. simulans locations after accounting for fixed inversion on the D. melanogaster lineage.

We observed 2,965,987 polymorphic nucleotides, of which 43,878 altered the amino acid sequence; 77% of sampled D. simulans genes were segregating at least one amino acid polymorphism. The average, expected nucleotide heterozygosity (hereafter, “heterozygosity” or “π_nt_”) for the *X* chromosome and autosomes was 0.0135 and 0.0180, respectively. *X* chromosome π_nt_ was not significantly different from that of the autosomes (after multiplying *X* chromosome π_nt_ by 4/3, to correct for *X*/autosome effective population size differences when there are equal numbers of males and females; see [19]). However, *X* chromosome divergence was greater than autosomal divergence in all three lineages (50-kb windows; Table 1, Table S1, Figure 1, Dataset S8). We will discuss this pattern in greater detail below.

**Table 1:**
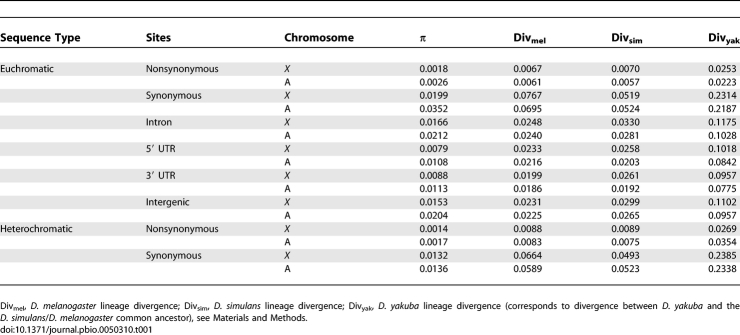
Autosome and *X* Chromosome Weighted Averages of Nucleotide Heterozygosity (π) and Lineage Divergence

Not surprisingly, many patterns of molecular evolution identified from previously published datasets were confirmed in this genomic analysis. For example, synonymous sites and nonsynonymous sites were the fastest and slowest evolving sites types, respectively [20–24]. Nonsynonymous divergence (*dN*) and synonymous divergence (*dS*) were positively, though weakly, correlated (*r* ^2^ = 0.052, *p* < 0.0001) [25–27], and *dN* was weakly, negatively correlated with CDS length (Spearman's ρ = −0.03, *p* = 0.0005) [28,29]. More generally, longer functional elements showed smaller D. simulans divergence than did shorter elements (intron Spearman's ρ = −0.33; intergenic Spearman's ρ = −0.39; 3′ UTRs Spearman's ρ = −0.11: all show *p* < 0.0001) [21,30].

#### Insertion/deletion (indel) variation.

**Figure 2:**
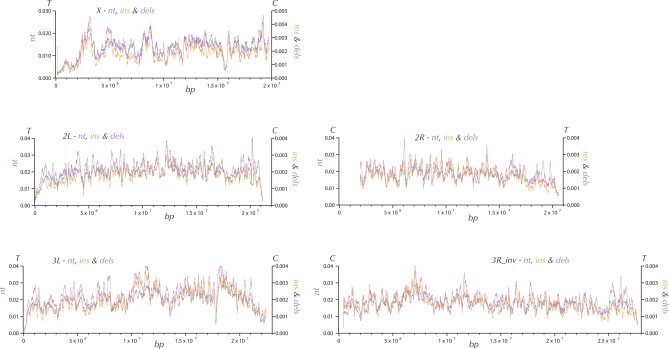
Patterns of Polymorphism for Nucleotides, Small Insertions, and Small Deletions along Chromosome Arms π for nucleotides (blue), π for small (≤ 10 bp) insertions (orange), and π for small (≤ 10 bp) deletions (orchid) among the D. simulans lines in 150-kbp windows are plotted every 10 kbp (see Materials and Methods). C and T correspond to locations of centromeres and telomeres, respectively. Chromosome arm *3R* coordinates correspond to D. simulans locations after accounting for fixed inversion on the D. melanogaster lineage.

We investigated only small indels (≤10 bp), because they were inferred with high confidence (Materials and Methods). Variants were classified with respect to the D. melanogaster reference sequence; divergence estimates were unpolarized. An analysis of transposable element variation can be found in Text S1. Estimates of small-indel heterozygosity for the *X* chromosome and autosomes (Table S1) were lower than estimates of nucleotide heterozygosity [31]. Interestingly, variation in nucleotide and indel heterozygosity across chromosome arms was highly correlated ([32], Figures 1 and 2; Spearman's ρ between 0.45 and 0.69, *p* < 10^−4^ for each arm). Deletion heterozygosity and divergence were consistently greater than insertion heterozygosity and divergence (Figures S1 and S2, Datasets S11–S15) for both the *X* chromosome and the autosomes, which supports and extends previous claims, based on analysis of repetitive sequences [33], of a general mutational bias for deletions in *Drosophila*.

#### D. simulans autosomal π_nt_ and divergence are of similar magnitude.

Mean polarized autosomal divergence (50-kb windows; 0.024) was only slightly greater than mean autosomal π_nt_ (0.018), even with regions of severely reduced π_nt_ near telomeres and centromeres included. Indeed, estimates of π_nt_ for several genomic regions are roughly equal to the genomic average polarized divergence (Figure 1), suggesting the existence of large numbers of shared polymorphisms in D. simulans and D. melanogaster; such variants should be overrepresented in regions of higher nucleotide heterozygosity in D. simulans. These patterns suggest that the average time to the most recent common ancestor of D. simulans alleles is nearly as great as the average time of the most recent common ancestor of D. simulans and D. melanogaster. The similarity in scale of polymorphism and divergence in D. simulans also suggests that many of the neutral mutations that have fixed in D. simulans were polymorphic in the common ancestor of the two species. As we discuss below, this has implications for interpreting chromosomal patterns of polymorphism and divergence in this species.

As expected under the neutral model, and given the observation that much of the D. simulans lineage divergence is attributable to polymorphism, D. simulans π_nt_ and divergence (50-kb windows) were highly, significantly correlated (autosome Spearman's ρ = 0.56, *p* < 0.0001: *X* chromosome Spearman's ρ = 0.48, *p* < 0.0001) [5]. Moreover, the genetic and population genetic processes shaping patterns of divergence along chromosome arms appear to operate in a similar manner in D. simulans and D. melanogaster, as polarized divergence (50-kb windows) for the two lineages was highly correlated (Spearman's ρ = 0.74; *p* < 0.0001). Nevertheless, some regions of the genome showed highly significant increases in divergence in either the D. simulans or the D. melanogaster lineage (see below).

#### Variation near centromeres and telomeres.

Figure 1 and Figure S1 support previous reports documenting severely reduced levels of polymorphism in the most proximal and distal euchromatic regions of *Drosophila* chromosome arms [7,10,34–36]. The fact that divergence in such regions (Materials and Methods) is only slightly lower (50-kb median = 0.0238) than that of the rest of the euchromatic genome (50-kb median = 0.0248) (Mann-Whitney *U*, *p* < 0.0001), supports the hypothesis that reduced π_nt_ in these regions is due to selection at linked sites rather than reduced neutral mutation rates [1,3,6]. Genes that are located in repetitive regions of chromosomes near telomeres and centromeres (Materials and Methods), which we refer to as “heterochromatic,” showed moderately reduced nonsynonymous and synonymous heterozygosity compared with other genes (Table 1, Dataset S6) [37] and showed a substantially higher ratio of nonsynonymous-to-synonymous polymorphism and divergence relative to other genes (Table S2) [38].

Interestingly, the magnitude and physical extent of reduced π_nt_ near telomeres and centromeres appears to vary among arms. Moreover, the physical scale over which divergence varied along the basal region of *3R* appears to be much smaller than the scale for other arms, which is seen in Figure 1 as a more compressed, thick red line representing divergence. These heterogeneous patterns of sequence variation near centromeres and telomeres across chromosome arms may reflect real differences. For example, genetic data from D. melanogaster suggest that the centromere-associated effects of reduced crossing-over are greater for the autosomes than for the *X* chromosome and also suggest that the *X* chromosome telomere is associated with a stronger reduction in crossing-over compared with the autosomal telomeres [39]. Alternatively, some of the heterogeneity between chromosome arms in the centromere proximal regions may reflect variation in the amount of repeat-rich sequence excluded from the analysis (Materials and Methods).

### *X* versus Autosome Divergence

#### Faster-*X* divergence.

The *X* chromosome differs from the autosomes in its genetics as well as in its population genetics [40,41]. These differences have motivated several attempts to compare patterns of polymorphism and divergence on these two classes of chromosomes and to use such comparisons to test theoretical population genetic models [19,41]. For example, several population genetic models (e.g., recessivity of beneficial mutations) predict faster evolution of *X*-linked versus autosomal genes [42]. Nevertheless, there is currently no statistical support for greater divergence of *X*-linked versus autosomal genes in *Drosophila* [19,43,44].

The genomic data presented here clearly show that the *X* is evolving faster than the autosomes. For example, median (standard error [SE]) *X* versus autosome divergence for 50-kb windows was 0.0274 (0.0003) versus 0.0242 (0.0001) for D. simulans, 0.0233 (0.0002) versus 0.0223 (0.0007) for D. melanogaster, and 0.1012 (0.0007) versus 0.0883 (0.0003) for D. yakuba. The *X* evolves significantly faster than the autosomes in D. simulans, D. melanogaster, and D. yakuba (Tables 1 and S1; 50-kb windows, Mann-Whitney *U*; *z* = 4.99, 12.92, and 14.68 for D. melanogaster, D. simulans, and D. yakuba respectively, all *p* < 0.0001), although the faster-*X* effect appeared to be considerably smaller in D. melanogaster than in D. simulans or D. yakuba. Moreover, of the 18 lineage divergence estimates (six site types and three lineages), only one, D. simulans synonymous sites, failed to show faster-*X* evolution (Table 1). However, not all classes of site/lineages showed statistically significant faster-*X* evolution (Table S3). Thus, the faster-*X* effect is likely to be general for *Drosophila* but vary in magnitude across lineages and site types. Mean *X* chromosome divergence in previous analyses of smaller datasets [19,43,44] was higher (though not significantly so) than autosome divergence, in agreement with these genomic results. Finally, indel divergence also showed a faster-*X* effect (Mann-Whitney *U*, *p* < 0.0001 for both insertions and deletions).

Interestingly, the lengths of coding regions, introns, intergenic regions, and 5′ and 3′ UTRs were significantly longer (Mann-Whitney *U*, all five have *p* < 0.0001) for the *X* chromosome than for the autosomes in D. melanogaster [45]. Longer introns, intergenic sequences, and genes tend to evolve more slowly than shorter functional elements (above and [45]), suggesting that the faster-*X* inference is conservative. Perhaps the *X* chromosome requires additional sequences for proper regulation through dosage compensation (e.g., [46–48]) or proper large-scale organization in the nucleus [49]. Alternatively, if directional selection were more common on the *X* chromosome, then Hill-Robertson effects [50] could favor insertions, because selection is expected to be more effective when there is more recombination between selected sites. However, the fact that *X*-linked deletion divergence is much greater than insertion divergence, at least for small indels (see below), does not support this idea. Further analysis of larger indels could clarify this matter. Finally, under the premise that ancestral polymorphism makes a considerable contribution to D. simulans divergence, lower *X* chromosome polymorphism (relative to ancestral autosome polymorphism) would also make the faster-*X* inference conservative.

As noted above, faster-*X* evolution has several possible explanations, including recessivity of beneficial mutations, underdominance, more frequent directional selection on males than on females, higher mutation rates in females than in males, or higher mutation rates on the *X* chromosome versus the autosomes [19,40–42]. The fact that faster-*X* evolution is observed across most site types is consistent with the hypothesis that *X* chromosome mutation rates are greater than autosomal mutation rates. The *X* chromosome is distinct from the autosomes in that it is dosage compensated in males through hypertranscription of *X*-linked genes [51–53]. Dosage compensation of the *Drosophila* male germline [52] could result in higher *X*-linked mutation rates if chromatin conformation associated with hypertranscription increases mutation rates. Indeed, cytological and biochemical studies of the male *Drosophila* polytene chromosomes suggest that the *X* has a fundamentally different chromatin organization than the autosomes [54]. Alternatively, DNA repair in the heterogametic male could have different properties than repair in females. In addition to the possible contribution of elevated *X*-linked mutation rates to faster-*X* evolution, some aspects of the data support a role for selection in elevating *X* chromosome substitution rates. For example, the three site classes that showed the greatest *X*/autosome divergence ratio in D. simulans (nonsynonymous, 5′ UTR and 3′ UTR) also showed the strongest evidence for adaptive divergence in contrasts of polymorphic and fixed variants in D. simulans (see below). Furthermore, the observation of a significantly higher frequency of derived polymorphic variants on the *X* relative to the autosomes [55] (Table S4) is consistent with more adaptive evolution on the *X* chromosome [56,57]. However, there is no obvious enrichment of genes showing a history of recurrent adaptive protein evolution on the *X* chromosome (see below).

In addition to the overall faster rate of *X* chromosome evolution, relative rate tests (Materials and Methods) revealed that the deviations of observed numbers of substitutions from neutral expectations are significantly greater for the *X* chromosome than for autosomes in both D. simulans and D. melanogaster (Mann-Whitney *U*, *p* = 1.3 × 10^−13^ and 1.4 × 10^−4^ for D. simulans and D. melanogaster, respectively). The magnitude of the deviations of D. simulans substitutions from expected numbers (Materials and Methods) varied along chromosome arms (Table S5 and Figure S3), with the *X* chromosome showing a particularly strong physical clustering of unusual regions. Though these patterns could be explained by natural selection [56,58], the possible role of demography or differences in the distribution of ancestral polymorphism within and among chromosome arms as factors contributing to these patterns require further study.

#### Greater *X*-linked deletion divergence.

Although nucleotide and indel polymorphism and divergence showed similar patterns across the genome, there was a great disparity between *X* chromosome and autosome deletion divergence in D. simulans (Figure S1). Whereas *X* chromosome nucleotide divergence was only 14% higher than autosomal nucleotide divergence, *X* chromosome deletion divergence (10-kb window median = 0.0056) was about 60% higher than autosomal deletion divergence (10-kb window median = 0.0035). Furthermore, *X* chromosome deletion divergence was much larger than *X* chromosome insertion divergence (10-kb window median = 0.0035). The lack of a homologous *X* chromosome for recombinational repair in G1 of the cell cycle in males, or an *X* chromosome bias for gene conversion of small deletions over small insertions, could contribute to this pattern. However, any neutral equilibrium explanation for accelerated *X*-linked deletion divergence should predict that the *X* shows a disproportionately high ratio of deletion-to-insertion heterozygosity relative to the autosomes, which was not observed. More generally, the ratio of deletion-to-insertion divergence was greater than the ratio of deletion-to-insertion heterozygosity (Mann-Whitney *U*, *p* < 0.0001), with the *X* showing a larger discrepancy than the autosomes (Mann-Whitney *U*, *p* < 0.0001). This can be explained either by invoking a change in the mutation process (e.g., a recent mutational bias shift towards insertions) or by natural selection (e.g., deletions more often favored relative to insertions).

### Chromosomal Gradients of Divergence

One of the main goals of large-scale investigations of sequence divergence is to characterize the many biological factors influencing variation in substitution rates throughout the genome. Most analyses of *Drosophila* data focus on variation in functional constraints or directional selection as the main cause of heterogeneity in substitution rates across genes or functional elements [20,21]. However, the available data have been too sparse to detect any patterns of increasing or decreasing divergence along chromosome arms.

Centromere proximal regions tend to be more divergent than distal regions (Figure 1, Figure S4, and Table S5). This pattern is more consistent for D. simulans than for D. melanogaster. Proximal euchromatic regions tend to have lower inferred ancestral GC content compared with distal regions of chromosome arms (Figure S4 and Table S5), which is consistent with the observation that D. simulans divergence was negatively correlated with inferred ancestral GC content (Materials and Methods) (50-kb windows, Spearman's ρ = −0.23, *p* = 1.4 × 10^−26^) [30]. The correlation between ancestral GC content and divergence was much weaker and only marginally significant for D. melanogaster (Spearman's ρ = −0.05, *p* = 0.03). However, while chromosomal gradients of divergence were observed for most chromosome arms (Figure S4 and Table S5), inferred ancestral GC content tends to show a less-consistent pattern. For example, some arms showed a more U-shaped distribution, with euchromatic regions near centromeres and telomeres tending to have higher estimated ancestral GC content (Figure S5). More proximal and distal regions also tend to have reduced crossing-over [39], which is consistent with the observation that inferred ancestral GC content is negatively correlated with cM/kb (Materials and Methods) on the *X* chromosome (Spearman's ρ = −0.33, *p* = 0.0002) [59], the only chromosome arm for which we investigated correlates of recombination rate variation (see below).

The neutral model of evolution predicts that gradients of divergence along chromosome arms are explained by gradients of functional constraint or mutation rates. For example, higher divergence in regions near centromeres could be explained if such regions harbor a lower density of functional elements (e.g., genes). However, with the exception of chromosome arm *2L* (Spearman's ρ = −0.19, *p* = 6 × 10^−5^), variation in coding sequence density (CDS bases per 50-kb window) showed no significant chromosomal proximal–distal trend, suggesting that variation in constraint that is associated with coding density plays, at best, a small part in explaining chromosomal gradients of divergence. More generally, the expectation of a negative correlation between coding density and nucleotide divergence in D. simulans was not met. This seemingly counterintuitive result probably reflects the fact that exons constitute a relatively small fraction of the genome and were not dramatically less diverged (0.016) compared with intergenic DNA (0.027).

If proximal–distal gradients of decreasing divergence along chromosome arms result from variation in mutation rates, then the neutral theory predicts that we should observe similar gradients of polymorphism. This is the case for some chromosome arms but not others (Figure 1 and Table S5), after regions of reduced π_nt_ in the most distal/proximal regions are excluded (Materials and Methods; this result is robust to variation in the extent of proximal and distal chromosomal regions removed from the analysis). Thus, variable neutral mutation rates alone is an insufficient explanation for the overall genomic patterns of variation. Below we address the possibility that recombination rate variation contributes to variation in D. simulans π_nt_ and divergence across chromosome arms.

### Fluctuations in Polymorphism and Divergence along Chromosome Arms

There was considerable variance of polymorphism and divergence across chromosome arms, even when regions of severely reduced heterozygosity near centromeres and telomeres were excluded. Figure 1 clearly shows that variance in polymorphism and divergence is not randomly arranged, but rather appears to be spatially structured on the scale of several tens of kilobases. These qualitative visual assessments were supported by significant statistical autocorrelations (Materials and Methods) for nucleotide heterozygosity and divergence across all chromosome arms (Table S6) [60]. Furthermore, the strength of this autocorrelation appeared to differ across arms, because *X* and *3L* show evidence of stronger correlations over longer distances (Figure 1). The strength of autocorrelation is consistently higher for heterozygosity than for divergence.

Under the neutral theory, fluctuations in polymorphism and divergence could be the result of variation in gene density, with windows that have more exons per kb showing lower polymorphism and divergence. This expectation was not met. Indeed, for 50-kb autosome windows (but not *X*-linked windows), divergence is positively correlated with coding density (Spearman's ρ = 0.12, *p* < 0.0001). This is consistent with an important role of directional selection on coding sequence to genome divergence, a point we will revisit in several analyses below. In contrast to the positive correlation between coding density and divergence, we found a negative correlation between coding density and D. simulans π_nt_ (autosome Spearman's ρ = −0.10, *p* < 0.0001; *X* Spearman's ρ = 0.29, *p* < 0.0001). Overall, the contrasting correlations between coding density and polymorphism versus divergence suggest that directional selection in exon-rich regions generates greater divergence and reduced polymorphism due to hitchhiking effects [3,6,61].

#### Correlations between recombination rates and sequence variation.

**Figure 3:**
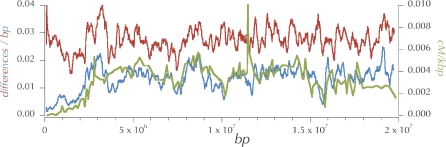
Rate of Crossing-Over per Base Pair (Green), Nucleotide Polymorphism (Blue) and Nucleotide Divergence (Red) along the *X* Chromosome Nucletotide π (blue) and *div* on the D. simulans lineage (red) in 150-kbp windows are plotted every 10 kbp. Estimated rate of crossing-over (green) is plotted for specific genomic segments (see Materials and Methods).

One of the most unusual genomic regions, at around 3 Mb on the *X* chromosome (Figure 1), showed a large peak of both polymorphism and divergence. A previous analysis suggesting that this region might have higher-than-average recombination rates in D. melanogaster [62] motivated a more detailed investigation of the possible relationship between crossing-over versus polymorphism and divergence. Most estimates of crossing-over per base pair in D. melanogaster have been generated using approaches that could obscure megabase-scale variation in crossing-over along chromosome arms [63,64]. Figure 3 shows the results of a sliding window analysis of D. simulans π_nt_, divergence, and cM/kb (see Materials and Methods) along the *D. melanogaster X* chromosome, which has the best genetic data of the five major chromosome arms. There is a surprisingly strong correlation between *D. melanogaster X* chromosome recombination rates and D. simulans π_nt_ (Spearman's ρ = 0.45, *p* = 8.5 × 10^−8^), especially given the fact that the genetic data are from D. melanogaster. There is a weaker, marginally significant correlation between recombination and D. simulans divergence (Spearman's ρ = 0.17, *p* = 0.03) and D. melanogaster divergence (Spearman's ρ = 0.19, *p* = 0.03).

Under neutrality, if neutral mutation rates were correlated with recombination rates, regions with higher recombination rates would tend to be more polymorphic and diverged, thereby explaining why recombination rates are positively correlated with polymorphism and divergence. This neutral explanation makes two predictions. First, regions of severely reduced heterozygosity near telomeres and centromeres should show severely reduced divergence. Second, the correlation between recombination and divergence should be greater than the correlation between recombination and polymorphism. The second prediction reflects the fact that selection at linked sites, the effects of which should be correlated with recombination rates, is expected to reduce the correlation between mutation rate and polymorphism but not affect the correlation between mutation rate and divergence. The first prediction was not met by our data (Figure 1), and the converse of the second prediction was observed. An alternative population genetic explanation is that the observed correlations are partly attributable to hitchhiking effects of beneficial mutations.

**Figure 4:**
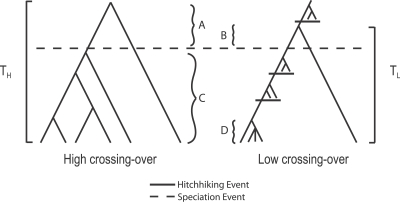
Hitchhiking Effects Can Induce a Correlation between Polymorphism and Divergence Hypothetical gene geneoligies in ancestral populations (A or B) and extant populations (C or D) for genomic regions of high crossing-over and low crossing-over (respectively) experiencing different hitchhiking effects. On average, time to the most recent common ancestor in the ancestral population is greater in regions of higher crossing-over (A) and therefore contributes more to the divergence, T_H_. Regions of lower crossing-over have smaller gene genealogies (D versus C) and less divergence (T_L_ versus T_H_).

Although there is no expected effect of recent hitchhiking on divergence at linked neutral sites [61], long-term, chronic hitchhiking effects can induce a correlation between recombination rates and both polymorphism and divergence (Figure 4), especially when the ancestral genealogy is a substantial part of divergence, as is the case in D. simulans (see above). Regions of higher recombination are expected to have experienced fewer hitchhiking effects, both in the recent and more ancient past. Such regions are expected to be associated with deeper genealogies in the ancestor and in extant samples, and thus should be more diverged and more polymorphic. The converse should be true for regions of lower recombination. This model posits that hitchhiking effects dominate chromosomal patterns of polymorphism in D. simulans and that much of the genome harbors levels of variation well below those expected in the absence of linked, directional selection [3,6]. Under this model, lower levels of nucleotide polymorphism in D. melanogaster than in D. simulans [24,65] could be due mainly to differences in the scale of hitchhiking effects in the two species. Furthermore, under this model, an as-yet-undetected proximal–distal gradient of recombination rate could contribute to proximal–distal gradients of polymorphism and divergence. Correlations between polymorphism and divergence may be weaker at telomere and centromere proximal regions (e.g., tip of the *X*, base of *3R*) compared to other regions due to larger-scale, recent hitchhiking effects on heterozygosity, which would tend to reduce any correlation between polymorphism and divergence induced by hitchhiking effects on ancestral variation. An alternative population genetic hypothesis for the high correlation between recombination and polymorphism is that the removal of deleterious variants by natural selection reduces variants at linked sites [1,66]**,** which is referred to as background selection. We will address this issue below.

Better meiotic exchange data for all of the chromosome arms in D. simulans and D. melanogaster will be necessary to investigate these ideas. If the *X* chromosome data are reliable, we predict that variation in the spatial distribution of crossing-over along chromosome arms is substantially different for the *X* versus autosomes of D. simulans and D. melanogaster [67]. Finally, we note that the region centered on location 3 Mb of the *D. simulans X* (Figure 1) is near a D. melanogaster meiotic “pairing site” [68] and harbors several copies of the *X* chromosome–enriched 1.688 satellite sequence [69]. It remains to be seen how the distribution of such entities across the genome contributes to patterns of polymorphism and divergence in *Drosophila*.

#### Correlated levels of nucleotide and indel polymorphism.

Although hitchhiking effects are expected to induce correlated patterns of variation along chromosome arms for SNPs versus indels, the extraordinarily high correlation observed (Figure 2) suggests the possibility that regional variation in mutation or repair could also contribute. Given that mutation rates differ for early versus late replicating DNA and that chromatin conformation affects both mutation and DNA repair, we investigated polymorphism and divergence in the context of genomic features related to replication [70]. Comparison of 10-kb windows (genomic data in Dataset S7) that overlap early-replicating regions on *2L* versus the remaining *2L* windows showed that early replicating origins had slightly higher heterozygosity (0.0188 versus 0.0179, *F* = 5.94 *p* = 0.015) and divergence (0.0266 versus 0.0251, *F* = 13.40, *p* = 0.0003). Origin-of-replication complexes appear to preferentially bind to AT-rich intron and intergenic sequences [70], consistent with the observation that the proximal regions of chromosomes tend to have lower GC content and greater divergence. Whole-genome data on origins of replication, preferably from germline cells, will be necessary to further investigate this issue. Nevertheless, the available data suggest that the effect of origins-of-replication on polymorphism and divergence is likely to be minor, and that the correlation between SNP and indel heterozygosity is likely caused by the effects of selection on linked sites.

It is also possible that spatial heterogeneity in transcription across the genome is associated with variation in mutation rates and thus, levels of polymorphism and divergence. Such an association could result from a correlation between transcription and replication [70,71] or because highly transcribed regions are associated with different mutation or repair than lowly transcribed regions. Though there are no data specifically from *Drosophila* germline cells, which are the only relevant cells for this question, to begin to address this issue we analyzed published gene expression data from D. melanogaster to identify a set of genes showing testis-biased expression (Materials and Methods). Median intron divergence in these genes (0.061) is much higher than the median intron divergence for the rest of the genome (0.042) (Mann Whitney *U*, *p* < 10^−4^), which is consistent with an association between mutation and germline transcription.

### Hitchhiking Effects in D. simulans

The analyses presented above, especially for the *X* chromosome data, strongly suggest that hitchhiking effects contribute to shaping patterns of polymorphism across the D. simulans genome. To provide a more quantitative assessment of the physical extent, magnitude, and biological basis of these hitchhiking effects, we carried out a genomic analysis of polymorphism and divergence in the context of the Hudson-Kreitman-Aguade (HKA) test [2] (Materials and Methods). The analysis should be thought of as a method for identifying unusual genomic regions rather than as a formal test of a specific model, since our data violate the assumptions of the simple neutral model (neutral alleles sampled from a single, equilibrium, panmictic population). The results (Figure 1, Datasets S6, S16–S20) statistically support our earlier contention and previous reports [7,8,10,34,36], that *Drosophila* chromosomes show greatly decreased polymorphism, relative to divergence, in both telomere- and centromere-proximal regions. The fact that corrected *X* chromosome heterozygosity was not significantly different from autosomal heterozygosity, although *X* chromosome divergence was significantly higher than autosomal divergence, supports a role for hitchhiking effects reducing nucleotide variation on the *X* chromosome.

Our previously mentioned result, that coding density is positively correlated with divergence and negatively correlated with polymorphism, suggested that hitchhiking effects of directional selection are more common in exonic sequence. The HKA-like analysis supports this contention. We identified regions of the genome that had either two or more consecutive, nonoverlapping 10-kb windows with *p* < 1 × 10^−6^ or four such windows with *p* < 0.01. The number of coding nucleotides per 10 kb in these “hitchhiking windows” (*n* = 378 windows, mean coding density = 2,980 bp) was much higher than coding density in other windows (*n* = 9,329, mean coding density = 1,860 bp) (Mann-Whitney *U*, *p* < 0.0001).

An alternative hypothesis for the strong correlation between recombination and polymorphism and the high density of coding sequence in regions showing reduced heterozygosity-to-divergence ratios is background selection, a phenomenon whereby the removal of deleterious mutations reduces polymorphism at linked sites [1]. To address this possibility, we calculated Fay and Wu's *H* [56] for 10-kb windows across the genome using only sites with a coverage of five alleles and windows not located in extended regions of reduced heterozygosity near the distal and proximal ends of chromosome arms (Materials and Methods). Hitchhiking effects of beneficial mutations are expected to cause an excess of high-frequency derived alleles (and a more-negative *H* statistic) relative to neutral theory predictions, while background selection predicts no such excess [1,72]. We compared the average *H* statistic for regions of the genome showing four or more consecutive 10-kb windows with an HKA-like test of *p* < 0.01 versus 10-kb windows from the rest of the genome. For each chromosome arm, the *H* statistic was significantly more negative in windows showing a reduced heterozyogsity-to-divergence ratio (Mann Whitney *U*, *p* < 10^−4^ for each arm), which strongly supports the proposition that hitchhiking effects of beneficial variants is a major cause of the fluctuations in heterozygosity across the genome. Note, however, that this analysis does not rule out a contribution of background selection [1].

#### Unusual genomic regions and the biology of recent selection.

**Figure 5:**
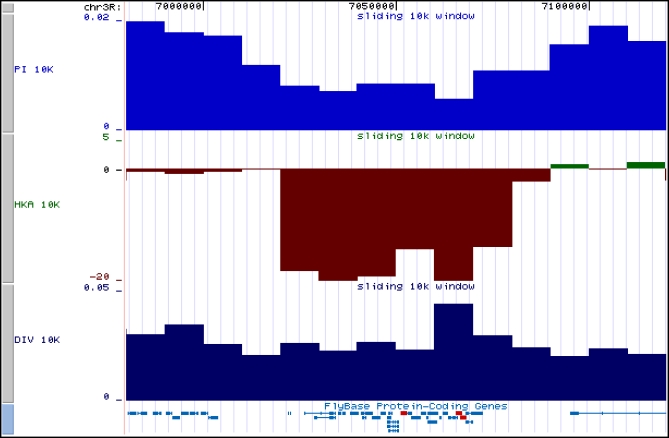
Snapshot of UCSC Browser Tracks in a Genomic Region Showing Significantly Reduced Heterozygosity Relative to Divergence Nucletotide π (blue, labeled “PI 10K”) and *div* on the D. simulans lineage (black), labeled “DIV 10K” in 10-kbp windows are plotted every 10 kbp. χ^2^[-log(*p*)] (green, labeled “HKA 10K”) as a measure of deviation (+ or −) in the proportion of polymorphic sites in 10-kbp windows is plotted every 10 kbp (see Materials and Methods). The genes *scpr-A*, *scpr-B*, and *scpr-C* exhibit high levels of expression in the testes and are indicated in red.

Several large genomic regions (on the order of 20 to 400 kb) showed severely reduced polymorphism. We have established University of California at Santa Cruz Genome Browser tracks (http://rd.plos.org/pbio.0050310) reporting (for nonoverlapping 10-kb windows) π_nt_, polarized nucleotide divergence, coverage, and signed log_10_ of HKA *p*-values (Datasets S16–S20) to facilitate investigation of these regions and promote further investigation of polymorphism and divergence across the D. simulans genome. As an example, Figure 5 shows a Genome Browser snapshot from an unusual region on *3R* (as indicated by large, negative HKA *p*-values) containing 23 genes, including three testis-biased genes, *scpr-A*, *scpr-B*, and *scpr-C*, which are located near the center of the region.

To investigate whether particular biological functions were more likely to be associated with genomic regions showing reduced polymorphism (relative to divergence), we used the genes encompassed by “hitchhiking” windows (*n* = 880 genes for ≥ two 10-kb windows and *n* = 728 genes for ≥ four windows) to search for overrepresented gene ontology (GO) terms (Materials and Methods). The most obvious trend (Table S7) was the frequency of GO terms associated with the nucleus and transcription, which were also common in the McDonald-Kreitman × GO analysis (see below) [4]. This trend supports the proposition that genomic regions of reduced heterozygosity are caused by the spread of beneficial mutations and suggests that biological functions that are targets of recent selection also tend to be targets of recurrent directional selection. Moreover, these patterns suggest that important agents of directional selection are likely related to chronic biological conflict such as meiotic drive, segregation distortion, sexual selection, or host-pathogen/parasite interactions.

#### Regions of strong linkage disequilibrium.

A genomic region that has experienced the recent spread of a strongly favored allele to intermediate frequency should not exhibit a major reduction of heterozygosity. Nevertheless, such regions should show strong linkage disequilibrium, because a single haplotype may constitute a significant proportion of sampled chromosomes. Although the average sample size per base in the D. simulans syntenic assembly (*n* = 3.9) is too small for generating reliable estimates of pairwise correlations among polymorphic sites, the high levels of nucleotide polymorphism and relatively low levels of linkage disequilibrium in this species [73,74] suggest that unusual regions of strong linkage disequilibrium spanning many kilobases could be detectable in our data. We investigated the variance of pairwise nucleotide differences [75,76] across the D. simulans genome using 150-kb overlapping windows (Materials and Methods). Because the mean and variance of pairwise differences showed the expected positive correlation, we used the coefficient of variation (CV) of heterozygosity to summarize the magnitude of large-scale, multilocus linkage disequilibrium for each window (Figure S3). Use of the “chimeric” *SIM4/6* assembly may reduce our power to detect unusual genomic regions but should not lead us to mistakenly identify such regions. At least two salient points emerged from this analysis. First, large regions of the genome showing a severely reduced heterozygosity-to-divergence ratio, such as the tip of the *X* chromosome, tend to have high levels of linkage disequilibrium. Second, some regions of the genome showing unremarkable HKA *p*-values nevertheless have unusually high linkage disequilibrium. Such regions may be candidates for recent selective spread of extended haplotypes. However, several regions showing high linkage disequilibrium are adjacent to regions showing significantly reduced polymorphism. This suggests that such regions are generated by hitchhiking effects of fixed or high-frequency beneficial alleles [77,78] rather than beneficial mutations, which are currently at intermediate frequency on their sojourn through the population.

#### Reduced polymorphism associated with colonization.

D. simulans probably originated in East Africa or Madagascar and recently colonized the rest of the world in association with humans [16]. Lower nucleotide polymorphism in recently established versus “ancient” populations is consistent with this scenario [79–82]. However, directional selection could favor certain alleles in recently established populations, thereby resulting in a further reduction of polymorphism beyond those due to demographic effects [83–85]. To detect such effects, we used 10-kb nonoverlapping windows of the ratio of United States/(Africa + Madagascar) π_nt_ to identify regions of the genome showing a disproportionate reduction of variation in the US sample (Materials and Methods).

Consistent with previous results [79–81], we found the US sample to be significantly less polymorphic than the Africa + Madagascar sample for all chromosome arms (*p* < 0.001). Variation in US genomes is largely a subset of the variation in the Old World genomes. The reduction of polymorphism in the US versus non-US sample is heterogeneous across chromosomes. Although all chromosomes are different from one another (*p* < 0.05), the *X* is clearly the most unusual (Table S8), supporting the finding that recently established populations are relatively depauperate of *X*-linked variation [19,86].

Several genomic regions (Tables S9 and S10) show substantial stretches of disproportionately reduced US heterozygosity. The most significant genomic region, which is located on the *X* chromosome, spans over 100 kb and has severely reduced heterozygosity in the US sample. One interesting gene in the region, *CG1689* (*lz*), is associated with several functions, including defense response and spermatheca development. Another interesting region (chromosome arm *2L*) contains the PI kinase *Pi3K21B*. A related gene was recently shown to be associated with diapause variation in natural D. melanogaster populations [87]. Table S11 shows the GO terms that are significantly overrepresented in significant regions (not Bonferroni corrected), many of which are associated with protein metabolism. Of note is the highly significant term “transmission of nerve impulse,” which is consistent with selection associated with insecticides [88] in recently established populations. Inferences regarding recent selection in D. simulans are weakened by the small sample size, large physical scale of significant regions, and the absence of explicit demographic models in the analysis. Additional data and analyses will be required to address these issues more fully.

### Lineage Effects on Divergence

Several factors can generate lineage differences in divergence. For example, higher divergence in a lineage (relative to the lineage of its sister species) could be due to higher mutation rates, shorter generation times, or stronger directional selection. Investigating which classes of mutations or functional elements tend to show different levels of divergence in two lineages can inform our understanding of the causes of rate variation.

Previously collected data from coding regions suggest that D. melanogaster evolves faster than D. simulans [89,90]. We found a similar pattern in that *dN* and *dS* are greater in D. melanogaster (median = 0.0045 and 0.0688) than in D. simulans (median = 0.0036 and 0.0507) (Table 1 and S3). This pattern has been interpreted as reflecting the reduced efficacy of selection against slightly deleterious variants in D. melanogaster, supposedly resulting from its smaller effective population size relative to D. simulans [89]. However, a different pattern is observed on a genome-wide scale, as median D. simulans divergence (50-kb windows; 0.025), though only slightly greater than D. melanogaster (50-kb windows; 0.022), is consistently greater across a large proportion of windows (Wilcoxon sign rank test, *p* = 1.8 × 10^−275^). We consider the genomic faster D. simulans finding as provisional due the potential biases associated with D. melanogaster-centric alignments. For example, genomic regions that are evolving quickly only in D. melanogaster may drop out of the *D. melanogaster–D. yakuba* alignment, whereas regions evolving quickly only in D. simulans may be retained because of the relatively short *D. melanogaster–D. simulans* branch. Analysis of rate variation across site types (Table 1 and Table S3) reveals a more complex pattern. For example, D. simulans shows greater divergence than D. melanogaster for intergenic, intron, and 3′ UTR sites, whereas D. melanogaster shows greater divergence than D. simulans for 5′ UTRs, nonsynonymous sites, and synonymous sites.

### Adaptive Protein Evolution

A decades-old issue in population genetics is the extent to which directional selection determines protein divergence. Several analytic strategies for investigating the prevalence of adaptive protein divergence between closely related species have been proposed (reviewed in [91]). Here we focused on two approaches. First, we used comparisons of synonymous and nonsynonymous polymorphic and fixed variants in individual genes to test the neutral model. Second, we identified proteins that show very different divergence estimates in D. simulans versus D. melanogaster.

#### Population genetic analysis of recurrent adaptive protein evolution.

McDonald and Kreitman [4] proposed a test (hereafter, MK test) that contrasts the numbers of polymorphic versus fixed/nonsynonymous versus synonymous variants to detect non-neutral protein evolution. The test is based on the neutral theory prediction that the ratio of the number of nonsynonymous-to-synonymous polymorphisms should be similar to the ratio of the number of nonsynonymous-to-synonymous fixations. Recurrent directional selection is expected to result in an increased ratio of nonsynonymous-to-synonymous fixations. We carried out MK tests out for all genes that showed *n* > 6 for each of polymorphisms, fixations, synonymous variants, and nonsynonymous variants (Dataset S1). The filtered data set of unpolarized MK tests contained 6,702 genes, of which 1,270 (19%) were significant (in the direction of adaptive evolution) at the 0.05 critical value and 539 (8%) genes were significant at a 0.01 critical value. Given that MK tests can only detect directional selection when multiple beneficial mutations have fixed, these results provide a conservative view of the prevalence of adaptive protein divergence. There was a slight enrichment of significant unpolarized MK tests on the autosomes relative to the *X* chromosome (Fisher's Exact test, *p* = 0.0014). However, conclusions regarding the incidence of directional selection on autosomes versus the *X* chromosome should be tempered by the fact that the average numbers of polymorphic and fixed variants per gene may differ between the two types of chromosomes, which affects the power of the MK test to reject neutrality. We observed no enrichment of significant tests in regions of the *X* chromosome hypothesized to experience greater versus lower rates of crossing over. Several of the most highly significant MK test statistics are from genes with known functions and in many cases, known names and mutant phenotypes. More generally, among the genes with no associated GO term, a smaller proportion had significant unpolarized MK tests compared to the proportion for genes associated with one or more GO terms (0.16 versus 0.20, *p* = 3 × 10^−5^).

Included among the most highly significant genes in the unpolarized MK tests (Table S12) were several with reproduction-related functions. For example, the sperm of males carrying mutations in *Pkd2* (*CG6504*), the gene with the smallest MK *p*-value in the genome, are not properly stored in females, suggesting sperm–female interactions (perhaps associated with sperm competition) as a possible agent of selection [92,93]. Other examples include *Nc* (*CG8091*), which plays a role in sperm individualization [94]; *Acxc* (*CG5983*), a sperm-specific adenylate cyclase [95]; and *Dhc16F* (*CG7092*), which is a component of the axonemal dynein complex (suggesting a possible role of selection on sperm motility).

For polarized MK tests, we used the D. yakuba genome to infer which fixed differences between D. simulans and D. melanogaster occurred along the D. simulans lineage (Materials and Methods). These fixations were then compared to D. simulans polymorphisms. This reduced, filtered dataset contained 2,676 genes of which 384 (14%) and 169 (6%) were significant at the 0.05 and 0.01 levels, respectively (deviating in the direction of adaptive evolution; Datasets S1). Twenty-three genes showed evidence of a significant (*p* < 0.05) excess of amino acid polymorphism, of which the five that were significant at *p* < 0.01 are presented in Table S13. We found no evidence of more recurrent, adaptive protein evolution on the *X* chromosome, as significant polarized MK tests were not more common for *X*-linked versus autosomal genes (Fisher's Exact test, *p* = 0.74).

Table S14 lists the genes associated with the smallest *p*-values in the polarized MK tests. As expected, there was considerable overlap between the most highly significant genes in the polarized and unpolarized analyses. However, some genes are highly significant in the polarized analysis, but not significant in the unpolarized analysis. For example, *Pvr* (*CG8222*) plays a role in male genitalic development (in addition to the roles noted in Table S14) in D. melanogaster [96]. Male genitalic traits evolve very quickly in *Drosophila* (e.g., [97]), presumably due to some form of sexual selection. *Pvr* thus becomes an attractive candidate gene for investigating the molecular basis of genitalic divergence between D. simulans and its relatives. Another interesting gene is *Gap1* (*CG6721*), which can act as a modifier of minichromosome transmission in D. melanogaster [98], suggesting a possible role in normal chromosome segregation and potentially meiotic drive. Many proteins under recurrent directional selection, such as nuclear pore and cytoskeleton components, have fundamental and diverse cell biological functions. A naïve view would be that pleiotropy associated with mutations in such proteins would be so ubiquitous that rapid adaptive evolution would be unlikely. The genomic data suggest that this view is incorrect.

#### Adaptive protein evolution and gene function.

We investigated the broader biological basis of adaptive protein evolution by determining whether certain GO terms are overrepresented among the genes found to be significant (*p* < 0.05) in unpolarized (Table S15) or polarized (Table S16) MK tests. The unpolarized analysis revealed 26 cellular components, 40 molecular functions, and 96 biological processes significantly enriched for genes under recurrent directional selection. Of particular note among the significant cellular function terms were chromosome, heterochromatin, nuclear envelope, nuclear pore, and polytene chromosome chromocenter, all of which showed *p* < 0.001. Molecular function terms that were enriched (*p* < 0.001) among genes with significant MK tests included adenlyate cyclase activity, chromatin binding, glucose transporter activity, histone methlytransferase activity, lipase activity, microtubule motor activity, and ubiquitin-specific protease activity. Finally, the biological processes terms with *p* < 0.001 were establishment/maintenance of chromatin architecture, female meiosis chromosome segregation, fusome organization/biogenesis, histone methylation, mRNA processing, regulation of cell growth and size, protein deubiquitination, and reproduction.

The polarized analysis revealed eight cellular components, 17 molecular functions, and 47 biological processes that were significant (we use *p* < 0.05, because there were fewer data for each polarized test), including actin binding, glucose transporter activity, ubiquitin-specific protease activity, amino acid biosynthesis, cell motility, cytoplasm and cytoskeleton organization and biogenesis, mRNA processing, and protein import into nucleus.

Overall, biological functions that appear to be under particularly frequent directional selection include those regulating chromosome biology (including motor proteins and chromatin regulation), those regulating movement of material between nucleus and cytoplasm, and those involved in meiosis and reproduction. These findings support speculation based on small datasets [99,100] that intragenomic conflicts relating to gametogenesis may be a major cause of adaptive evolution in *Drosophila*. Sperm competition, sperm-female interactions, or cytoplasmic parasites [101–103] could also result in directional selection on phenotypes related to spermatogenesis. The data and analyses presented here motivate comprehensive investigation of the functional biology of adaptively evolving proteins in D. simulans and the role of such proteins in the evolution of reproductive isolation.

#### Adaptive protein evolution and gene expression.

We used several published gene expression experiments (Materials and Methods) to investigate whether the proportion of genes showing significant MK tests in a given expression category was significantly greater than expected by chance (Table S17). The strongest result was that genes primarily expressed in males are more likely to be under recurrent directional selection, which is consistent with our aforementioned results from MK tests and previously reported results from smaller datasets [104]. We also found evidence that genes expressed primarily in females are enriched for significant MK tests, although only in the polarized analysis. The finding that both male- and female-biased genes are enriched for adaptively evolving proteins supports the idea that antagonistic male–female interactions [105] may drive protein divergence. However, we found no evidence that genes expressed in the sperm-storage organs of mated females are more likely to be under recurrent directional selection than a random sample of genes.

#### Adaptive evolution and protein–protein interactions.

We used published data on *Drosophila* protein–protein interactions (Materials and Methods) to ask whether proteins showing evidence of recurrent directional selection (based on the MK test) are more likely to interact physically with other such proteins. We found no significant genomic association between protein interactions and positive selection. However, there were interesting individual cases in which interacting proteins appear to have diverged under positive selection. For example, as noted here and in previous work [106], nuclear pore components appear to be frequent targets of adaptive evolution. Another interesting case is the *Nc* gene, which has one of the most significant unpolarized MK tests in the genome. The Nc protein, which has several roles including sperm individualization [94], may physically interact with products of at least eight other genes (*Ice*, *Laminin A*, *tramtrack*, *BTB protein-VII*, *Apaf-1 related killer*, *Dodeca satellite binding protein 1*, *CG4282*, and *CG6767*; see [107]). Annotations associated with these proteins include sperm individualization and chromatin condensation, assembly, or disassembly. All four of the eight genes for which we could carry out an unpolarized MK test (*LamininA*, *Apaf-1 related killer*, *Dodeca satellite binding protein 1*, and *CG4282*) rejected the neutral model. These data suggest a history of selection on the molecular components of sperm individualization and differentiation and provide yet further evidence that male reproductive functions are frequent targets of directional selection in *Drosophila*. The causes of such selection are still unclear, but could include gametic selection in *Drosophila* males [108,109], exclusion of cytoplasmic parasites during spermatogenesis [101,103], or selection on aspects of sperm morphology associated with sperm competition or sperm–female interactions [110]. The role of physically interacting, adaptively evolving proteins that function in spermatogenesis for hybrid sterility remains an intriguing, open question.

#### Proteins showing increased divergence.

Genes that show relatively low nonsynonymous divergence in D. yakuba and D. melanogaster but high nonsynonymous divergence in D. simulans may have a history of adaptive evolution in D. simulans. Similarly, genes showing elevated nonsynonymous divergence only in D. melanogaster may have a history of adaptive evolution in this species. Although this approach does not exploit the D. simulans polymorphism data, it permits investigation of genes that show little polymorphism due to hitchhiking effects or low sequence coverage. Although directional selection is the most plausible explanation for a lineage-specific rate increase, a change in the neutral mutation rate could also lead to a rate increase. However, three results support the proposition that an inflated lineage-specific *dN* is associated with natural selection. First, the median relative rate χ^2^ statistic for *dN* is greater for genes with significant unpolarized MK tests (1.91) than for genes with nonsignificant test (1.69) (Mann-Whitney *U*, *p* < 1 × 10^−20^). Second, of the 352 genes showing significant (*p* < 0.05) *D. simulans dN* rate accelerations and which had sufficient data for polarized MK tests (see below), 28% (99) of the tests were significant (*p* < 0.05). Of the 2,301 nonsignificant genes, only 12% (285) had significant polarized MK tests. Finally, the median synonymous π_nt_/*D. simulans dS* for genes that showed significant *D. simulans dN* rate increases (*n* = 743, median = 0.46) is dramatically lower than the median for nonsignificant genes (*n* = 9300, median = 0.63, Mann-Whitney *U*; *p* = 2.1 × 10^−23^), which is consistent with recurrent selection inflating protein divergence while reducing heterozygosity at closely linked synonymous sites.

The genes (*n* = 25) showing the largest test statistics consistent with lineage-specific protein acceleration are shown in Tables S18 and S19 for D. simulans and D. melanogaster, respectively. Many of the top 25 genes in each lineage are named and associated with considerable functional information. Thus, genes with important functions may still be subject to strong, lineage-specific rate acceleration.

#### Accelerated protein divergence and gene function.

We used permutation tests to gain a broader view of enrichment of particular protein functions with *dN* χ^2^ test statistics in D. simulans (Table S20). The GO terms with *p* < 0.001 and *n* > 10 genes include nuclear envelope, nuclear pore, amino acid-polyamine transporter activity, ubiquitin-specific protease activity, protein deubiquitination, and protein import into the nucleus. Results from a comparable analysis of D. melanogaster protein evolution are shown in Table S21. Using the same criteria of *n* > 10 genes and *p* < 0.001, we find only FAD binding and antimicrobial humoral response GO terms. However, several other GO terms are significant (e.g., choline dehydrogenase activity, endopeptidase inhibitor activity, oxidoreductase activity, and dosage compensation) and worthy of further investigation in D. melanogaster.

### Adaptive Evolution of Noncoding Elements

The same logic originally proposed in the MK test using nonsynonymous and synonymous variation can be extended to any setting in which variant types can be categorized, a priori. We tested variation in individual noncoding elements (introns, UTRs, and intergenic sequences) relative to variation at tightly linked synonymous sites (Materials and Methods) using the same criteria described for the MK tests; we present only polarized analyses (Datasets S2–S5). The proportion of tests (Materials and Methods) that rejected (*p* < 0.05) the null model for 5′ UTR, 3′ UTR, intron, and intergenic sites are 0.13, 0.13, 0.12, and 0.17, respectively. However, unlike the case for the nonsynonymous versus synonymous polarized MK tests, of which only 6% of the significant tests deviated in the direction of excess polymorphism (relative to synonymous sites), a much greater proportion of noncoding MK tests deviated in this direction—0.13, 0.24, 0.28, and 0.28 for 5′ UTR, 3′ UTR, intron, and intergenic regions, respectively. Thus, the proportion of noncoding elements showing evidence of adaptive evolution for 5′ UTR, 3′ UTR, intron, and intergenic sites is 0.12, 0.10, 0.08, and 0.12, respectively, which is similar to the proportion of coding sequences inferred (by polarized MK tests) to be under direction selection (0.14). It would be tempting to conclude from this result that intergenic variants are as likely to be under directional selection as nonsynonymous variants. However, such an interpretation ignores the fact that the number of variants per element for each MK test is much greater for intergenic sequence (median = 87) compared to the numbers for coding regions (median = 42), 5′ UTRs (median = 34), 3′ UTRs (median = 35), or introns (median = 64). Thus, there is more power to reject the neutral model for intergenic sequence and introns than for exonic sequence. The fact that MK *p*-values are significantly negatively correlated with the total number of observations per test is consistent with this explanation. There was no evidence of different proportions of significant versus nonsignificant tests for *X*-linked versus autosomal elements.

Tables S22–S24 report data from the ten most highly significant MK tests (average coverage > 2) indicative of directional selection on 5′ UTRs, 3′ UTRs, and intron sequences, respectively. Among the most unusual 5′UTRs are those associated with genes coding for proteins associated with the cytoskeleton or the chromosome, categories that also appeared as unusual in the MK tests on protein variation. Two of the top-ten 3′ UTRs are associated with the SAGA complex, a multi-subunit transcription factor involved in recruitment of RNA Pol II to the chromosome [111]. Among the extreme introns, two are from genes coding for components of the ABC transporter complex and two are from genes coding for centrosomal proteins, again pointing to the unusual evolution of genes associated with the cytoskeleton and chromosome structure and movement. As previously noted, a large number of significant UTRs deviate in the direction of excess polymorphism (relative to synonymous mutations). Given the potential importance of the UTRs in regulating transcript abundance and localization, translational control, and as targets of regulatory microRNAs [112], such UTRs could be attractive candidates for functional investigation. Contingency tests of significant versus nonsignificant MK test for amino acids versus each of the noncoding elements yielded *p*-values of 0.65, 0.04, and 0.07 for 5′ UTRs, 3′ UTRs, and introns, respectively. Thus, there is weak evidence that genes under directional selection on amino acid sequences tend to have 3′ UTRs and introns influenced by directional selection as well.

### Whole-Genome Analysis of Polymorphic and Fixed Variants

Up to this point, our analyses have investigated various attributes of polymorphism and divergence based on windows or genes. An alternative approach for understanding the causes of variation and divergence is to analyze polymorphism and divergence across site types. Table 2 shows whole-genome counts of polymorphic and polarized fixed variants for UTRs, synonymous sites, nonsynonymous sites, introns, and intergenic sites. We also provide data for polarized, synonymous preferred or unpreferred variants. Almost all preferred versus unpreferred codons in D. melanogaster end in GC versus AT, respectively [113]; thus, preferred versus unpreferred codons can be thought of as GC-ending versus AT-ending codons.

**Table 2:**
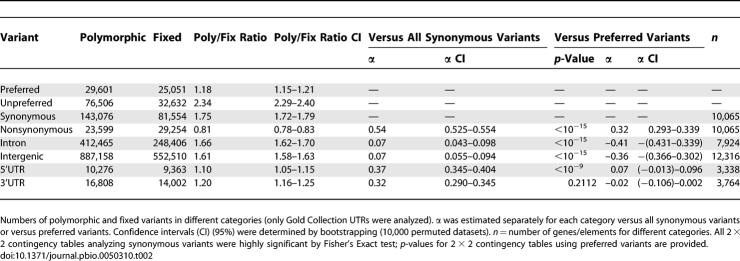
Whole-Genome Counts of Polarized Polymorphic and Fixed Variants

Nonsynonymous sites showed the smallest ratio of polymorphic-to-fixed variants, which is consistent with the MK tests and supports the idea that such sites are the most likely to be under directional selection. Nonsynonymous polymorphisms also occur at slightly lower frequency than do noncoding variants (Table S25). Synonymous sites have the highest ratio of polymorphic-to-fixed variants, which supports the previously documented elevated ratio of polymorphic-to-fixed unpreferred synonymous variants in D. simulans [89]. The confidence intervals of the ratio of polymorphic-to-fixed variants among site types are nonoverlapping with the exception of intron and intergenic sites. If preferred synonymous mutations are, on average, beneficial [89,114], then the smaller polymorphic-to-fixed ratio for nonsynonymous and UTR variants versus preferred variants implies that a large proportion of new nonsynonymous and UTR mutations are beneficial. Using similar reasoning, the data in Table 2 suggest that directional selection plays a larger role in nonsynonymous and UTR divergence compared to intron and intergenic divergence [20,115,116]. These conclusions are consistent with estimates of α [11,117], the proportion of sites fixing under directional selection (assuming that synonymous sites are neutral and at equilibrium) for different site types.

### Base Composition Evolution

Determining the relative contributions of various mutational and population genetic processes to base composition variation and inferring the biological basis of selection on base composition remain difficult problems. Much of the previously published data on base composition variation in D. simulans have been from synonymous sites [55,89,90,118]. Several lines of evidence [55,89,90,113,118] suggest that on average, preferred codons have higher fitness than unpreferred codons, with variation in codon usage being maintained by AT-biased mutation, weak selection against unpreferred codons, and genetic drift [23,114]. However, the possibilities of nonequilibrium mutational processes and/or natural selection favoring different base composition in different lineages have also been addressed [119]. The D. simulans population genomic data allow for a thorough investigation of the population genetics and evolution of base composition for both coding and noncoding DNA [59,120]. The analyses discussed below use parsimony to polarize polymorphic and fixed variants. Complete genomic and gene-based data are available as Datasets S9 and S10.

#### Synonymous sites.

Previous reports suggested that D. simulans synonymous sites are evolving towards higher AT content, although the excess of AT over GC fixations is small [55]. That trend was confirmed in this larger dataset; there are many more ancestral preferred codons that have fixed an unpreferred codon (coverage classes four–six, *n* = 21,156) in D. simulans compared with ancestral unpreferred codons that have fixed a preferred codon (coverage classes four–six, *n* = 15,409). Furthermore, the population genomic data also support previous reports [89] that *D. melanogaster* synonymous sites are becoming AT-rich at a faster rate than D. simulans synonymous sites (Table S26), contributing to the higher median *dS* in D. melanogaster (0.069) compared to D. simulans (0.051, Wilcoxon Signed Rank, *p* < 0.0001).

The data also support previous reports [89] in that 2 × 2 contingency tables of polymorphic versus fixed, preferred versus unpreferred variants are highly significant for the *X* chromosome and the autosomes (Table S27). Under the mutation-selection-drift model [89,114], this pattern has been interpreted as reflecting a disproportionate contribution of borderline deleterious unpreferred variants to the synonymous polymorphism in D. simulans. This model predicts that unpreferred polymorphisms should occur at lower average frequency than preferred variants. Indeed, contingency tests (coverage-five sites) showed that this is the case (Table S28).

Previous results showing higher levels of codon bias for the *X* chromosome versus autosomes suggest the possibility of more effective selection against *X*-linked unpreferred variants [58]. The population genomic data revealed that the ratio of preferred-to-unpreferred fixations was not significantly different for the *X* versus autosomes (coverage classes four and five *p*-values = 0.28 and 0.11, respectively), which shows that rates of codon bias evolution are not detectably different for *X* chromosomes and autosomes. However, two additional aspects of the data support the idea that selection on codon bias differs between the *X* chromosome and the autosomes. First, the ratio of unpreferred-to-preferred polymorphisms is significantly smaller for the *X* chromosome compared to the autosomes (coverage classes four and five, *p*-values < 0.0001 and 0.003, respectively). Second, unpreferred polymorphisms occur at significantly lower frequency on the *X* chromosome than on the autosomes (Table S28; coverage five sites, *p* = 0.0014). Both of these observations are consistent with an increased efficacy of natural selection against *X*-linked unpreferred mutations [58].

Finally, we note that the ratio of preferred-to-unpreferred fixations in D. simulans was slightly higher (*p* = 0.002) among the genes associated with a significant polarized MK test (0.83) compared to those associated with a nonsignificant test (0.75). This is consistent with the notion that amino acid variants experiencing directional selection are more likely to fix if they are associated with preferred codons (Table S29).

#### Noncoding sites.

Because selection on codon bias occurs only in protein-coding regions, comparisons of base composition variation in protein-coding versus noncoding regions can serve to rule out some explanations for codon bias or point to general explanations for base composition variation that are unrelated to selection on codons.

Although synonymous sites are evolving toward higher AT content in D. simulans, analysis of noncoding sites clearly demonstrate that GC fixations are significantly more common than AT fixations (coverage classes two–six; 277,005 GC versus 218,302 AT). This observation is inconsistent with predictions of equilibrium base composition (binomial probability, *p* < 1 × 10^−6^). The D. simulans genome is becoming more GC-rich, as the large GC fixation bias for intron and intergenic sites greatly outweighs the countervailing AT fixation bias at synonymous sites (Table S30).

To gain further insight into base composition evolution, we investigated polymorphic and fixed AT versus GC variants in intergenic and intron DNA (coverage five sites in Table 3). We found that the ratio of polymorphic-to-fixed AT variants was much larger than the corresponding ratio for GC variants for both intron and intergenic sequence. These data are consistent with selection favoring GC over AT mutations; although if this is the case, such GC mutations are apparently favored significantly less strongly than preferred mutations, as the polymorphic-to-fixed ratio for GC is much higher for intron/intergenic variants than for synonymous variants. Alternatively, biased gene conversion favoring GC could increase the frequency of GC variants. Although configurations of polymorphic versus fixed variants were generally similar for intron and intergenic DNA (Table S30), autosomal data from coverage-six sites (Dataset S9) suggest that the ratio of polymorphic-to-fixed AT variants is greater for introns (3.12) than for intergenic DNA (2.76; χ^2^ = 30.4, *p* = 3 × 10^−8^).

**Table 3:**
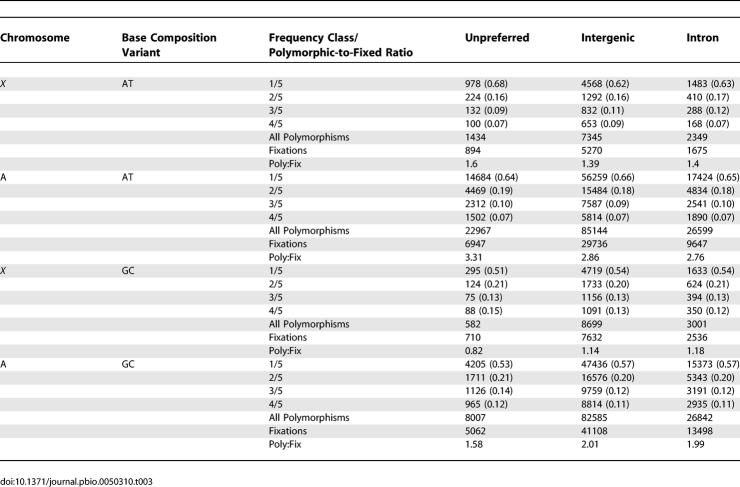
Counts (Frequencies) of Variants for the *X* and Autosomes (A) for Sites with Coverage of Five D. simulans Alleles

We further investigated base composition variation by comparing the frequency spectrum of derived GC versus AT polymorphisms in noncoding DNA for coverage-five sites. For the *X* chromosome and the autosomes, intergenic and intron GC polymorphisms occurred at significantly higher average frequencies than AT polymorphisms (Table 3; χ^2^, *p* < 10^−4^ for all tests). As expected, if gene conversion favoring GC variants contributes to their higher frequency and if most gene conversion occurs during female meiosis, the *X* chromosome has significantly higher frequencies of GC polymorphisms (Table 3, χ^2^, *p* < 10^−4^). We also compared GC-to-AT ratios for the *X* versus autosome polymorphisms in coverage-six sites (sites at which a base was called in all six D. simulans syntenic assemblies). In agreement with predictions for biased gene conversion, the ratio of GC-to-AT polymorphisms was greater on the *X* chromosome than on the autosomes for each frequency class (Table S31), although frequency class 1 was the only one that individually had a significantly greater GC-to-AT ratio on the *X* (1.06) than on the autosomes (0.89) (χ^2^ = 25.8, degrees of freedom = 1). Overall, these results support a role for biased gene conversion favoring GC or more-effective selection favoring GC on the *X*.

The observation that ancestral GC content is negatively correlated with D. simulans divergence (50-kb windows; described above) may be understood as a consequence of the fact that genomic regions having higher ancestral AT content have more, new GC mutations that may be favored by genic selection or biased gene conversion compared with regions that ancestrally were more GC rich. The question remains as to why fewer preferred codons have fixed compared to unpreferred codons given that the former may be favored by genic selection due to translational efficiency or accuracy, as well as by biased gene conversion. One possibility is that ancestral codons were so enriched for preferred variants that the mutation rate to unpreferred variants has outweighed selection against such variants. Alternatively, selection on base composition could be stronger for noncoding than for coding sequence. However, these interpretations do not help us explain the basic conundrum: the D. simulans genome is far from base composition homogeneity and stationarity for many site types. The biological explanation for evolving base composition remains a mystery.

### Conclusions and Prospects

The genomic analysis of polymorphism and divergence based on alignments to a reference sequence is poised to become a central component of biological research. Here we have demonstrated that such analysis can be based on high-quality whole-genome syntenic assemblies from light shotgun sequence data; accounting for variable coverage and data quality is fundamentally important. Several, noteworthy new results have been reported here. First, our genome-level investigation of adaptive protein evolution has revealed a large number of proteins and biological processes that have experienced directional selection, setting the stage for a general analysis of functional protein divergence under selection in *Drosophila*. Second, we identified several UTRs, introns, and intergenic sequences showing the signature of adaptive evolution. The functional biology of such noncoding elements and their connections to adaptive protein and gene expression evolution is open to investigation. Third, D. simulans populations exhibit large-scale chromosomal patterning of polymorphism and divergence that is poorly explained by current genome annotations. Variation in recombination rates across chromosomes may contribute to these patterns. Fourth, the population genetics of the *X* chromosome differs in several ways from that of the autosomes. It evolves faster, harbors less polymorphism, and shows a different spatial scale of variation of polymorphism and divergence compared to the autosomes. Finally, base composition is evolving in both coding and noncoding sequences, for reasons that are as of yet unclear. This project is, in many ways, a first step toward population genomics in general, and in the *Drosophila* model specifically. For example, the average number of alleles sampled per base is too small for investigating many interesting properties of variation. Some genomic regions have been excluded due to low coverage, their repetitive nature, or very high divergence from D. melanogaster. Many aspects of biological annotation have not been investigated here, and many new *Drosophila* annotations will be produced in the near future as comparative and functional annotations of the D. melanogaster genome move forward. Nevertheless, the data are a stunningly rich source of material for functional and population genetic investigation of D. simulans polymorphism and divergence. It will be interesting to compare the processes determining polymorphism and divergence in D. simulans to those controlling variation in D. melanogaster (http://www.dpgp.org) and in other species, such as humans. Such comparisons are likely to result in new insights into the genetic, biological, and population genetic factors responsible for similarities and differences among species in the genomic distribution of sequence variation.

## Materials and Methods

### *Drosophila* stocks.

D. simulans 4 (males and females). This strain was established by ten generations of sibling mating from a single, inseminated female collected by D. Begun in the Wolfskill orchard, Winters, California, USA, summer 1995.

D. simulans 6 (males and females). This strain was established by ten generations of sibling mating from a single, inseminated female collected by D. Begun in the Wolfskill orchard, Winters, California, summer 1995.

*D. simulans w^501^*(males and females). This strain carries a white (eye color) mutation. It has been in culture since the mid 20th century, likely descended from a female collected in North America. The strain used for sequencing was sib-mated for nine generations by D. Barbash at UC Davis. Libraries for sequencing were prepared from DNA isolated from embryos.

D. simulans MD106TS (males and females). This strain was descended from a single, inseminated female collected by J. W. O. Ballard in Ansirabe, Madagascar on 19 March 1998. It has the *siII* mitochondrial genotype, and was cured of *Wolbachia* by tetracycline. The strain was sib-mated for five generations in the Ballard lab, followed by an additional five generations of sib-mating by D. Begun.

D. simulans MD199S (females). This strain was descended from a single, inseminated female collected by J. W. O. Ballard in Joffreville, Madagascar on 28 March 1998. It has the *siIII* mitochondrial genotype, and probably lost *Wolbachia* infection. The strain was sib-mated for five generations in the Ballard lab, followed by an additional five generations of sib-mating by D. Begun. All-female DNA was made to assist in assembly of the Y chromosome by comparison to mixed-sex libraries of other lines.

D. simulans NC48S (males and females). This strain was descended from a collection by F. Baba-Aissa in Noumea, New Caledonia in 1991. It has the *siI* mitochondrial genotype, and was sib-mated for five generations in the Ballard lab, followed by an additional five generations of sib-mating by D. Begun.

D. simulans C167.4 (males and females). This strain was descended from a collection in Nanyuki, Kenya. It is unusual in that can produce fertile females when hybridized to D. melanogaster. The line used for genome project was obtained from the Ashburner laboratory via D. Barbash, and was subjected to a total of 13 generations of sib- mating.

D. yakuba Tai18E2 (males and females). This strain derives from a single inseminated female captured in 1983 by D. Lachaise in the Taï rainforest, on the border of Liberia and Ivory Coast. This line was sib-mated for ten generations by A. Llopart and J. Coyne. Inspection of 21 salivary gland polytene chromosomes showed no chromosomal rearrangements segregating within the strain. Therefore, Tai18E2 appears homokaryotypic for the standard arrangement in all chromosome arms, save *2R*, which is homokaryotypic for 2*Rn*.

### DNA extraction.

DNA preparations for sequencing all lines except *w^501^* and Tai18E2 were made from adults. *Drosophila* nuclei were isolated following Bingham et al. [121]. For all lines except *w^501^* and Tai18E2, DNA was isolated by phenol/chloroform extraction of nuclei followed by ethanol precipitation. For lines *w^501^* and Tai18E2, embryos were collected using standard procedures [122] followed by DNA isolation on CsCl gradients [121].

### D. yakuba sequencing and assembly.

Sequence data were obtained from paired-end plasmids and fosmids (Table S32) using standard Washington University Genome Sequencing Center laboratory protocols (http://genome.wustl.edu). A highly automated production pipeline using a 384-well format ensured the integrity of the paired-end data.

We determined the nucleotide-level accuracy by reviewing the quality values of the D. yakuba consensus assembly and by comparison to manually edited D. yakuba sequence. More than 97% of the D. yakuba genome sequence had quality scores of at least 40 (Q40), corresponding to an error rate of ≤10^−4^. Further, we extracted reads from two local fosmid-sized regions (68 kb, defined by fosmid-end sequence pairs, one on chromosome *X* and one on chromosome *3L*) of the assembly and reassembled using Phrap (P. Green, unpublished data). The resulting “fosmids” were manually reviewed and edited. Comparison of the sequence to these manually edited regions revealed a high-quality discrepancy rate of 2 × 10^−4^ substitutions and 1 × 10^−4^ insertion/deletion errors, consistent with the above estimates based on consensus base quality. We also found no evidence of misassembly when comparing the WGS assembly to these projects.

Repetitive content was estimated both in D. yakuba and D. melanogaster using RECON to generate the repeat families and RepeatMasker to then identify those repeats in the genomes. The D. yakuba genome was ∼27% repetitive overall (of which ∼2.5% is simple sequence repeats/low complexity sequence) and 8% in the euchromatic portion of the genome. The D. melanogaster genome was ∼11% repetitive overall (of which 2.3% is simple sequence repeats/low complexity sequence) and ∼7% in the euchromatic portion of the genome.

The first step in creating D. yakuba chromosomal fasta files was to align the D. yakuba WGS assembly data against the D. melanogaster genome. D. yakuba supercontigs were artificially broken into 1,000-bp fragments and aligned against the D. melanogaster genome using BLAT [123]. An alignment was defined as “unique” if its best scoring match had a score of at least twice that of its next best scoring alignment. Of the 139.5 Mb of D. yakuba supercontigs that uniquely aligned to the D. melanogaster genome (4.2 Mb of which aligned uniquely to D. melanogaster unlocalized sequence, chrU), only 16 supercontigs totaling 15.1 Mb contained unique assignments to more than one chromosome arm. Eleven of these involved alignments to heterochromatin where only less than ∼5% of the supercontig aligned uniquely to the D. melanogaster genome. These contigs were assigned to either chrU or the heterochromatic portion of the chromosome for cases where the contig aligned to both the heterochromatic and nonheterochromatic portion of the same chromosome. One 200-kb contig had only 6.2 kb that uniquely mapped to the D. melanogaster genome, 3.8 kb mapping to chr2R, and 2.4 kb mapping to chrX. This supercontig was assigned to chrU. The remaining four supercontigs were alignments to chromosome arms *2L* and *2R*, the location of a known pericentric inversion between D. melanogaster and D. yakuba.

The D. yakuba contigs were initially ordered by their position along the assigned D. melanogaster chromosomes. Because there are rearrangements in D. yakuba as compared to D. melanogaster, we allowed one portion of a D. yakuba supercontig to align to one region of a chromosome and the remaining portion to align elsewhere along that chromosome. For example, four supercontigs aligned to both chromosome arms *2L* and *2R*. However, these *2L*/*2R* cross-overs and other interspecific nonlinearities are expected given the known chromosome inversions [124] between D. yakuba and D. melanogaster. This initial ordering for *2L*, *2R*, *3L*, *3R*, and *X* was used as the starting point for manually introducing inversions in the D. melanogaster-ordered D. yakuba supercontigs. The goal was to minimize the total number of inversions required to “rejoin” all D. yakuba supercontigs previously assigned to distant chromosomal regions based on D. melanogaster alignments (L. Hillier, unpublished data). Inversions were only introduced between contigs and not within contigs. Using this process, we created the final chromosomal D. yakuba sequence.

### D. simulans sequencing.

Sequence data were obtained from paired-end plasmids from the various D. simulans strains using standard laboratory protocols (http://genome.wustl.edu). A genomic assembly was also created. We began by generating an ∼4× WGS assembly of *D. simulans w^501^* using PCAP [18]. The *w^501^*contigs were initially anchored, ordered, and oriented by alignment with the D. melanogaster genome in a manner similar to that described above for alignments between the D. yakuba and D. melanogaster genome. The assembly was then examined for places where the *w^501^* assembly suggested inversions with respect to the D. melanogaster assembly. One major inversion was found, confirming the already-documented inversion found by [124]. Six other D. simulans lines (*C167*.*4*, *MD106TS*, *MD199S*, *NC48S*, *SIM4*, and *SIM6*) were also assembled using PCAP with ∼1× coverage. Using the 4× WGS assembly of the *D. simulans w^501^* genome as a scaffold, contigs and unplaced reads from the 1× assemblies of the other individual D. simulans lines were used to cover gaps in the *w^501^* assembly where possible. Thus, the resulting assembly is a mosaic containing the *w^501^* contigs as the primary scaffolding, with contigs and unplaced reads from the other lines filling gaps in the *w^501^* assembly (L. Hillier, unpublished data). The *D. simulans w^501^* whole-genome shotgun assembly can be accessed at GenBank.

### D. simulans syntenic assembly.

The goal was to align unique D. melanogaster reference sequence assembly v4 to orthologous D. simulans sequence. The D. melanogaster genome was preprocessed to soft mask all 24mers that were not unique, as such sequences were not expected to have a discriminating effect during mapping of D. simulans reads. Transposable elements in the reference sequence were also masked.

The D. simulans WGS reads were quality trimmed prior to assembly based on their phred-score derived error probability. These error probabilities were used to trim the read back to the longest contiguous interval with an average probability of error less than 0.005. Each end was then examined and trimmed until its terminal 10 bp had an average probability of error less than 0.005. If the read was less than 50 bp after this process, it was discarded. These criteria resulted in 164,480 discarded reads from a total 2,424,141 reads. See Table S33 for read and trim statistics.

A dynamic programming algorithm was used to create a maximum-likelihood description of the evolutionary path between sequences from the two species with respect to the standard alignment model, which was extended to incorporate the possibility of sequencing error. To improve the accuracy of the alignments, optimal parameters were estimated with respect to the overall expected evolutionary distance between the two species. This was done from a first-pass assembly using the method described in [129]. Because dynamic programming is not feasible on a genomic scale, we determined candidate locations for each read using the MegaBLAST (http://www.ncbi.nlm.nih.gov/BLAST/docs/megablast.html) algorithm. A read was then realigned to each candidate location as a single contiguous alignment using a derivative of the Smith-Waterman algorithm, which was adapted to incorporate the expected divergence and the error probabilities provided by Phred quality scores. Alignments were ranked by score. Reads were considered unambiguously mapped if their alignment covered at least 90% of the sequence and showed more than two high-quality differences between the putative best orthologous location and a possible secondary candidate location. Reads were incorporated into the assembly on a clone-by-clone basis only if both mate-pairs were unambiguously mapped with the proper orientation and appropriate distance from each other.

For each D. simulans line, the aligned reads were coalesced into syntenic contigs using their overlap with respect to the D. melanogaster genome. Note that “overhanging” or unaligned sequence that may represent transposable elements, other repetitive sequence, or highly diverged sequence, was not considered. This “master–slave” multiple alignment contains reads that are aligned “optimally” with respect to the D. melanogaster reference sequence. However, this does not ensure that the reads are optimally aligned with respect to each other. For instance, small, identical insertion or deletion variants may not be mapped to precisely identical locations in all D. simulans reads. To address this problem, the D. melanogaster reference sequence was set aside, and the method of Anson and Meyers [125] was used to optimize the alignment of each component read of each D. simulans line with respect to a *D. simulans–*only consensus sequence. This method, which minimizes the sum of differences between each of the reads and the consensus sequence, belongs to the class of expectation maximization algorithms [125]. The round robin, align-and-update algorithm converges on a consensus sequence and alignment that most parsimoniously describe the differences between each read and the consensus. This has the effect of coalescing deletions and aligning insertions. The end result of the assembly is a multi-tiered alignment with associated quality scores for (i) the trimmed reads, (ii) the assembled sequences within lines, and (iii) a species consensus sequence, all aligned to the D. melanogaster reference sequence. A reference sequence was produced for each D. simulans line by concatenating the syntenically assembled contigs that were padded with respect to the D. melanogaster reference sequence. The result is a set of D. simulans genomes onto which D. melanogaster annotation can be directly mapped.

### Empirical validation of syntenic assembly.

Nine regions, including coding and noncoding DNA, were chosen to cover a range of polymorphism levels as predicted by an early version of the syntenic assembly. These regions were amplified from lines *C167.4*, *MD106TS*, *NC48S*, and *w^501^*, and sequenced at UNC-Chapel Hill High-Throughput Sequencing Facility. Sequences were assembled using Consed; a minimum quality score of 30 was required. Approximately 27,500 bp were sequenced per line. The per-base discrepancy between these sequences and the current syntenic assembly (insertions, deletions, and masked bases omitted) was estimated as 0.00043.

### Alignment of D. yakuba to the D. melanogaster reference sequence.

An orthology map (with respect to the D. melanogaster reference sequence) of D. yakuba assembly (v1.0) was generated by the Mercator program (http://rd.plos.org/pbio.0050310a). The MAVID [126] aligner was run on each orthologous segment in the map. MAVID uses protein-coding hits reported by Mercator to anchor its alignment of each segment. It recursively finds additional anchors and then runs the Needleman-Wunsch algorithm in between the anchors to obtain a single, global alignment of the entire orthologous segment.

### Heterochromatic regions.

These regions were filtered based on manual examination of the density of annotated repetitive sequence in the centromere and telomere proximal regions of the five large arms. The transition from the “typical” euchromatic density of large repeats to the typical “beta heterochromatic” pattern is obvious. The “euchromatic/heterochromatic boundaries” were drawn roughly at the edges of the first annotated gene within each euchromatic arm.

The following regions were excluded from analysis: (i) *X* 1 to 171944 AND 19740624 to END; (ii) *2L* 1 to 82455 AND 21183096 to END; (iii) *2R* 1 to 2014072 AND 20572063 to END; (iv) *3L* 1 to 158639 AND 22436835 to END; and (v) *3R* 1 to 478547 AND 27670982 to END.

### Consensus and quality scores.

The sequence for each line is derived from the multiple alignment of reads to the D. melanogaster reference assembly (v4). For each line and each column (nucleotide position) corresponding to a D. melanogaster base, a likelihood model was used to determine the quality score for each of the four bases. The quality score was calculated as −10log(1 – probability base is correct). To compute the probability a base call is correct, we assume that each read is an observation of a random variable with equal likelihoods for all four bases with some probability of error. From the definition of a phred score, the probability of error for a particular observed call is: 10^(phred score/–10 )^. We assumed that a base in error is equally likely to be any one of the three other bases. Then, for a given position A, Bayes theorem implies the probability (Pr) that the call is correct is

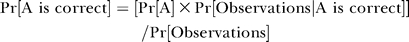



Where Pr[A] = 1/4, Pr[Observations|A is correct] = likelihood of A observations being correct and non-A observations being incorrect, and Pr[Observations] = likelihood of seeing observed values given frequency and error rates.

Quality scores were truncated at 90. The sequences for each line were investigated for regions containing unusually high densities of high-quality discrepancies, which are due to residual heterozygosity, duplication, and erroneous sequence. These regions were filtered from subsequent analysis (see below). For each line, the support for each alternative (A, G, C, and T) at each aligned base was the sum of the qualities, with the highest quality base assigned as the base for that line/position. Implicit in this approach is that a base is called only if the highest quality base has a quality score that is 30 or more greater than that of the next highest quality base. The combined *SIM4/SIM6* consensus was also treated in this manner.

### Filtering of high-quality discrepancies within lines.

Residual heterozygosity within lines or duplications present in D. simulans but not D. melanogaster can lead to regions with excess high-quality discrepancies between reads within lines. We refer to these as single-nucleotide discrepancies. We derived a distribution of the number of discrepancies per site over each chromosome for each D. simulans line. We based the distributions on counts of within-line discrepancies per site in 500-bp windows that had 250-bp overlap. We took the conservative approach of filtering windows in all the lines that fell into the top 0.5% of the distribution in any single line. In other words, a window with a high-quality discrepancy in one line was filtered from the entire dataset, even if the other lines had no discrepancy. Overall, 334,500 base pairs were filtered from the genome. The number of sites filtered for each chromosome arm were 39 kb for *2L*, 86.5 kb for *2R*, 58 kb for *3L*, 73 kb for *3R*, and 78 kb for *X*.

### Inversion on the D. melanogaster lineage.

One large inversion on chromosome arm *3R* distinguishes the two species. Phylogenetic analysis of the cytogenetic data suggested that the inversion fixed in the D. melanogaster lineage [39]. Thus, *D. simulans 3R* is rearranged with respect to the D. melanogaster reference sequence. We used D. melanogaster/D. simulans alignments provided by the UC Santa Cruz Genome Browser to locate the putative breakpoints of the inversion as Chr*3R*: 3874907 and 17560827.

### Features.

All features were defined in the D. melanogaster v4.2 annotation (http://flybase.org). For each gene, the longest isoform (i.e., the isoform the with greatest number of codons) was chosen for analyses. Exons that were not part of the longest isoform were excluded from all feature-based analyses, but were included in window analyses. The analyzed introns correspond to these longest isoforms; all introns were included in window analyses. Intronic sequences within annotated UTRs or that overlapped any coding sequence were excluded. UTRs investigated for this paper were restricted to those inferred from “Gold Collection” genes with completely sequenced cDNAs (http://www.fruitfly.org/EST/gold_collection.shtml). All annotated CDS sequences were used regardless of the associated empirical support. Intergenic regions were defined as noncoding segments between annotated genic regions (UTRs, coding sequence, and noncoding RNAs) regardless of strand. Defined intergenic regions from v4.2 annotation were checked against all known coding and UTR coordinates; any nucleotides that overlapped a genic region were removed from the intergenic set before analysis.

### Defining the D. simulans syntenically aligned gene set.

We established a conservative gene set for analyses (base composition analyses excepted) by including only genes for which the start codon (ATG or otherwise), splice junctions (canonical or otherwise), and termination codon position agreed with the D. melanogaster reference sequence. We took the conservative approach of excluding from the gene-based analysis any gene for which any of the six D. simulans gene models disagreed with the D. melanogaster gene model.

### Insertions and deletions.

Long insertions and deletions (indels) are difficult to identify using only aligned reads. As indel length increases, the likelihood that indels are missed increases because they are either too long or occur near the end of a read, which compromises alignment. Furthermore, indel error probabilities are difficult to estimate. These considerations led us to restrict our analysis to indels of 10 bp or less and to restrict our analysis of divergence to the D. simulans versus D. melanogaster comparison. Variants were classified as insertions or deletions relative to the D. melanogaster reference sequence. The quality score for an insertion was the average quality score of sequence in that insertion; the quality score for a deletion was the minimum of qualities of the two flanking nucleotides. Qualities were determined this way to provide a metric of overall sequence quality in the region of a putative indel, thereby allowing a quantitatively defined cutoff for inferring indel variants; only indels of high quality (over phred 40) were considered in the analysis.

### Estimation and inferences.

Light, variable coverage of each line requires that statistical estimation and inference account for coverage variation. When appropriate (e.g., contingency tables of frequency variation), counts of variants within a coverage category were used. In other estimation and inference settings, the familiar estimators were applied to each coverage class and then averaged, weighting by the proportion of total covered base pairs in the window or other feature.

*Heterozygosity.* The expected nucleotide, insertion, and deletion heterozyogsity was estimated as the average pairwise differences between D. simulans alleles as follows:

π_i_ is the coverage-weighted average expected heterozygosity of nucleotide variants (*i* = *nt*), deletions (*i* =Δ) or insertions (*i* = ▿) per base pair. “Expected heterozygosity” assumes the six sequenced genomes were drawn from a single, randomly mating population. Variable coverage over sites led us to extend the typical calculation of expected heterozygosity [127,128] to the following:

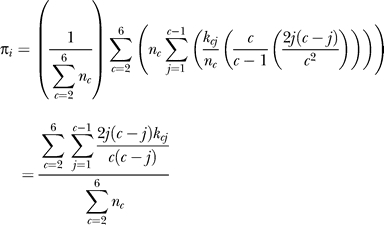
where *n_c_* is the number of aligned base pairs in the genomic region (e.g., gene feature or window) with sequencing coverage *c*. *k_cj_* is the number of sites in this region with coverage *c* at which the derived state (*nt*, ▵, or ▿) occurs in *j* out of the *c* sequences. This estimator was used for 10-kb windows, 50-kb windows, 30-kb sliding windows (10-kb increments), 150-kb sliding windows (10-kb increments), and 210-kb windows (10-kb increments), including all windows for which coverage was >200 bp. Expected heterozygosity was also estimated for genomic features (exons, introns, UTRs, and intergenic sequence) that had a minimum size and coverage [i.e., *n*(*n* – 1) × *s* ≥ 100, where *n* = average number of alleles sampled and *s* = number of sites]. For coding regions, the numbers of silent and replacement sites were counted using the method of Nei and Gojobori [129]. The pathway between two codons was calculated as the average number of silent and replacement changes from all possible paths between the pair.

The variance of pairwise differences in sliding windows (150-kb windows, 10-kb increments) was used as a method of summarizing the magnitude of linkage disequilibrium across the D. simulans genome. For each window, we calculated coverage weighted variance of the expected heterozygosity (see above) for all pairs of alleles.

*Divergence.* Unpolarized (i.e., pairwise) divergence between D. simulans and D. melanogaster was estimated for 10-kb windows, 50-kb windows, 30-kb sliding windows (10-kb increments), 150-kb sliding windows (10-kb increments), 210-kb windows (10-kb increments), and genomic feature that had a minimum number of nucleotides represented [i.e., *n* × *s* > 100, with *n* and *s* as above in calculations of π. Unpolarized divergence was calculated as the average pairwise divergence at each site, which was then summed over sites and divided by the total number of sites. A Jukes-Cantor [130] correction was applied to account for multiple hits. For coding regions, the numbers of silent and replacement sites were counted using the method of Nei and Gojobori [129]. The pathway between two codons was calculated as the average number of silent and replacement changes from all possible paths between the pair. Estimates of unpolarized divergence over chromosome arms were calculated for each feature with averages weighted by the number of sites per feature.

Lineage-specific divergence was estimated by maximum likelihood using PAML v3.14 [131] and was reported as a weighted average over each line with greater than 50 aligned sites in the segment being analyzed. Maximum likelihood estimates of divergence were calculated over 10-kb windows, 50-kb windows, 30-kb sliding windows (10-kb increments), 150-kb sliding windows (10-kb increments), 210-kb windows (10-kb increments), and gene features (exons, introns, and UTRs). PAML was run in batch mode using a BioPerl wrapper [132]. For noncoding regions and windows, we used baseml with HKY as the model of evolution to account for transition/transversion bias and unequal base frequencies [133]; for coding regions, we used codeml with codon frequencies estimated from the data.

Insertion and deletion divergence was calculated as *div_i_*, the coverage-weighted average divergence of deletions (*i* = ▵) or insertions (*i* = ▿) per base pair.

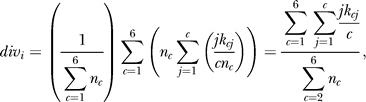
where *n_c_* is the number of aligned base pairs in the genomic region (e.g., gene feature or window) with sequencing coverage *c*. *k_cj_* is the number of sites in this region with coverage *c* at which the derived state with respect to the D. melanogaster reference sequence (▵ or ▿) occurs in *j* out of the *c* sequences.

### MK tests (unpolarized and polarized).

Unpolarized MK tests [4] used D. simulans polymorphism data and the D. melanogaster reference sequence for counting fixed differences. Polarized MK tests used D. yakuba to infer the D. simulans/D. melanogaster ancestral state. For both polarized and unpolarized analyses, we took the conservative approach of retaining for analysis only codons for which there were no more than two alternative states. For cases in which two alternative codons differed at more than one position, we used the pathway between codons that minimized the number of nonsynonymous substitutions. This is conservative with respect to the alternative hypothesis of adaptive evolution. Polymorphic codons at which one of the D. simulans codons was not identical to the D. melanogaster codon were not included. To reduce multiple testing problems, we filtered the data to retain for further analysis only genes that exceeded a minimum number of observations; we required that each row and column in the 2 × 2 table (two variant types and polymorphic versus fixed) sum to six or greater. Statistical significance of 2 × 2 contingency tables was determined by Fisher's Exact test. MK tests were also carried out for introns and Gold Collection UTRs by comparing synonymous variants in the associated genes with variants in these functional elements. For intergenic MK tests, we used synonymous variants from genes within 5 kb of the 5′ and/or 3′boundary of the intergenic segment. For some analyses, we restricted our attention to MK tests that rejected the null in the direction of adaptive evolution. This categorization was determined following Rand and Kann [134].

Polarized 2 × 2 contingency tables were used to calculate α, which under some circumstances can be thought of as an estimate of the proportion of variants fixing under selection [11]. Bootstrap confidence intervals of α and of the ratio of polymorphic-to-fixed variants for each functional element (Table 2) were estimated in R using bias correction and acceleration [135].

### Rate variation.

Our approach takes overall rate variation among lineages into account when generating expected numbers of substitutions under the null model and allows for different rates of evolution among chromosome arms (e.g., a faster-*X* effect). For example, the number of substitutions for all *X*-linked 50-kb windows was estimated using PAML (baseml), allowing different rates for each lineage. All D. simulans lines were used, with the estimated substitution D. simulans rate for each window being the coverage-weighted average. This generated an empirically determined branch length of the *X* chromosome for the average over each of the D. simulans lines (from all three way comparisons with D. melanogaster and D. yakuba) weighted by the number of bases covered. We carried out a relative rate test for windows or features in D. simulans and D. melanogaster by generating the expected number of substitutions for each window/feature/lineage based on the branch length of the entire chromosome in each lineage (PAML) and the coverage of the window/feature in question in each lineage. We then calculated the deviation from the expected number of substitutions as (observed – expected substitutions)^2^/expected substitutions for any window/feature/lineage.

### GO by MK permutations.

For each GO term associated with at least five MK tests, we calculated the proportion of significant (*p* < 0.05) tests. We then randomly selected *n p*-values from the set of all MK *p*-values, where *n* is the number of tests in the ontology category. We repeated this procedure 10,000 times to get the empirical distribution of the proportion significant *p*-values for each GO term.

### GO by *dN* permutations.

The relative rate χ^2^ for *dN* was calculated for each gene as described above. Genes showing a significant (*p* < 0.05) acceleration of *dN* in the D. simulans lineage were identified as described in the previous section. The probabilities of observing as many, or more, significant relative rate χ^2^ tests for *dN* were determined by permutation as described in the previous section.

### GO terms under “hitchhiking” windows.

We retrieved ontology terms associated with genes that fell under windows of interest in linked selection analyses. Then, for each term, we divided the number of instances that the term was represented in the windows of interest by the total number of genes in the genome that are associated with the ontology term. This gave us a proportional representation of each GO term in windows of interest. We compared this proportion for each GO term with the empirical distribution of proportions derived from permuted datasets. For each permuted dataset, we randomly picked a nonoverlapping set of windows that were the same size in numbers of base pairs as the observed windows. Each window was guaranteed to contain at least one gene, given that windows of interest have higher-than-average gene density. We then retrieved the ontology terms associated with the genes under the random set of windows. We next calculated the proportion of each term as described above for the observed windows. We repeated this procedure 1,000 times to obtain an empirical distribution of proportions of each term in random windows. The proportion of each GO term in the original observed windows of interest was compared to this empirical distribution to obtain a probability of observing that proportion of each term in windows of interest.

### GO clustering.

We wanted to know whether ontology terms were clustered in the genome. We tested whether each ontology term was significantly clustered by calculating the coefficient of variation based on occurrence in 1-Mb, nonoverlapping windows and compared that to the coefficient of variation from permuted datasets in which we randomized the locations of genes on each chromosome arm.

### Gene expression.

Genes were assigned to expression categories, with the goal of determining whether certain categories had a greater proportion of significant MK tests for adaptive protein divergence than expected by chance. Two types of data, expressed sequence tag (EST) collections and microarray experiments, were used. Genes associated with EST collections from D. melanogaster (head, ovary, and testis from Flybase and spermatheca from Swanson et al [136]) were assigned to that tissue expression category. Female-mating responsive genes were those defined by microarray experiments [137]. Male- and female-biased genes were defined based on microarray experiments of Parisi et al. [138] and Arbeitman et al. [139]. Male- and female-biased genes from Parisi et al. [138] were obtained directly from their Tables S41 and S42. Arbeitman et al. [139] measured expression over the D. melanogaster life cycle for adult males and females. We averaged expression for each gene over the time points taken for each stage. For example, there were 30 time point measurements during the egg stage; we used the average expression over those 30 time points. We repeated this for larvae, metamorph, adult female, and adult male stages. Each gene was provisionally designated as having biased expression for the stage with the maximum average expression, which we will call the biased stage. For each gene, we calculated the average difference between the biased stage expression value and the other stage expression values. This generated a distribution of differences for each comparison of stages. A gene was finally determined to have biased expression if the average difference between the biased stage and the other stages fell into top half for that stage distribution. This procedure resulted in 592, 374, 223, 466, and 296 stage-biased genes for egg, larvae, metamorph, adult male, and adult female stages, respectively. We calculated the proportion of genes in a group (e.g., male-biased) that had significant MK tests (*p* < 0.05). We used permutation testing to determine whether the proportion of significant polarized MK tests deviating in the direction of adaptive protein evolution exceeded the 95% tail of the empirical distribution, based on 10,000 datasets of randomly selected MK tests, sampled without replacement.

### Protein–protein interactions.

We tested whether pairs of proteins that interact with one another were more likely to show evidence of adaptive protein divergence than random pairs of proteins with no evidence of interaction. Data were from Giot et al. [140]. We considered pairs of genes to have a significant interaction if the probability of interaction was greater than 0.5. We calculated the proportion of interacting pairs where both members had significant evidence of adaptive evolution (MK *p*-values < 0.05). We compared this proportion to the distribution of proportion generated from permuted datasets generated by randomly drawing pairs of genes without replacement from the Giot et al. [140] dataset.

### Polymorphism versus divergence.

Hudson, Kreitman, and Aquadé [2] proposed a test of the neutral theory of molecular evolution in which the numbers of polymorphic and (fixed) divergent sites are contrasted between two independent loci (genomic regions). The distribution of a χ^2^-like test statistic can be determined by simulation for any assumed values of recombination within each locus. However, given the small sample size here and the genomic scale of the data, we used an analogous statistic for polymorphisms and fixations on the D. simulans lineage in various sizes of sliding windows, combined over coverage classes. First, the average proportion of segregating sites in D. simulans and parsimony-inferred fixed differences for each chromosome arm in D. simulans was determined for each coverage class over a range of sliding window sizes (10 kbp to 510 kbp). The test statistic is a simple two-cell χ^2^, in which the observed numbers (summed over coverage classes) of segregating and fixed sites are contrasted with their expected numbers (summed over coverage classes, the chromosome arm average for each coverage class times the total numbers of segregating and fixed sites in that class). Only sites for which unambiguous, parsimony-inferred D. simulans/D. melanogaster ancestral states could be determined were included in the analysis. In a number of figures, χ[–log_10_(*p*)] is plotted; –log_10_ of *p*, critical value for this χ^2^, was given the sign of the difference, *observed numbers of segregating site – expect number of segregating sites*. As expected (Figure 1), there is a clear tendency for the level of polymorphism (both π*_nt_* and proportion of segregating sites) to decline proximal to the telomeres and centromeres. Therefore, the test statistics discussed in this section were determined by generating expected values as described above, but only including the “central euchromatic” regions. These were defined as the regions bounded by the first and last position on each chromosomes arm for which the proportion of segregating sites was greater than or equal to the chromosome arm average in a 510-kbp window. While this makes deviations in the centromere and telomere proximal regions appear greater, it removes the obvious bias toward positive deviations (i.e., excess polymorphism) that would be created by including large genomic regions known to show reduced polymorphism when generating expectations. Minimum values for the expected numbers of segregating and fixed sites were one (unless otherwise indicated). Windows with coverage <200 bp were dropped (unless otherwise indicated).

### Autocorrelation of nucleotide heterozygosity and divergence.

Expected nucleotide heterozygosity and polarized divergence were calculated for 10-kb and 50-kb nonoverlapping windows spanning each chromosome arm as described above. For each arm, autocorrelation between successive windows was calculated as:

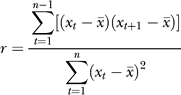
where there are *n* windows along an arm, and *x_t_* represents the value of nucleotide heterozyogsity or divergence for the *t-*th window. Significance of *r* for all arms for both polymorphism and divergence was calculated by permutation. All calculations were carried out in R (http://www.r-project.org).

### Reduced variation associated with colonization.

We set out to find putative selective sweeps that occurred concomitantly with migration by D. simulans out of Africa/Madagascar*.* We expect the signature of these sweeps to be low variation in New World (NW) lines, defined here as *w^501^* and *SIM4/6*, compared to Old World (OW) lines, defined here as *C167.4*, *MD199S*, and *MD106TS*. The method described here addresses the issue of autocorrelated loci. Our approach was to simulate datasets with the same degree of autocorrelation as that of the observed data, and to determine whether there are longer runs of windows with disproportionately low NW π in the actual data than one would expect by chance. All data manipulation, calculations, and simulations were carried out in R using functions available within the “base” and “stats” packages. Mean nucleotide diversity (π) from 10-kb nonoverlapping windows throughout the genome from the two NW and three OW lines were used. Adjacent 10-kb windows were averaged (weighted by coverage) to obtain 20-kb windows. Remaining windows for which no estimate of π was available were conservatively estimated by interpolation. There were no gaps in the 20-kb window data longer than three consecutive windows in either population. For each window, the ratio of NW π:OW π (π_ NW_:π_OW_) was measured. Maximum likelihood estimates of first-order coefficients of autocorrelation for each of the chromosome arms were found (all were significant).

Monte Carlo simulations of the ratio π_NW_:π_OW_ were performed according to the following procedure. We first randomly sampled ratios of π_ NW_: π_OW_ from the data with replacement for each arm separately; this ensures that our simulated data has the same mean and variance as the actual data. A first-order autoregressive filter was then applied to the randomly sampled data using the estimate of autocorrelation for the given chromosomal arm, according to the following relationship:

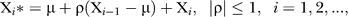
where parameter μ is the mean of the sampled data, ρ is the autocorrelation, X*_i –_*_ 1_ is previous value in the series, and X*_i_* is the original sampled measure for the *i*th window. This filter imposes the observed autocorrelation onto the sampled data to mimic the observed autocorrelation, resulting in a new value, X_i_*, for each window. Variance and estimated first-order autocorrelation of the simulations were similar to those of the empirical data without altering this procedure.

A lower threshold of π_ NW_: π_OW_, below which 5% of the empirical data points reside, was determined. Significance of runs of windows below this threshold was determined by comparison to the distribution of the run lengths in 10,000 Monte Carlo simulation runs for each chromosome arm, performed as described above. *P*-values for each arm were corrected for multiple comparisons conservatively via Bonferroni correction (Dunn-Sidak corrections did not result in an increased number of significant sweeps).

### Preferred/unpreferred codons and base composition analyses.

 Parsimony was used to infer D. simulans/D. melanogaster ancestral states; D. yakuba was the outgroup. Only codons with one synonymous variant among the three species were included in these analyses. The preferred codon set was defined following Akashi [113]. For some analyses, preferred and unpreferred substitution rates were determined by dividing the number of substitutions of each type by the number of ancestral codons of the appropriate ancestral state (unpreferred ancestors for the preferred substitution rate and preferred ancestors for the unpreferred substitution rate), all inferred by parsimony. In principle, excess unpreferred polymorphisms at synonymous sites could erroneously lead one to infer directional selection on other sites. However, the ratio of preferred-to-unpreferred polymorphisms is not significantly different (pooled across genes or gene-by-gene) for UTRs that had significant versus nonsignificant MK tests in contrasts of synonymous and UTR sites. For introns that showed a significant MK test versus synonymous sites, there was a slightly larger ratio of unpreferred-to-preferred polymorphisms compared to the ratio for introns that were not significant. However, this was seen only in the pooled analysis and not in the gene-by-gene analysis. We found that significant intron and UTR MK tests had more similar coverages (e.g., 5′ UTR versus synonymous) compared to tests that were not significant, suggesting that the large number of significant noncoding versus synonymous tests cannot be explained by relatively small coverage differences across site-types. Overall, these data suggest that most of the highly significant MK tests of noncoding DNA are not explained by excess unpreferred polymorphisms or coverage variation.

Base composition analyses on noncoding DNA were carried out in a similar fashion, with parsimony being used to infer the D. simulans/D. melanogaster ancestor. Only unambiguous parsimony-inferred sites were used in these analyses.

### Estimates of cM/kb across the *X* chromosome.

All *X*-linked genes for which Flybase reported genetic and physical locations (first nucleotide of the gene in Flybase annotation of D. melanogaster v4.2) were used. Genetic and physical distances were determined for 12-gene intervals, sliding one gene at a time; estimates of cM/kb per interval were used as estimates of recombination rate per physical length. Mean physical and genetic distances per interval were 1.55 Mb and 5 cM, respectively. Two intervals with negative estimates of cM/kb, indicative of discordant genetic and physical data were removed, leaving estimates of cM/kb for 150 intervals. The physical location of the interval was defined as the midpoint between physical locations of the first and last gene. For analyses investigating correlations of 50-kb windows of polymorphism and divergence with crossing-over, midpoints were rounded to the nearest 50,000. If multiple intervals were rounded to the same number, the distal interval was used in the analyses.

### Transposable elements.

Cloned elements. The “hanging ends” of well-mapped plasmid clones that were not fully aligned to D. melanogaster were examined by BLAST for extensive (100 bp or greater), high-quality (90% or greater) sequence similarity to known transposable elements of D. melanogaster (v 9.2, http://www.fruitfly.org/p_disrupt/TE.html). The coordinates are slightly rounded to facilitate finding duplicates slightly off in alignment.

*Clustered elements.* This analysis used plasmid clones for which only one mate pair mapped uniquely and unambiguously to the genome according to the method described previously. The other mate pair was compared to the D. melanogaster transposable element database v9.2. If the read mapped uniquely and unambiguously to a transposable element (90% coverage, 90% identity, at least two high quality differences to a secondary candidate), a transposable element was considered as mapped to the general genomic location of its mate pair. The estimated location begins at the end of the mate pair read and ends 10 kb away in the appropriate direction determined by the direction of the alignment. Transposable elements from the same family located within 5 kb of each other in the same D. simulans line were considered the same element, and therefore, were clustered.

## Supporting Information

Dataset S1 Estimates of Polymorphism, Divergence, and Counts of Polymorphic and Fixed Sites for CDS (2.1 MB TXT)

Dataset S2 Estimates of Polymorphism, Divergence, and Counts of Polymorphic and Fixed Sites for Introns (956 KB TXT)

Dataset S3 Estimates of Polymorphism, Divergence, and Counts of Polymorphic and Fixed Sites for Gold Collection 5′ UTRs (346 KB TXT)

Dataset S4 Estimates of Polymorphism, Divergence, and Counts of Polymorphic and Fixed Sites for Gold Collection 3′ UTRs (396 KB TXT)

Dataset S5 Estimates of Polymorphism, Divergence, and Counts of Polymorphic and Fixed Sites for Intergenic Regions (1.7 MB TXT)

Dataset S6 Estimates of Polymorphism, Divergence, and Counts of Polymorphic and Fixed Sites for CDS in Heterochromatic Regions (53 KB TXT)

Dataset S7 Estimates of Polymorphism and Divergence for 10-kb Windows. Coordinates reflect D. melanogaster genomic organization.
(855 KB TXT)

Dataset S8 Estimates of Polymorphism and Divergence for 50-kb Windows. Coordinates reflect D. melanogaster genomic organization.
(177 KB TXT)

Dataset S9 Frequencies of Synonymous and Nonsynonymous Variants and Base Composition Variants for Coverage Classes Three–Six P and U are preferred and unpreferred, respectively (e.g., up = unpreferred-to-preferred).
(60 KB XLS)

Dataset S10 Counts of Polymorphic and Fixed Variants of Preferred and Unpreferred Codons (133 KB TXT)

Dataset S11 *X* Chromosome Insertion and Deletion Polymorphism and Divergence Estimates for 150-kb Sliding Windows (Sliding by 10 kb) (108 KB TXT)

Dataset S12 *2L* Chromosome Insertion and Deletion Polymorphism and Divergence Estimates for 150-kb Sliding Windows (Sliding by 10 kb) (116 KB TXT)

Dataset S13 *2R* Chromosome Insertion and Deletion Polymorphism and Divergence Estimates for 150-kb Sliding Windows (Sliding by 10 kb) (105 KB TXT)

Dataset S14 *3L* Chromosome Insertion and Deletion Polymorphism and Divergence Estimates for 150-kb Sliding Windows (Sliding by 10 kb) (123 KB TXT)

Dataset S15 *3R* Chromosome Insertion and Deletion Polymorphism and Divergence Estimates for 150-kb Sliding Windows (Sliding by 10 kb) Coordinates reflect D. simulans genomic organization by accounting for the inversion fixed on *3R* in D. melanogaster.
(150 KB TXT)

Dataset S16 *X* Chromosome Nucleotide Polymorphism and Divergence Estimates and HKA test statistics for 10-kb Windows (87 KB TXT)

Dataset S17 *2L* Chromosome Nucleotide Polymorphism and Divergence Estimates and HKA test statistics for 10-kb Windows (93 KB TXT)

Dataset S18 *2R* Chromosome Nucleotide Polymorphism and Divergence Estimates and HKA test statistics for 10-kb Windows (86 KB TXT)

Dataset S19 *3L* Chromosome Nucleotide Polymorphism and Divergence Estimates and HKA test statistics for 10-kb Windows (100 KB TXT)

Dataset S20 *3R* Chromosome Nucleotide Polymorphism and Divergence Estimates and HKA test statistics for 10-kb Windows. Coordinates reflect D. simulans genomic organization by accounting for the inversion fixed on *3R* in D. melanogaster.
(122 KB TXT)

Figure S1 Patterns of Polymorphism and Divergence of Small Deletions along the Chromosome Arms π for small deletions (blue) and the divergence from D. melanogaster (red) in 150-kbp windows are plotted every 10 kbp. χ[–log(*p*)] (olive) as a measure of the deviation (+/-) in the proportion of polymorphic deletions in 30-kbp windows is plotted every 10 kbp; see Materials and Methods.
(586 KB PDF)

Figure S2 Patterns of Polymorphism and Divergence of Small Insertions along the Chromosome Arms π, average number of insertions per bp (blue) and the pairwise divergence from D. melanogaster per bp (red) in 150 kbp windows are plotted every 10kbp. χ[–log(*p*)] (olive) as a measure of the deviation (+/-) in the proportion of polymorphic insertions in 30-kb windows is plotted every 10 kbp; see Materials and Methods.
(582 KB PDF)

Figure S3 Patterns of the Relative Rate Test, Nucleotide Divergence, and Deviation of Proportion of Divergence Nucleotide Sites The χ^2^ (red) for the relative rate test in 150-kbp windows is plotted every 10 kbp. *CV*(*π*) (orange), the coefficient of variation of nucleotide π in 150-kbp windows, is plotted every 10 kbp. χ[–log(*p*)] (olive) as a measure of deviation (+/-) in the proportion of sites in a 150-kbp windows is plotted every 10 kbp.
(559 KB PDF)

Figure S4 Patterns of TEs Insertions, Nucleotide Divergence, and GC Content along Chromosome Arms Distribution of total numbers of “clustered transposable elements” (TEs) in nonoverlapping 210-kbp windows (olive) along each of the arms of D. simulans (pooled across lines). The dashed (olive) lines are the regression lines of TEs numbers on position (bp), with the outliers (orange) masked from the data. Note the gapped scales for total TEs on the right. Average divergence on the D. simulans lineage (red) in 150-kbp windows are plotted every 10 kbp for reference along with the dashed regression line. GC content in D. simulans (blue) in 150-kbp windows are plotted every 10 kbp for reference along with the dashed regression line.
(553 KB PDF)

Figure S5 Copy Numbers of TE Families in D. simulans and *D. melanogaster*
The numbers of each TE family in the D. melanogaster reference sequence is plotted against the numbers identified in the D. simulans genomes (see Materials and Methods). The lower-left panel is an enlargement of the lower ranges.
Red, Long Terminal Repeat (LTR) containing retrotransposons; blue, non-LTR retrotransposons; orange, foldback elements; olive, inverted repeat elements; and black, MITE and SINE-like.
(38 KB PDF)

Table S1 Coding and Noncoding Nucleotide Heterozygosity in D. simulans; Lineage-Specific Nucleotide Divergence in D. simulans, D. melanogaster, and D. yakuba; and Pairwise Nucleotide Divergence for *D. simulans-D. melanogaster*
UTRs are from the Gold Collection genes.
(142 KB DOC)

Table S2 Nonsynonymous (NS) and Synonymous (S) Variants in Heterochromatic versus Euchromatic Genes (38 KB DOC)

Table S3 Comparisons of D. simulans versus D. melanogaster Divergence and *X* versus Autosome Divergence for D. simulans, D. melanogaster, and D. yakuba
(58 KB DOC)

Table S4 Comparison of *X* and Autosome Polarized Polymorphic Variants in Different Frequency Classes for Sites with Coverage *n* = 5 or *n* = 6 D. simulans Alleles (51 KB DOC)

Table S5 Spearman Correlations of Nucleotide Heterozygosity, Nucleotide Divergence, Relative Rate χ^2^ Tests, Ancestral GC Content, and D. simulans Transposable Element Density (all Measured in 50-kb Windows) versus Proximal–Distal Location along Chromosome Arms Positive correlations for *2L*, *3L*, and *X*, and negative correlations for *2R* and *3R* indicate increasing values closer to centromeres. Inv*3R* was used for D. simulans lineage inferences. Trimmed data indicates analyses for which regions of low heterozygosity were removed (Materials and Methods).
(70 KB DOC)

Table S6 Autocorrelations of D. simulans Nucleotide Polymorphism and Divergence (10- and 50-kb Windows) along Chromosome Arms All are significant at *p* < 0.0001.
(52 KB DOC)

Table S7 GO Terms Overrepresented among Genes in HKA Windows Having Unusually Low Ratios of Nucleotide Heterozygosity to Divergence CC, MF, and BP are cellular component, molecular function, and biological process, respectively.
(113 KB DOC)

Table S8 Mean (SE) Ratio of Nucleotide Heterozygosity (50-kb Windows, Weighted by Coverage) for New World versus Old World Lines (27 KB DOC)

Table S9 Regions of the Genome Showing Disproportionate Reductions of Nucleotide Heterozygosity in the US Sample (29 KB DOC)

Table S10 >Genes Located in Genomics Regions Showing Disproportionate Reductions of Nucleotide Heterozygosity in the US Sample (68 KB DOC)

Table S11 GO Terms Overrepresented in Windows from Out-of-Africa/Madagascar Analysis. MF and BP, molecular function and biological process, respectively
(50 KB DOC)

Table S12 GO Terms Associated with the Top 20 Genes with the Smallest Unpolarized MK Test *p*-Value (118 KB DOC)

Table S13 Genes Showing Excess Protein Polymorphism (*p* < 0.01) in Polarized MK Tests (65 KB DOC)

Table S14 GO Terms Associated with the Top 20 Genes with the Smallest Polarized MK Test *p*-Values (111 KB DOC)

Table S15 GO Categories Enriched among Genes with Significant (*p* < 0.05) Unpolarized MK Tests (74 KB DOC)

Table S16 GO Categories Enriched among Genes with Significant (*p* < 0.05) Polarized MK Tests (145 KB DOC)

Table S17 Tissue-Specific or Developmental Stage–Specific Expression Patterns Enriched with Significant (*p* < 0.05) MK Tests (53 KB DOC)

Table S18 Genes Having the Greatest Relative Rate Test χ^2^ Statistics for *dN* in the D. simulans Lineage (68 KB DOC)

Table S19 Genes Having the Greatest Relative Rate Test χ^2^ Statistics for *dN* in the D. melanogaster Lineage (63 KB DOC)

Table S20 GO Categories Enriched among Proteins Showing Accelerated Protein Evolution (χ Test *p*-Value < 0.01) in the D. simulans Lineage (215 KB DOC)

Table S21 GO Categories Enriched among Proteins Showing Accelerated Protein Evolution (χ^2^ Test *p*-Value < 0.01) in the D. melanogaster Lineage (205 KB DOC)

Table S22 Genes Associated with the Most-Significant 5′ UTR Polarized MK Tests (Average Coverage per Site > 2) (55 KB DOC)

Table S23 Genes Associated with the Most-Significant 3′ UTR Polarized MK Tests (Average Coverage per Site > 2) (52 KB DOC)

Table S24 Genes Associated with the Most-Significant Intron MK Tests (Average Coverage per Site > 2) (64 KB DOC)

Table S25 Number (Frequency) of Nonsynonymous and Noncoding Polymorphisms (Sites with Coverage of *n* = 5 or *n* = 6 D. simulans Alleles) for Different Frequency Classes (40 KB DOC)

Table S26 Counts and Substitution Rates per Site of Preferred and Unpreferred Variants “Fixed” along the D. simulans and D. melanogaster Lineages (Inferred by Parsimony) Substitution rates were determined by dividing the number of preferred/unpreferred fixations by the number of unpreferred/preferred ancestral bases.
(74 KB DOC)

Table S27 *X* and A, Polymorphic and Fixed, Preferred and Unpreferred Variants for Sites with Coverages Four, Five, or Six (33 KB DOC)

Table S28 Unpreferred Polymorphisms (Coverage Five Sites) Occur at Lower Frequency than Preferred Polymorphisms (30 KB DOC)

Table S29 Genes with Significant Polarized MK Tests Have a Higher Proportion of Preferred Fixations than Genes with Nonsignificant MK Tests (27 KB DOC)

Table S30 Preferred, Unpreferred, and Noncoding GC/AT Fixed Variants across the Genome (Coverage Classes Three–Six) (27 KB DOC)

Table S31 Polymorphic GC Variants Occur at Higher Frequency than Polymorphic AT Variants *X*-linked polymorphic GC variants occur at higher frequency than autosomal polymorphic GC variants (coverage-six polymorphisms from intergenic and intron DNA).
(32 KB DOC)

Table S32 D. yakuba Genome Input and Assembly Statistics Statistics presented are for the whole-genome assembly before it was anchored using alignments to D. melanogaster. “Contigs” are contiguous sequences not interrupted by gaps, and “supercontigs” are ordered and oriented “contigs” including estimated gap sizes. The N50 statistic is defined as the largest length L such that 50% of all nucleotides are contained in contigs of size at least L. The total contig size was 167 Mb, with 97% of the consensus base pairs having quality scores of at least 40 (Q40) (expected error rate of less than or equal to 10^−4^) and 98% are at least Q20.
(59 KB DOC)

Table S33 Read and Trim Statistics for D. simulans Syntenic Assemblies (35 KB DOC)

Table S34 Correlation (Kendall's τ) between Copy Numbers of TE Families in “Trimmed” Euchromatic Regions of D. simulans and D. melanogaster
The *simulans* TEs are the “clustered” TEs. The *melanogaster* TEs are those annotated in release 4.0.
(31 KB DOC)

Table S35 Tests of the Homogeneity of the Proportions of Each Family across Six D. simulans Lines, Homogeneity of Classes across Lines, and Homogeneity of Families within Classes across Lines (33 KB DOC)

Table S36 Test of the Homogeneity of Relative Family Copy Numbers across the Five Chromosome Arms (Pooled across Lines) for All TEs and within the Four Classes (33 KB DOC)

Table S37 Test of the Homogeneity of Relative Family Copy Numbers on the *X* chromosome versus the Autosomes (Pooled across Lines) for All TEs and within the Four Classes (32 KB DOC)

Table S38 Heterogeneity of “Cloned” TE Numbers in Various Gene Annotation Elements (29 KB DOC)

Table S39 Comparison of Expected D. simulans Nucleotide Heterozygosity and Divergence for 30-kb Windows Centered on the Estimated Position of “Clustered” TEs (+) Compared to Windows without Clustered TEs (–) The difference between the distributions (TEs: +/-) was tested with the Mann-Whitney *U* test; the *p*-value is in the upper position in the last column (probability < / ratio). The ratio of the means is also shown (lower in last column).
(50 KB DOC)

Text S1 Transposable Elements (48 KB DOC)

### Accession Numbers

The GenBank (http://www.ncbi.nlm.nih.gov/Genbank/) accession number for *D. yakuba* is AAEU01000000 (version 1) and for the *D. simulans w^501^* whole-genome shotgun assembly is TBS-AAEU01000000 (version 1).

## Abbreviations

CDS: coding sequence
GO: gene ontology
indel: insertion/deletion
MK test: McDonald and Kreitman test
UTR: untranslated region
