# Supplementary material for: Population Genomics: Whole-Genome Analysis of Polymorphism and Divergence in Drosophila simulans
Source: PLoS Biol. 2007 Nov 6;5(11):e310. doi: 10.1371/journal.pbio.0050310 (PMC2062478; doi:10.1371/journal.pbio.0050310)
Supplement: Supplementary file 1 [file pbio.0050310.sg001.pdf]

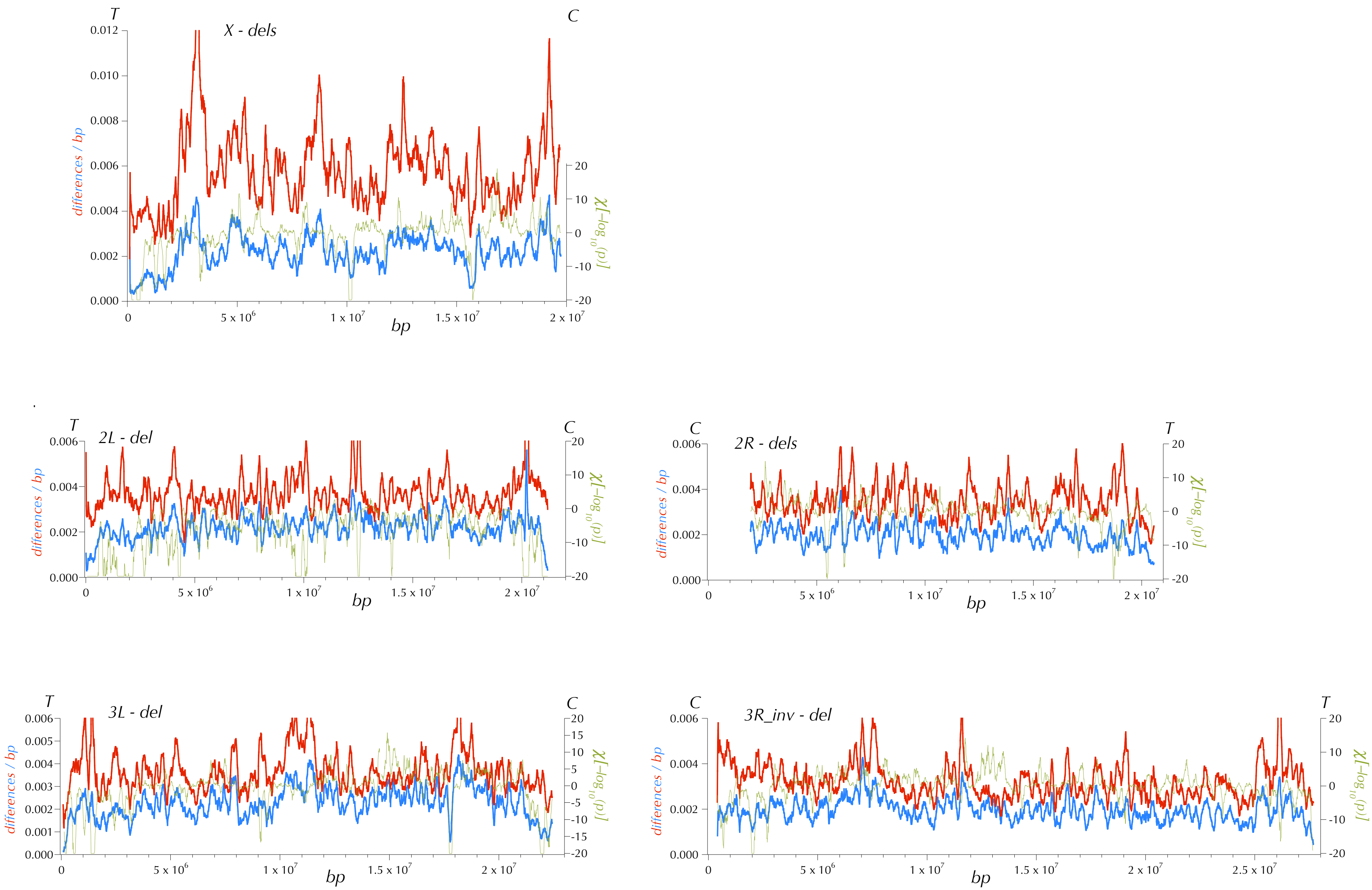

X - dels
2L - del
2R - dels
3L - del
3R_inv - del
T
C
differences / bp
χ[−log10 (p)]
bp
0
5 x 10^6
1 x 10^7
1.5 x 10^7
2 x 10^7
2.5 x 10^7
