# Supplementary material for: Population Genomics: Whole-Genome Analysis of Polymorphism and Divergence in Drosophila simulans
Source: PLoS Biol. 2007 Nov 6;5(11):e310. doi: 10.1371/journal.pbio.0050310 (PMC2062478; doi:10.1371/journal.pbio.0050310)
Supplement: Supplementary file 2 [file pbio.0050310.sg002.pdf]

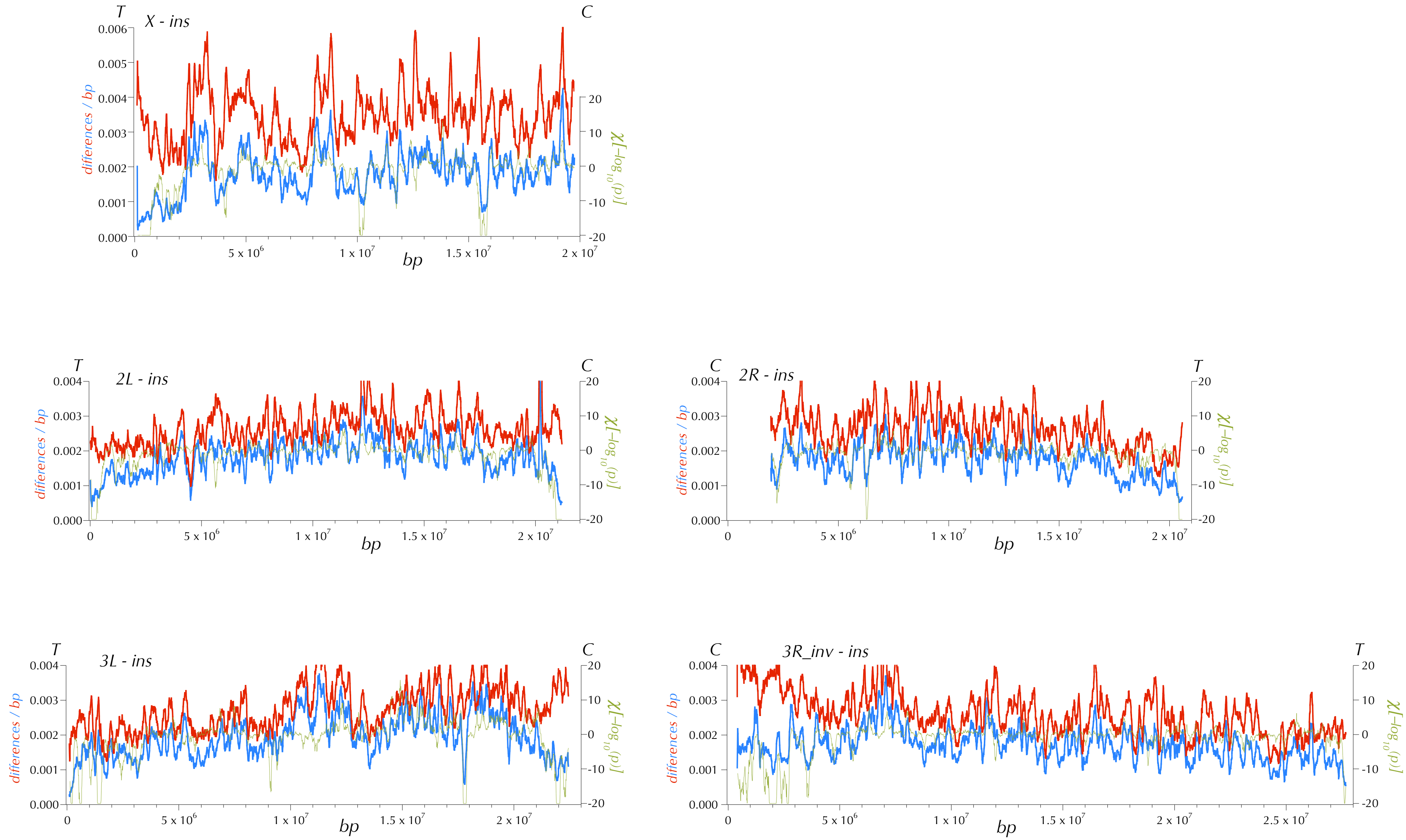
X - ins
2L - ins
2R - ins
3L - ins
3R_inv - ins
T
C
differences / bp
$\chi[-\log_{10}(p)]$
bp
0.000
0.001
0.002
0.003
0.004
0.005
0.006
-20
-10
0
10
20
0
5 x 10^6
1 x 10^7
1.5 x 10^7
2 x 10^7
2.5 x 10^7
