# Supplementary material for: Population Genomics: Whole-Genome Analysis of Polymorphism and Divergence in Drosophila simulans
Source: PLoS Biol. 2007 Nov 6;5(11):e310. doi: 10.1371/journal.pbio.0050310 (PMC2062478; doi:10.1371/journal.pbio.0050310)
Supplement: Supplementary file 3 [file pbio.0050310.sg003.pdf]

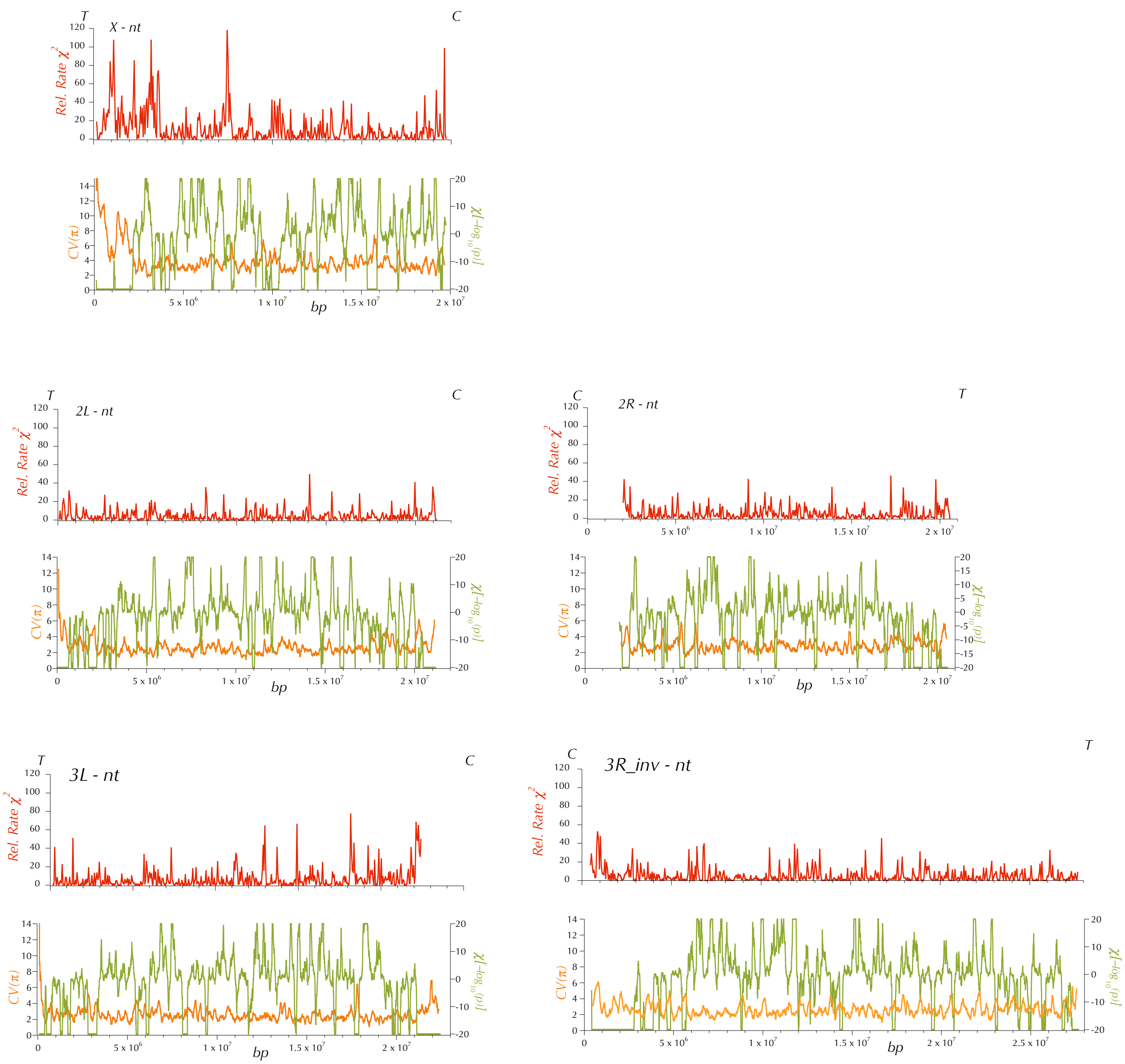
T
X - nt
C
Rel. Rate χ2
CV(π)
χ[-log10(p)]
bp
0
5 x 10^6
1 x 10^7
1.5 x 10^7
2 x 10^7
T
2L - nt
C
C
2R - nt
T
T
3L - nt
C
C
3R_inv - nt
T
2.5 x 10^7
