# Supplementary material for: Population Genomics: Whole-Genome Analysis of Polymorphism and Divergence in Drosophila simulans
Source: PLoS Biol. 2007 Nov 6;5(11):e310. doi: 10.1371/journal.pbio.0050310 (PMC2062478; doi:10.1371/journal.pbio.0050310)
Supplement: Supplementary file 4 [file pbio.0050310.sg004.pdf]

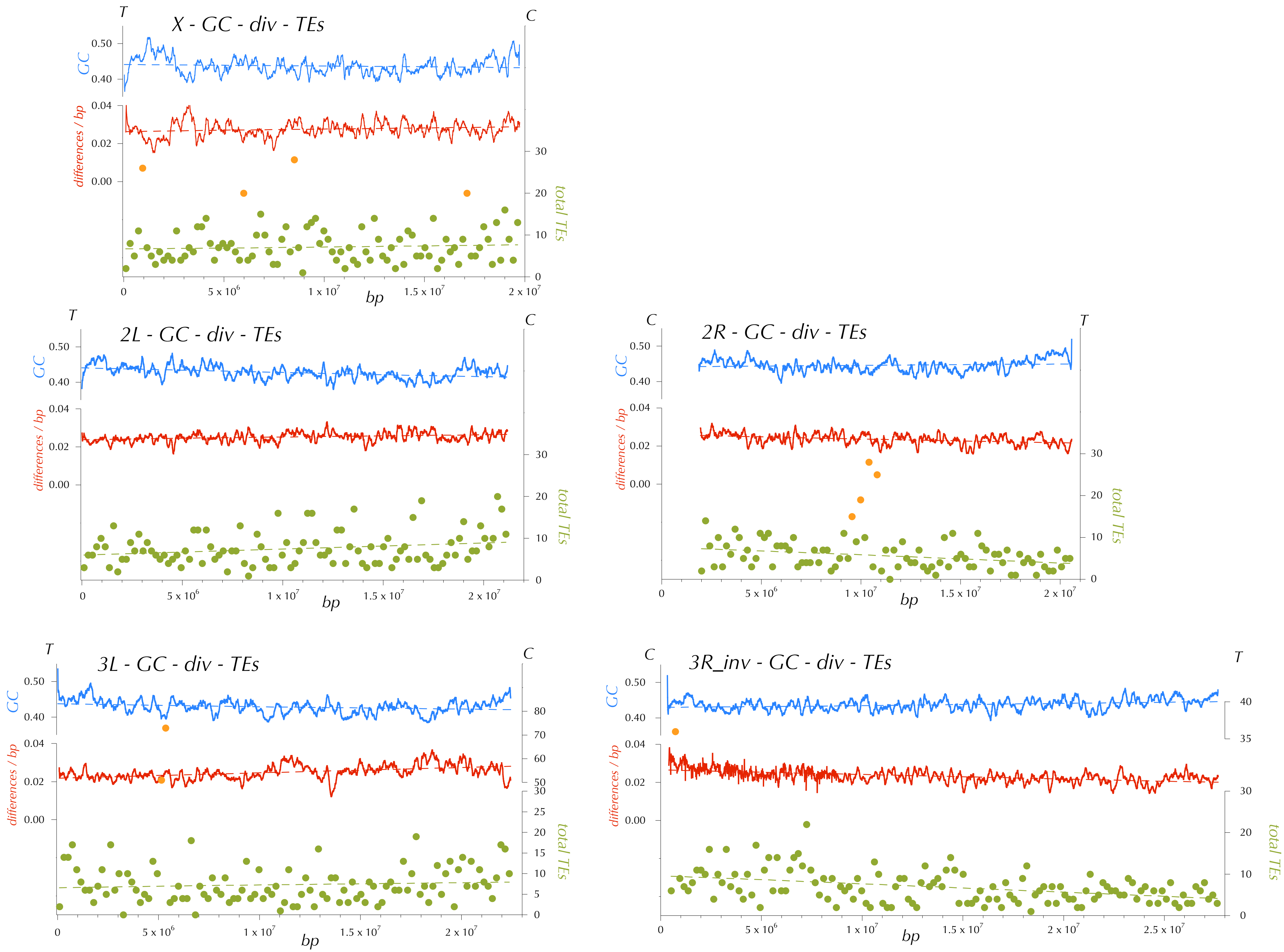
X - GC - div - TEs
T
C
GC
0.50
0.40
differences / bp
0.04
0.02
0.00
total TEs
30
20
10
0
0
5 x 10^6
1 x 10^7
1.5 x 10^7
2 x 10^7
bp
2L - GC - div - TEs
2R - GC - div - TEs
3L - GC - div - TEs
80
70
60
50
25
15
5
3R_inv - GC - div - TEs
40
35
2.5 x 10^7
