# Supplementary material for: Population Genomics: Whole-Genome Analysis of Polymorphism and Divergence in Drosophila simulans
Source: PLoS Biol. 2007 Nov 6;5(11):e310. doi: 10.1371/journal.pbio.0050310 (PMC2062478; doi:10.1371/journal.pbio.0050310)
Supplement: Supplementary file 5 [file pbio.0050310.sg005.pdf]

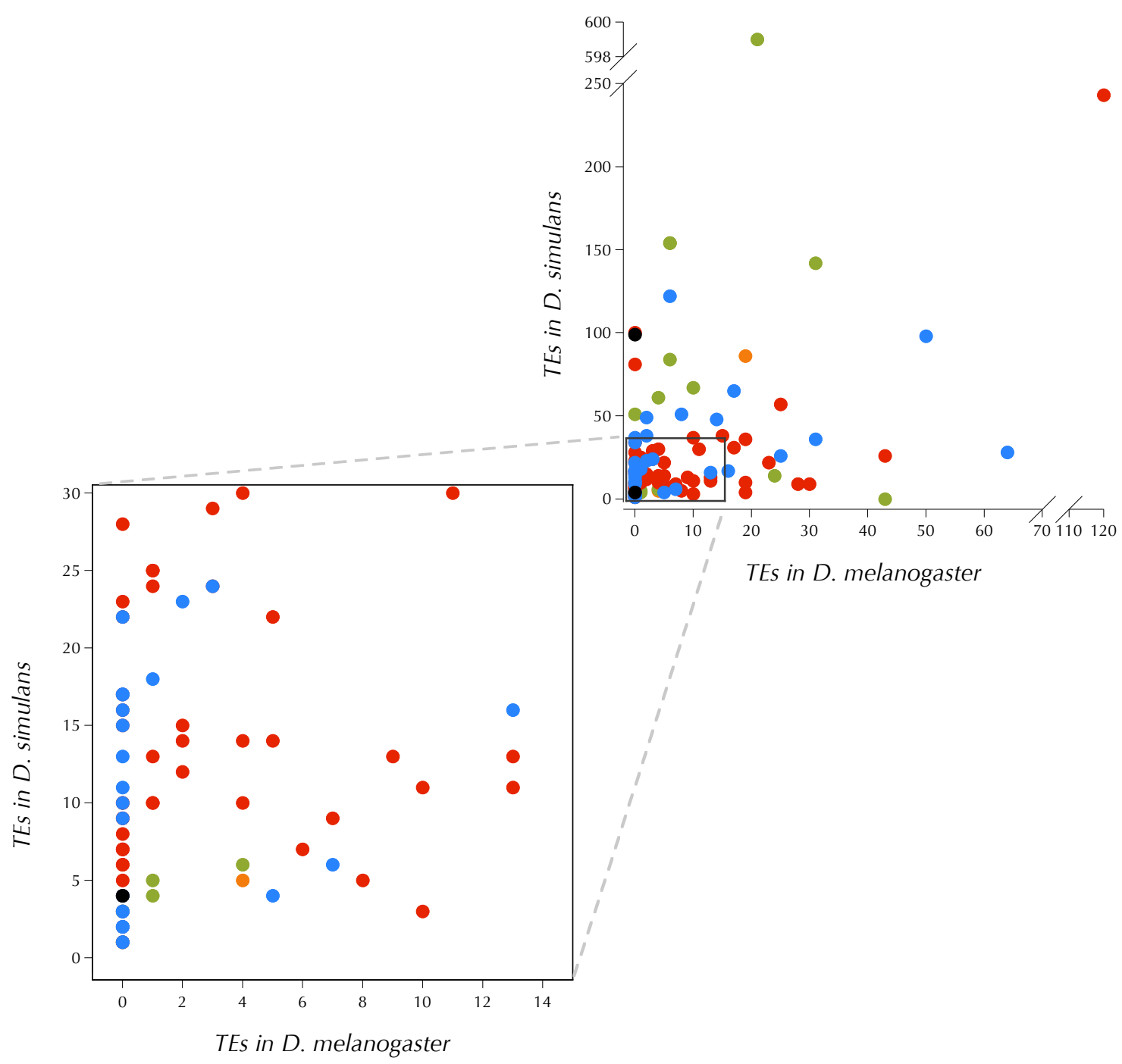

600
598
250
200
150
100
50
0
TEs in D. simulans
0
10
20
30
40
50
60
70
110
120
TEs in D. melanogaster
30
25
20
15
10
5
0
TEs in D. simulans
0
2
4
6
8
10
12
14
TEs in D. melanogaster
