# Supplementary material for: Population Genomics: Whole-Genome Analysis of Polymorphism and Divergence in Drosophila simulans
Source: PLoS Biol. 2007 Nov 6;5(11):e310. doi: 10.1371/journal.pbio.0050310 (PMC2062478; doi:10.1371/journal.pbio.0050310)
Supplement: Supplementary file 6 [file pbio.0050310.sd021.doc]

# Transposable Elements

**General patterns between species**: *D. simulans* transposable elements (TEs) that show high sequence similarity to annotated *D. melanogaster* TEs were physically mapped at two levels of precision. First, “clustered” TEs were placed for mate-pairs in which one read mapped uniquely and the other mapped to a known *D. melanogaster* TE. The precise location of these 3857 euchromatic insertions is ambiguous (Materials and Methods). Second, 434 “cloned” euchromatic TEs were precisely mapped when a long, high-quality read contained a large segment of uniquely mapped sequence that was contiguous with a large segment of high similarity to a known *D. melanogaster* TE. *D. melanogaster* TEs (*n* = 1572) have been organized [1] and recently updated to include 129 families (based on sequence similarity; Materials and Methods) and four classes - long terminal repeat (*LTR*), non-*LTR*, inverted repeat (*IR*) and foldback (*FB*). We found 126 of these families (941 TEs) in the euchromatic regions homologous to the “trimmed euchromatic regions” in *D. simulans* (see Materials and Methods); however, only 68 TEs were reported for the homologous euchromatic regions in the *D. melanogaster* reference sequence. One TE, a *pogo* element, was found in the *D. melanogaster* euchromatic reference sequence but not in the corresponding *D. simulans* data.

Figure S5 and Table S34 reveal that copy number of clustered TEs for individual families are correlated in *D. simulans* vs. *D. melanogaster* (Kendall’s *τ* = 0.36). Nevertheless, some families that were found only in more heterochromatic (repeat rich) centromere- and telomere-proximal regions in *D. melanogaster* are found in the euchromatic regions of one or more *D. simulans* lines. Furthermore, a few of the most common *D. melanogaster* TEs are considerably less common in *D. simulans*. Thus, although copy number is correlated in the two species, the relative numbers of each family are significantly heterogeneous between species, *p* < 10^-8^. TEs grouped by class (*LTR*, *non-LTR*, *IR* and *FB*; Table S33) also show the positive correlation and (with the exception of *FB*) significant heterogeneity between species. Therefore, the overall picture is that relative family abundance between these sibling species is moderately conserved. While ascertainment of the *D. simulans* clustered TEs is difficult to quantify, these data suggest that that the impression that *D. simulans* TE copy number is much lower than that of *D. melanogaster* [2] may be partially attributable to ascertainment bias resulting from the focus on the most abundant *D. melanogaster* TE families.

**Genomic distribution and population variation**: The relative copy numbers of clustered TEs show small amounts of variation across the six *D. simulans* assemblies (Table S35), which is consistent with earlier findings [3]. Most significant is the variation among the four classes (pooled across families), which is probably attributable to the interaction of variation in coverage and variation in sequence length of copies within families of *IR* TEs. For example, the *TRANSIB2* transposable element family (155 copies detected), which contains many extensively deleted, putatively nonautonomous copies in *D. melanogaster* [4], is highly overrepresented (50% excess) in *sim4/6* and *w501*, the two most deeply sequenced lines.

Relative numbers of TEs in different families are heterogeneous across the five major chromosome arms (Table S36). Table S37 shows that this heterogeneity is not primarily due to different copy numbers on the *X* chromosome. Indeed, most of the heterogeneity is due to *non-LTR* and *IR* elements, both of which exhibit considerable variation in sequence length of individual insertions. TE copy number shows a consistent and significant (outliers removed) centromere-to-telomere decline on each chromosome arm (Figure S5). Although similar in direction, this pattern is weaker than the chromosomal gradients of nucleotide divergence and GC content. Table S38 shows the expected (based on coverage) and observed numbers of “cloned” TEs in exons (including UTRs), introns and intergenic sequence. As expected, TEs are dramatically underrepresented in exons, moderately underrepresented in introns, and overrepresented in intergenic sequence. This pattern is consistent across chromosome arms and lines.

Although clustered TEs cannot be confidently attached to small, gene-based annotations, they can be compared to nucleotide and indel polymorphism and divergence in 30 kb windows (Materials and Methods). Table S37 shows that windows containing a clustered TE are significantly more diverged than those that do not, despite the fact that such windows do not show a lower density of exons. The association of clustered TEs with heterozygosity is less consistent across arms, particularly for the X chromosome. Overall, these analyses suggest that TEs are more abundant in more diverged sequence or in sequences where large insertions are less likely to have a large fitness effect. Of course, regions of high divergence and/or less transcription could also be more likely target of insertion and/or less susceptible to ectopic recombination, the proposed mechanism containing TEs copy number in Drosophila populations [3].

The rarity of individual TE insertions in natural populations of *D. melanogaster* and *D. simulans* [3] stands in stark contrast to the pattern observed in mammals, in which most copies of TEs are fixed. Mapping of the clustered TEs is too crude to support confident detection of particular TE insertions that appear in multiple lines. However, “cloned” TEs, though small in number, are consistent with the idea that individual insertions are rare. Of the 434 cloned, euchromatic TEs, 16 are found in two lines, while two are found in three lines. Two of the 18 recurrent TEs are located in regions of strongly reduced polymorphism relative to divergence. A DMTHB1#1653 at 17757090 on *3L*, in lines *SIM4/6* and *MD199S*, is in a broad (>100 kb) region of extremely reduced polymorphism (Figure 1). Similarly, a *G5* element, also in lines *SIM4/6* and *MD199S*, is found at 9527700 on the *X*, which is the center of a 50 kb region of reduced polymorphism. These two instances suggest that some TEs that have reached appreciable frequencies may have recently hitchhiked in association with directional selection at linked sites.

Literature Cited

1. Kaminker JS, Bergman CM, Kronmiller B, Carlson J, Svirskas R, et al. (2002) The transposable elements of the Drosophila melanogaster euchromatin: a genomics perspective. Genome Biol 3: RESEARCH0084.

2. Dowsett AP, Young MW (1982) Differing levels of dispersed repetitive DNA among closely related species of Drosophila. Proc Natl Acad Sci U S A 79: 4570-4574.

3. Charlesworth B, Langley CH (1989) The population genetics of Drosophila transposable elements. Annu Rev Genet 23: 251-287.

4. Kapitonov VV, Jurka J (2003) Molecular paleontology of transposable elements in the Drosophila melanogaster genome. Proc Natl Acad Sci U S A 100: 6569-6574.
