# Supplementary material for: Population Genomics: Whole-Genome Analysis of Polymorphism and Divergence in Drosophila simulans
Source: PLoS Biol. 2007 Nov 6;5(11):e310. doi: 10.1371/journal.pbio.0050310 (PMC2062478; doi:10.1371/journal.pbio.0050310)
Supplement: Supplementary file 7 [file pbio.0050310.st001.doc]

Table S1. Coding and noncoding nucleotide heterozygosity in *D. simulans*, lineage-specific nucleotide divergence in *D. simulans*, *D. melanogaster*, *D. yakuba* and pairwise nucleotide divergence for *D. simulans/D. melanogaster*. UTRs are from the Gold collection genes.

|  | Chromosome | π | Div._mel-sim_ | Div._mel_ | Div._sim_ | Div._yak+(mel-sim anc)_ |
| --- | --- | --- | --- | --- | --- | --- |
| Non-synonymous | X | 0.0018 (1.14x10^-8^) | 0.0162 (8.59x10^-7^) | 0.0067 (9.79x10^-8^) | 0.0070 (1.99x10^-7^) | 0.0253 (2.05x10^-6^) |
|  | Autosomes | 0.0026 (3.69x10^-9^) | 0.0131 (7.99x10^-8^) | 0.0061 (1.54x10^-8^) | 0.0057 (2.29x10^-8^) | 0.0223 (2.18x10^-7^) |
|  |  |  |  |  |  |  |
|  | 2L | 0.0026 (1.46x10^-8^) | 0.0137 (4.73x10^-7^) | 0.0062 (6.82x10^-8^) | 0.0060 (1.09x10^-7^) | 0.0236 (1.21x10^-6^) |
|  | 2R | 0.0025 (2.40x10^-8^) | 0.0131 (3.07x10^-7^) | 0.0061 (6.26x10^-8^) | 0.0056 (8.99x10^-8^) | 0.0220 (9.18x10^-7^) |
|  | 3L | 0.0025 (1.46x10^-8^) | 0.0127 (3.68x10^-7^) | 0.0060 (8.59x10^-8^) | 0.0054 (1.12x10^-7^) | 0.0208 (9.04x10^-7^) |
|  | 3R | 0.0026 (8.41x10^-9^) | 0.0130 (2.07x10^-7^) | 0.0062 (4.02x10^-8^) | 0.0059 (6.64x10^-8^) | 0.0227 (6.04x10^-7^) |
|  |  |  |  |  |  |  |
| Synonmyous | X | 0.0199 (2.06x10^-7^) | 0.1161 (3.03x10^-6^) | 0.0767 (2.87x10^-6^) | 0.0519 (1.93x10^-6^) | 0.2314 (4.98x10^-5^) |
|  | Autosomes | 0.0352 (8.30x10^-8^) | 0.1167 (3.56x10^-7^) | 0.0695 (2.42x10^-7^) | 0.0524 (5.29x10^-7^) | 0.2187 (3.24x10^-6^) |
|  |  |  |  |  |  |  |
|  | 2L | 0.0359 (4.13x10^-7^) | 0.1188 (1.89x10^-6^) | 0.0701 (9.89x10^-7^) | 0.0528 (6.07x10^-7^) | 0.2226 (7.14x10^-6^) |
|  | 2R | 0.0349 (3.85x10^-7^) | 0.1168 (1.47x10^-6^) | 0.0705 (9.65x10^-7^) | 0.0531 (7.13x10^-7^) | 0.2201 (1.02x10^-5^) |
|  | 3L | 0.0354 (3.59x10^-7^) | 0.1155 (1.49x10^-6^) | 0.0678 (1.38x10^-6^) | 0.0517 (7.37x10^-6^) | 0.2167 (3.15x10^-5^) |
|  | 3R | 0.0349 (2.25x10^-7^) | 0.1161 (1.03x10^-6^) | 0.0697 (6.62x10^-7^) | 0.0523 (4.71x10^-7^) | 0.2162 (5.70x10^-6^) |
|  |  |  |  |  |  |  |
| Intron | X | 0.0166 (2.90x10^-6^) | 0.0657 (1.20x10^-5^) | 0.0248 (3.27x10^-6^) | 0.0330 (7.73x10^-6^) | 0.1175 (4.89x10^-5^) |
|  | Autosomes | 0.0212 (9.22x10^-7^) | 0.0584 (2.95x10^-6^) | 0.0240 (1.04x10^-6^) | 0.0281 (1.77x10^-6^) | 0.1028 (1.07x10^-5^) |
|  |  |  |  |  |  |  |
|  | 2L | 0.0221 (4.89x10^-6^) | 0.0594 (1.52x10^-5^) | 0.0244 (4.91x10^-6^) | 0.0292 (9.95x10^-6^) | 0.1063 (5.16x10^-5^) |
|  | 2R | 0.0206 (3.26x10^-6^) | 0.0578 (1.15x10^-5^) | 0.0235 (4.38x10^-6^) | 0.0275 (6.10x10^-6^) | 0.1021 (3.93x10^-5^) |
|  | 3L | 0.0220 (4.00x10^-6^) | 0.0592 (1.19x10^-5^) | 0.0239 (4.11x10^-6^) | 0.0284 (7.38x10^-6^) | 0.1024 (4.93x10^-5^) |
|  | 3R | 0.0203 (2.85x10^-6^) | 0.0575 (9.21x10^-6^) | 0.0242 (3.30x10^-6^) | 0.0272 (5.45x10^-6^) | 0.1011 (3.35x10^-5^) |
|  |  |  |  |  |  |  |
| g5’UTR | X | 0.0079 (3.30x10^-7^) | 0.0526 (4.85x10^-6^) | 0.0233 (1.67x10^-6^) | 0.0258 (2.10x10^-6^) | 0.1018 (1.44x10^-5^) |
|  | Autosomes | 0.0108 (1.02x10^-7^) | 0.0442 (7.58x10^-7^) | 0.0216 (2.30x10^-7^) | 0.0203 (2.97x10^-7^) | 0.0842 (2.43x10^-6^) |
|  |  |  |  |  |  |  |
|  | 2L | 0.0117 (4.49x10^-7^) | 0.0461 (2.99x10^-6^) | 0.0220 (9.12x10^-7^) | 0.0218 (1.30x10^-6^) | 0.0887 (1.03x10^-5^) |
|  | 2R | 0.0104 (4.74x10^-7^) | 0.0432 (3.72x10^-6^) | 0.0203 (9.54x10^-7^) | 0.0199 (1.36x10^-6^) | 0.0841 (1.20x10^-5^) |
|  | 3L | 0.0104 (4.27x10^-7^) | 0.0431 (3.27x10^-6^) | 0.0216 (9.49x10^-7^) | 0.0195 (1.05x10^-6^) | 0.0804 (8.75x10^-6^) |
|  | 3R | 0.0108 (3.07x10^-7^) | 0.0447 (2.33x10^-6^) | 0.0224 (8.24x10^-7^) | 0.0202 (1.03x10^-6^) | 0.0843 (8.10x10^-6^) |
|  |  |  |  |  |  |  |
| g3’UTR | X | 0.0088 (6.55x10^-7^) | 0.0503 (5.66x10^-6^) | 0.0199 (2.80x10^-8^) | 0.0261 (9.04x10^-8^) | 0.0957 (5.15x10^-7^) |
|  | Autosomes | 0.0113 (1.72x10^-7^) | 0.0418 (1.03x10^-6^) | 0.0186 (1.58x10^-7^) | 0.0192 (3.19x10^-7^) | 0.0775 (2.28x10^-6^) |
|  |  |  |  |  |  |  |
|  | 2L | 0.0122 (8.79x10^-7^) | 0.0432 (3.97x10^-6^) | 0.0186 (3.18x10^-8^) | 0.0200 (4.86x10^-8^) | 0.0815 (4.60x10^-7^) |
|  | 2R | 0.0099 (6.82x10^-7^) | 0.0397 (4.74x10^-6^) | 0.0177 (4.93x10^-8^) | 0.0185 (1.04x10^-7^) | 0.0735 (5.65x10^-7^) |
|  | 3L | 0.0120 (6.97x10^-7^) | 0.0411 (4.03x10^-6^) | 0.0180 (3.24x10^-8^) | 0.0187 (6.96x10^-8^) | 0.0741 (5.00x10^-7^) |
|  | 3R | 0.0112 (5.35x10^-7^) | 0.0430 (3.64x10^-6^) | 0.0198 (4.44x10^-8^) | 0.0195 (9.95x10^-8^) | 0.0809 (7.42x10^-7^) |
|  |  |  |  |  |  |  |
| Intergenic | X | 0.0153 (7.50x10^-7^) | 0.0620 (4.24x10^-6^) | 0.0231 (6.85x10^-7^) | 0.0299 (2.21x10^-6^) | 0.1102 (1.43x10^-5^) |
|  | Autosomes | 0.0204 (2.54x10^-7^) | 0.0561 (1.18x10^-6^) | 0.0225 (1.95x10^-7^) | 0.0265 (6.00x10^-7^) | 0.0957 (3.85x10^-6^) |
|  |  |  |  |  |  |  |
|  | 2L | 0.0209 (1.15x10^-6^) | 0.0564 (5.14x10^-6^) | 0.0227 (8.05x10^-7^) | 0.0270 (2.86x10^-6^) | 0.0979 (1.63x10^-5^) |
|  | 2R | 0.0200 (9.62x10^-7^) | 0.0558 (4.59x10^-6^) | 0.0219 (7.29x10^-7^) | 0.0265 (2.22x10^-6^) | 0.0970 (1.51x10^-5^) |
|  | 3L | 0.0222 (1.27x10^-6^) | 0.0578 (5.41x10^-6^) | 0.0228 (9.18x10^-7^) | 0.0277 (2.56x10^-6^) | 0.0966 (1.81x10^-5^) |
|  | 3R | 0.0187 (7.34x10^-7^) | 0.0547 (3.66x10^-6^) | 0.0226 (6.44x10^-7^) | 0.0252 (1.92x10^-6^) | 0.0924 (1.20x10^-5^) |
|  |  |  |  |  |  |  |
| Deletions | X | 0.0023 | 0.0060 |  |  |  |
|  | Autosomes | 0.0021 | 0.0035 |  |  |  |
|  |  |  |  |  |  |  |
|  | 2L | 0.0022 | 0.0037 |  |  |  |
|  | 2R | 0.0020 | 0.0035 |  |  |  |
|  | 3L | 0.0023 | 0.0037 |  |  |  |
|  | 3R | 0.0019 | 0.0031 |  |  |  |
|  |  |  |  |  |  |  |
| Insertions | X | 0.0019 | 0.0036 |  |  |  |
|  | Autosomes | 0.0018 | 0.0026 |  |  |  |
|  |  |  |  |  |  |  |
|  | 2L | 0.0018 | 0.0026 |  |  |  |
|  | 2R | 0.0017 | 0.0026 |  |  |  |
|  | 3L | 0.0019 | 0.0028 |  |  |  |
|  | 3R | 0.0017 | 0.0026 |  |  |  |
|  |  |  |  |  |  |  |
| 50 kb Windows | X | 0.0135 |  | 0.0232 | 0.0277 | 0.1017 |
|  | Autosomes | 0.0180 |  | 0.0224 | 0.0243 | 0.0892 |
|  |  |  |  |  |  |  |
|  | 2L | 0.0187 |  | 0.0227 | 0.0252 | 0.0923 |
|  | 2R | 0.0171 |  | 0.0218 | 0.0237 | 0.0881 |
|  | 3L | 0.0192 |  | 0.0225 | 0.0250 | 0.0896 |
|  | 3R | 0.0170 |  | 0.0224 | 0.0235 | 0.0871 |
