# Supplementary material for: Population Genomics: Whole-Genome Analysis of Polymorphism and Divergence in Drosophila simulans
Source: PLoS Biol. 2007 Nov 6;5(11):e310. doi: 10.1371/journal.pbio.0050310 (PMC2062478; doi:10.1371/journal.pbio.0050310)
Supplement: Supplementary file 8 [file pbio.0050310.st002.doc]

Table S2. Nonsynonymous (NS) and synonymous (S) variants in heterochromatic vs. euchromatic genes.

|  | Euchromatic | | |  | Heterochromatic | | |
| --- | --- | --- | --- | --- | --- | --- | --- |
|  | Polymorphism | Fixation | Poly/Fix |  | Polymorphism | Fixation | Poly/Fix |
| NS | 23599 | 29254 | 0.81 |  | 394 | 1273 | 0.31 |
| S | 143076 | 81554 | 1.75 |  | 1438 | 3024 | 0.48 |
| NI |  |  | 0.46 |  |  |  | 0.65 |
| NS/S | 0.16 |  |  |  | 0.27 |  |  |

NI = (NSfix/NSpoly)/(Sfix/Spoly)
