# Supplementary material for: Population Genomics: Whole-Genome Analysis of Polymorphism and Divergence in Drosophila simulans
Source: PLoS Biol. 2007 Nov 6;5(11):e310. doi: 10.1371/journal.pbio.0050310 (PMC2062478; doi:10.1371/journal.pbio.0050310)
Supplement: Supplementary file 9 [file pbio.0050310.st003.doc]

Table S3. Comparisons of *D. simulans* vs *D. melanogaster* divergence and X vs autosome divergence for *D. simulans*, *D. melanogaster* and *D. yakuba*.

| Feature | Divergence Comparison |  | Feature | Divergence Comparison |
| --- | --- | --- | --- | --- |
| 5’UTR | sim < mel**  A: sim < mel****  X: sim = mel |  | dN | sim < mel****  A: sim < mel****  X: sim > mel* |
|  |  |  |  |  |
|  | sim X > A****  mel X > A*  yak X > A**** |  |  | sim X = A  mel X = A  yak X > A* |
|  |  |  |  |  |
| 3’UTR | sim > mel**  A: sim = mel  X: sim > mel**** |  | dS | sim < mel****  A: sim < mel****  X: sim < mel**** |
|  |  |  |  |  |
|  | sim X > A****  mel X > A*  yak X > A**** |  |  | sim X = A  mel X > A****  yak X > A**** |
|  |  |  |  |  |
| Intron | sim > mel****  A: sim > mel****  X: sim > mel**** |  | 50 kb windows | sim > mel****  A: sim > mel****  X: sim > mel**** |
|  |  |  |  |  |
|  | sim X = A  mel X < A****  yak X > A**** |  |  | sim X > A****  mel X > A****  yak X > A*** |
|  |  |  |  |  |
| Intergenic | sim > mel****  A: sim > mel****  X: sim > mel**** |  |  |  |
|  |  |  |  |  |
|  | sim X > A***  mel X < A****  yak X > A**** |  |  |  |

*D. simulans* vs. *D. melanogaster* inferences are Wilcoxon sign rank tests: X vs A inferences are Mann Whitney U tests.

* p < 0.05, ** p < 0.01, ***p < 0.001, **** p < 0.0001
