# Supplementary material for: Population Genomics: Whole-Genome Analysis of Polymorphism and Divergence in Drosophila simulans
Source: PLoS Biol. 2007 Nov 6;5(11):e310. doi: 10.1371/journal.pbio.0050310 (PMC2062478; doi:10.1371/journal.pbio.0050310)
Supplement: Supplementary file 10 [file pbio.0050310.st004.doc]

Table S4. Comparison of X and autosome polarized polymorphic variants in different frequency classes for sites with coverage *n* = 5 or *n* = 6 *D. simulans* alleles.

|  | X | A | X/A |
| --- | --- | --- | --- |
| Coverage-five sites |  |  |  |
| 1 | 19224 | 220864 | 0.087 |
| 2 | 6200 | 69352 | 0.089 |
| 3 | 4091 | 37915 | 0.108 |
| 4 | 3510 | 31251 | 0.112 |
| χ^2^ = 279.32, df=3, p < 0.0001 |  |  |  |
|  |  |  |  |
| Fixed | 26503 | 152491 | 0.174 |
|  |  |  |  |
| Coverage-six sites |  |  |  |
| 1 | 5259 | 80848 | 0.065 |
| 2 | 1624 | 25094 | 0.065 |
| 3 | 1013 | 13408 | 0.076 |
| 4 | 767 | 9367 | 0.082 |
| 5 | 778 | 9472 | 0.082 |
| χ^2^ = 73.7, df=4, p < 0.0001 |  |  |  |
|  |  |  |  |
| Fixed | 6583 | 52202 | 0.126 |
