# Supplementary material for: Population Genomics: Whole-Genome Analysis of Polymorphism and Divergence in Drosophila simulans
Source: PLoS Biol. 2007 Nov 6;5(11):e310. doi: 10.1371/journal.pbio.0050310 (PMC2062478; doi:10.1371/journal.pbio.0050310)
Supplement: Supplementary file 11 [file pbio.0050310.st005.doc]

Table S5. Spearman correlations of nucleotide heterozygosity, nucleotide divergence, relative rate χ^2^ tests, ancestral GC content and *D. simulans* transposable element density (all measured in 50 kb windows) vs. proximal-distal location along chromosome arms. Positive correlations for 2L, 3L and X, and negative correlations for 2R and 3R indicate increasing values closer to centromeres. Inv3R was used for *D. simulans* lineage inferences. Trimmed data indicates analyses for which regions of low heterozygosity were removed (Methods).

*D. simulans* divergence

X	0.18**

2L	0.24***

2R	-0.30***

3L	0.40***

3R	-0.47***

*D. simulans* divergence (trimmed)

X	0.05 NS

2L	0.13*

2R	-0.29***

3L	0.48***

3R	-0.44***

*D. simulans* relative rate χ^2^

X	-0.29***

2L	0.01 NS

2R	-0.04 NS

3L	0.19***

3R	-0.14*

*D. simulans* relative rate χ^2^ (trimmed)

X	-0.20***

2L	0.03 NS

2R	-0.05 NS

3L	0.13*

3R	-0.09*

*D. simulans* nucleotide heterozygosity

X	0.32***

2L	-0.02 NS

2R	-0.19**

3L	0.26***

3R	-0.17***

*D. simulans* nucleotide heterozygosity (trimmed)

X	0.08 NS

2L	-0.02 NS

2R	-0.19**

3L	0.43***

3R	-0.17***

Inferred ancestral GC content

X	-0.10 NS

2L	-0.36***

2R	0.06 NS

3L	-0.19***

3R	0.24***

Inferred ancestral GC composition (trimmed)

X	0.08 NS

2L	-0.26***

2R	-0.20**

3L	-0.26***

3R	0.22***

TE density

X	-0.620,10^-14^

X*	-0.623,10^-14^

2L	+0.175,0.08

2R	-0.309, 0.003

3L	+0.079, 0.4

3L*	+0.119,0.23

3R	-0.404,10^-5^

*Outlier windows removed, 2 for *3L* and 4 for *X*.

*D. melanogaster* divergence

X	0.01 NS

2L	0.09 NS

2R	-0.13*

3L	0.34***

3R	-0.38***

*D. melanogaster* divergence (trimmed)

X	-0.08 NS

2L	0.03 NS

2R	-0.18**

3L	0.26***

3R	-0.35****

*D. melanogaster* relative rate χ^2^

X	-0.20***

2L	0.09 NS

2R	-0.06 NS

3L	0.02 NS

3R	-0.03 NS

*D. melanogaster* relative rate χ^2^ (trimmed)

X	-0.20***

2L	0.00 NS

2R	-0.11*

3L	-0.12*

3R	-0.06 NS

*** p < 0.0001

** p <0.001

* p < 0.05

NS = not significant
