# Supplementary material for: Population Genomics: Whole-Genome Analysis of Polymorphism and Divergence in Drosophila simulans
Source: PLoS Biol. 2007 Nov 6;5(11):e310. doi: 10.1371/journal.pbio.0050310 (PMC2062478; doi:10.1371/journal.pbio.0050310)
Supplement: Supplementary file 12 [file pbio.0050310.st006.doc]

Table S6. Autocorrelations of *D. simulans* nucleotide polymorphism and divergence (10 and 50 kb windows) along chromosome arms. All are significant at p < 0.0001.

| **Polarized Divergence** | | |  | **Polymorphism** | | |
| --- | --- | --- | --- | --- | --- | --- |
| *Chromosome* | *Window size* | *r* |  | *Chromosome* | *Window size* | *r* |
| X | 10 | 0.423 |  | X | 10 | 0.610 |
|  | 50 | 0.456 |  |  | 50 | 0.680 |
| 2L | 10 | 0.268 |  | 2L | 10 | 0.501 |
|  | 50 | 0.286 |  |  | 50 | 0.536 |
| 2R | 10 | 0.358 |  | 2R | 10 | 0.476 |
|  | 50 | 0.358 |  |  | 50 | 0.464 |
| 3L | 10 | 0.485 |  | 3L | 10 | 0.608 |
|  | 50 | 0.527 |  |  | 50 | 0.612 |
| 3R | 10 | 0.380 |  | 3R | 10 | 0.495 |
|  | 50 | 0.457 |  |  | 50 | 0.548 |
