# Supplementary material for: Population Genomics: Whole-Genome Analysis of Polymorphism and Divergence in Drosophila simulans
Source: PLoS Biol. 2007 Nov 6;5(11):e310. doi: 10.1371/journal.pbio.0050310 (PMC2062478; doi:10.1371/journal.pbio.0050310)
Supplement: Supplementary file 13 [file pbio.0050310.st007.doc]

Table S7. Gene Ontology terms overrepresented among genes in HKA windows having unusually low ratios of nucleotide heterozygosity to divergence. CC, MF, and BP are Cellular Component, Molecular Function and Biological Process, respectively**.**

| GOID | Numgenes | GO Term | pval | GO |
| --- | --- | --- | --- | --- |
| GO:0005635 | 10 | nuclear envelope | 0.011 | CC |
| GO:0005643 | 8 | nuclear pore | 0.007 | CC |
| GO:0005634 | 139 | nucleus | 0.001 | CC |
| GO:0016469 | 8 | proton-transporting two-sector ATPase complex | 0.004 | CC |
| GO:0004197 | 8 | cysteine-type endopeptidase activity | 0.030 | MF |
| GO:0003677 | 66 | DNA binding | 0.019 | MF |
| GO:0003678 | 7 | DNA helicase activity | 0.018 | MF |
| GO:0046933 | 10 | hydrogen-transporting ATP synthase activity, rotational mechanism | <0.001 | MF |
| GO:0046961 | 10 | hydrogen-transporting ATPase activity, rotational mechanism | <0.001 | MF |
| GO:0003676 | 118 | nucleic acid binding | 0.042 | MF |
| GO:0003704 | 10 | specific RNA polymerase II transcription factor activity | 0.005 | MF |
| GO:0003700 | 35 | transcription factor activity | 0.018 | MF |
| GO:0030528 | 67 | transcription regulator activity | 0.005 | MF |
| GO:0004843 | 5 | ubiquitin-specific protease activity | 0.005 | MF |
| GO:0051082 | 9 | unfolded protein binding | 0.034 | MF |
| GO:0004221 | 5 | ubiquitin thiolesterase activity | 0.028 | MF |
| GO:0009948 | 8 | anterior/posterior axis specification | <0.001 | BP |
| GO:0015986 | 10 | ATP synthesis coupled proton transport | 0.001 | BP |
| GO:0001709 | 5 | cell fate determination | 0.015 | BP |
| GO:0009790 | 26 | embryonic development | 0.036 | BP |
| GO:0007507 | 6 | heart development | 0.045 | BP |
| GO:0007494 | 5 | midgut development | 0.005 | BP |
| GO:0006139 | 114 | nucleobase, nucleoside, nucleotide and nucleic acid metabolism | 0.028 | BP |
| GO:0016579 | 5 | protein deubiquitination | 0.013 | BP |
| GO:0000381 | 7 | regulation of alternative nuclear mRNA splicing, via spliceosome | 0.042 | BP |
| GO:0006521 | 5 | regulation of amino acid metabolism | 0.003 | BP |
| GO:0006355 | 23 | regulation of transcription, DNA-dependent | 0.038 | BP |
| GO:0006790 | 8 | sulfur metabolism | 0.010 | BP |
| GO:0006366 | 24 | transcription from RNA polymerase II promoter | 0.021 | BP |
|  |  |  |  |  |
|  |  | >=2windows, log pvalue <=-6, 1000 permuted ds |  |  |

| GOID | NumGenes | GO Term | p-val |  |
| --- | --- | --- | --- | --- |
| GO:0005843 | 6 | cytosolic small ribosomal subunit (sensu Eukaryota) | 0.043 | CC |
| GO:0005622 | 159 | intracellular | 0.039 | CC |
| GO:0005635 | 11 | nuclear envelope | 0.001 | CC |
| GO:0005643 | 6 | nuclear pore | 0.031 | CC |
| GO:0005634 | 107 | nucleus | 0.006 | CC |
| GO:0005488 | 142 | binding | 0.014 | MF |
| GO:0005516 | 8 | calmodulin binding | 0.038 | MF |
| GO:0003678 | 5 | DNA helicase activity (could also be DNA binding) | 0.050 | MF |
| GO:0003704 | 10 | specific RNA polymerase II transcription factor activity | 0.008 | MF |
| GO:0003735 | 18 | structural constituent of ribosome | 0.017 | MF |
| GO:0030528 | 54 | transcription regulator activity | 0.029 | MF |
| GO:0051082 | 8 | unfolded protein binding | 0.028 | MF |
| GO:0009948 | 6 | anterior/posterior axis specification | 0.000 | BP |
| GO:0007420 | 6 | brain development | 0.000 | BP |
| GO:0006887 | 7 | exocytosis | 0.023 | BP |
| GO:0006139 | 96 | nucleobase, nucleoside, nucleotide and nucleic acid metabolism | 0.025 | BP |
| GO:0006412 | 38 | protein biosynthesis | 0.022 | BP |
| GO:0000059 | 5 | protein import into nucleus, docking | 0.009 | BP |
|  |  |  |  |  |
|  |  | >=4 windows, log pval <=-2, from 1000 permuted ds. |  |  |

**GO clustering**

We calculated the average number of instances of each GO term in 1MB non-overlapping windows. We also calculated at standard deviation of the average. We then compared the coefficient of variation (SD/mean) of each GO term to 1000 random datasets with the locations of genes permuted. Overall, 30% of GO terms are significantly clustered in the genome. However, GO terms in the windows showing reduced polymorphism relative to divergence are significantly clustered 55-60% of the time.
