# Supplementary material for: Population Genomics: Whole-Genome Analysis of Polymorphism and Divergence in Drosophila simulans
Source: PLoS Biol. 2007 Nov 6;5(11):e310. doi: 10.1371/journal.pbio.0050310 (PMC2062478; doi:10.1371/journal.pbio.0050310)
Supplement: Supplementary file 14 [file pbio.0050310.st008.doc]

Table S8. Mean (SE) ratio of nucleotide heterozygosity (50 kb windows, weighted by coverage) for New World vs. Old World lines.

X	0.531 (0.027)

2L	0.740 (0.016)

2R	0.727 (0.017)

3L	0.801 (0.013)

3R	0.771 (0.015)
