# Supplementary material for: Population Genomics: Whole-Genome Analysis of Polymorphism and Divergence in Drosophila simulans
Source: PLoS Biol. 2007 Nov 6;5(11):e310. doi: 10.1371/journal.pbio.0050310 (PMC2062478; doi:10.1371/journal.pbio.0050310)
Supplement: Supplementary file 15 [file pbio.0050310.st009.doc]

Table S9. Regions of the genome showing disproportionate reductions of nucleotide heterozygosity in the US sample.

X1:170000-249999 *

X2:9130000-9269999 ***

X3:9390000-9469999 *

X4:10230000-10289999

2R1:2190000-2289999 *

2R2:5650000-5709999

2R3:5750000-5809999

2R4:18850000-18909999

2L1:300000-419999 **

2L2:1320000-1379999

2L3:2040000-2159999 **

2L4:2280000-2339999

2L5:10340000-10399999

2L6:11060000-11119999

3R1:2050000-2109999

3R2:4450000-4529999

3R3:5110000-5169999

3R4:16950000-17009999

3R5:23190000-23249999

3R6:26750000-26829999

3L1:3010000-3069999

3L2:3130000-3209999 *

3L3:5130000-5189999

3L4:16310000-16369999

All p < 0.05.

Asterisks indicate Bonferroni corrected p values * = < 0.05, ** = < 0.01, *** = < 0.001
