# Supplementary material for: Population Genomics: Whole-Genome Analysis of Polymorphism and Divergence in Drosophila simulans
Source: PLoS Biol. 2007 Nov 6;5(11):e310. doi: 10.1371/journal.pbio.0050310 (PMC2062478; doi:10.1371/journal.pbio.0050310)
Supplement: Supplementary file 16 [file pbio.0050310.st010.doc]

Table S10. Genes located in genomics regions showing disproportionate reductions of nucleotide heterozygosity in the US sample.

X1*

X:171945..173379	*Or1a*

X:173843..191621	*CG32816*

X:136525..207026	*CG3777*

X:213331..218067	*y*

X:226853..227813	*ac*

X2***

X:9129870..9148858	*lz*

X:9157064..9158135	*CG12667*

X:9163135..9164465	*c12.1*

X:9164713..9169580	*c12.2*

X:9171879..9184385	*CG32705*

X:9201867..9203823	*BCL7-like*

X:9205052..9221083	*CG12139*

X:9227659..9229457	*CG12654*

X:9256913..9264208	*CG12650*

X3*

X:9351932..9396450	*CG32700*

X:9396826..9398891	*l(1)G0320*

X:9399840..9400442	*RpS28b*

X:9401003..9401697	*CG15317*

X:9403886..9416369	*CG32702*

X:9418018..9425196	*CG32699*

X:9431106..9433625	*Hex-A*

X:9446255..9449503	*Gga*

X:9449373..9451187	*CG3004*

X:9451581..9454105	*Aats-lys*

X:9454181..9457826	*CG3003*

X:9464974..9466888	*CG3099*

X4

X:10228837..10230355	*CG2222*

X:10231154..10253307	*flw*

X:10262717..10263304	*RabX2*

X:10277686..10278033	*CG12640*

2R1*

2R:2188809..2192199	*Tdc1*

2R:2195052..2196170	*CG15909*

2R:2196785..2201775	*Tdc2*

2R:2187640..2204407	*CG9422*

2R:2205253..2207000	*CG3270*

2R:2206927..2208438	*CG3403*

2R:2208535..2211749	*Rab2*

2R:2211933..2227678	*CG3409*

2R:2228158..2229620	*ZIP1*

2R:2230429..2231346	*CG9430*

2R:2232912..2252457	*l(2)01289*

2R:2253680..2257957	*phtf*

2R:2258198..2260767	*CG3267*

2R:2261005..2267852	*Eb1*

2R:2268103..2268833	*CG3420*

2R:2269414..2270699	*CG9436*

2R2

2R:5649792..5650812	*CG12917*

2R:5651240..5653847	*Or46a*

2R:5654153..5658025	*CG18011*

2R:5657706..5666684	*KCNQ*

2R:5668183..5670693	*CG12214*

2R3

2R:5752682..5757896	*Hdc*

2R:5757886..5759285	*CG12209*

2R:5759945..5760973	*CG12914*

2R:5761290..5764222	*Syb*

2R:5761069..5766657	*CG12913*

2R:5768502..5773089	*CG12911*

2R:5773128..5775639	*CR30009*

2R:5773128..5775639	*CR30009-RA*

2R:5778682..5781020	*CG12910*

2R4

2R:18851005..18855237	*CG3502*

2R:18854235..18855377	*CG9863*

2R:18862075..18862832	*CG30409*

2R:18862829..18863798	*CG30410*

2R:18863969..18865165	*CG3500*

2R:18865279..18866145	*CG9875*

2R:18866358..18867873	*CG11079*

2R:18867863..18873854	*vir*

2R:18874489..18879061	*CG13550*

2R:18878951..18884612	*CG3493*

2R:18886133..18888038	*yellow-d*

2R:18888799..18890193	*yellow-d2*

2R:18885460..18891021	*CG13551*

2R:18891243..18892926	*CG30414*

2R:18893272..18894025	*CG30415*

2R:18894126..18896656	*eIF2B-delta*

2R:18896606..18904279	*CG3530*

2R:18904366..18909909	*CG3520*

2L1**

2L:297882..303955	P*i3K21B*

2L:318357..319254	*CG11912*

2L:320279..321246	*CG11911*

2L:322131..322985	*CG33127*

2L:327429..328518	*CG33992*

2L:325520..329761	*CG31920*

2L:329991..332335	*CG31921*

2L:305941..346549	P*lc21C*

2L:355559..357868	*Ent1*

2L:378116..387439	a*l*

2L:396595..402181	*CG4213*

2L:404286..408049	*RpI135*

2L:408022..414775	a*lpha-Adaptin*

2L:415069..418536	e*bi*

2L:418557..419600	*CG13690*

2L:419931..420706	*RpLP1*

2L2

2L:1329161..1329904	*a5*

2L:1334269..1334763	*CG5440*

2L:1341365..1342095	*CG33923*

2L:1343443..1344171	*CG33922*

2L:1344480..1345144	*Cdlc2*

2L:1351860..1352869	*CG5556*

2L:1352970..1354109	*CG5561*

2L:1354098..1354919	*CG31924*

2L:1354950..1355924	*CG5565*

2L:1358971..1359784	*CG31659*

2L:1359983..1361732	*NLaz*

2L:1368102..1369047	*CG14346*

2L3**

2L:2037802..2044373	*Su(dx)*

2L:2045525..2048054	*CG31672*

2L:2054589..2057967	*Or22c*

2L:2080077..2081170	*CG12674*

2L:2061793..2109836	*dpr3*

2L:2128215..2129211	*CG4259*

2L:2130815..2137296	*GlyP*

2L:2137350..2145175	*tho2*

2L:2145491..2147287	*CG11723*

2L:2147519..2151664	*CG7261*

2L:2151664..2155390	*CG7263*

2L:2155760..2156791	*CG15382*

2L4

2L:2285174..2285902	*CG4271*

2L:2286050..2286844	*CG31681*

2L:2288495..2289370	*CG17237*

2L:2290329..2290622	*robl22E*

2L:2300119..2300717	*CG31949*

2L:2308027..2308671	*CG16995*

2L5

2L:10339460..10341505	*SamDC*

2L:10341621..10342527	*CG31715*

2L:10342711..10344228	*Sps2*

2L:10344459..10347706	*CG5022*

2L:10348017..10349744	*CG13141*

2L:10351580..10353631	*CG5369*

2L:10354810..10356276	*CG5367*

2L:10356743..10357876	*CG5045*

2L:10358162..10362703	*CG5366*

2L:10362977..10364105	*pim*

2L:10365118..10366149	*CG13139*

2L:10366698..10367829	*CG5056*

2L:10368176..10372898	*rho-5*

2L:10372955..10375752	*CG33303*

2L:10376492..10379908	*dpr19*

2L:10380118..10382865	*CG5091*

2L:10382253..10384730	*CG5096*

2L:10384739..10386263	*cdc2*

2L:10386664..10387524	*mRpS7*

2L:10388078..10392786	*da*

2L:10393847..10396299	*CG5362*

2L:10396711..10402885	*CG31716*

2L6

2L:11068776..11069508	*CG14914*

2L:11071061..11071423	*CG14915*

2L:11076152..11081397	*Ast-C*

2L:11083741..11085170	*CG14920*

2L:11088331..11090782	*CG16854*

2L:11091446..11095005	*CG4705*

2L:11095353..11100866	*CG6181*

2L:11100959..11106638	*CG6192*

2L:11106952..11110852	*CG4713*

2L:11110557..11113063	*CG6201*

2L:11114183..11115755	*Gr32a*

3R1

3R:2053428..2054461	*Osi5*

3R:2060431..2062603	*Osi6*

3R:2074714..2075795	*Osi7*

3R:2080997..2082114	*Osi8*

3R:2085935..2087832	*Osi9*

3R:2089581..2093117	*Osi10*

3R:2093502..2094410	*Osi11*

3R:2104253..2108217	*Osi12*

3R2

3R:4445477..4452743	*CG8007*

3R:4460192..4461606	*Or85e*

3R:4468055..4468147	*tRNA:Y1:85Ae-RA*

3R:4468055..4468147	*tRNA:Y1:85Ae*

3R:4468423..4468515	*tRNA:Y1:85Ad*

3R:4468423..4468515	*tRNA:Y1:85Ad-RA*

3R:4468782..4471179	*Cyp313b1*

3R:4473287..4476516	*Mkk4*

3R:4476839..4477114	*CG8021*

3R:4478738..4479576	*CG11753*

3R:4477111..4479736	*Dhod*

3R:4481292..4486483	*bel*

3R:4486622..4489607	*CG9770*

3R:4490085..4493076	*CG8032*

3R:4493838..4493930	*tRNA:Y1:85Ac-RA*

3R:4493838..4493930	*tRNA:Y1:85Ac*

3R:4494334..4494427	*tRNA:Y1:85Ab-RA*

3R:4494334..4494427	*tRNA:Y1:85Ab*

3R:4494803..4494896	*tRNA:Y1:85Aa-RA*

3R:4494803..4494896	*tRNA:Y1:85Aa*

3R:4493726..4499453	*CG8036*

3R:4499839..4500924	*mRpL19*

3R:4501005..4502310	*CG9773*

3R:4502639..4503955	*CG8043*

3R:4504007..4504471	*CG11755*

3R:4504676..4506052	*CG33325*

3R:4517038..4523540	*hb*

3R3

3R:5147548..5148072	*HP1e*

3R:5120655..5154227	*CG8861*

3R:5154998..5156047	*CG12951*

3R:5156601..5157617	*CG16749*

3R:5157783..5160436	*CG8866*

3R:5160897..5162987	*CG8121*

3R4

3R:16944973..16953202	*slmb*

3R:16953446..16954630	*CG5793*

3R:16954672..16955439	*Obp93a*

3R:16955941..16958402	*CG10825*

3R:16958456..16959599	*CG7009*

3R:16959984..16963938	*CG5798*

3R:16964409..16966178	*CG5802*

3R:16984955..16986815	*CG10824*

3R:16989512..16990798	*CG5810*

3R:16991436..16993850	*CG7000*

3R5

3R:23185112..23190052	*CG6599*

3R:23194867..23196702	*CG13980*

3R:23233587..23233880	*CG12509*

3R:23202304..23244247	*side*

3R6

3R:26738610..26756404	*Ptx1*

3R:26761118..26762229	*CG15549*

3R:26762389..26762820	*CG15550*

3R:26785529..26787973	*CG15548*

3R:26789011..26790042	*Ctr1C*

3R:26815199..26815728	*CG31008*

3R:26817457..26818179	*CG31007*

3L1

3L:3007661..3010053	*aly*

3L:3018730..3024003	*CG2162*

3L:3025907..3027713	*Jafrac2*

3L:3027637..3030342	*CG1893*

3L:3030488..3033693	*Sk2*

3L:3033861..3035297	*CG32485*

3L:3036046..3038439	*CG1271*

3L:3038634..3039376	*CG16753*

3L:3039436..3054371	*CG32486*

3L:3054660..3072206	*CG11486*

3L2*

3L:3128937..3131890	*CG12082*

3L:3132207..3138349	*BtbVII*

3L:3139253..3141940	*CG14956*

3L:3142022..3143105	*CG12079*

3L:3143527..3145556	*pfk*

3L:3145712..3147384	*rasp*

3L:3147311..3149782	*CG14962*

3L:3148714..3152612	*CG32280*

3L:3152907..3154441	*CG32281*

3L:3152819..3155299	*CG32278*

3L:3155288..3156073	*CG14963*

3L:3156795..3161438	*CG14964*

3L:3162466..3168822	*CG12734*

3L:3170196..3170781	*CG32284*

3L:3171111..3171522	*CG14957*

3L:3173526..3174068	*CG14958*

3L:3174153..3176000	*CG14965*

3L:3176559..3180593	*Hsp83*

3L:3180630..3181052	*CG14966*

3L:3181392..3182575	*CG32276*

3L:3185846..3186592	*CG32271*

3L:3187207..3187980	*CG33159*

3L:3188196..3188981	*CG32277*

3L:3189119..3189895	*CG33160*

3L:3190477..3192514	*CG32269*

3L:3190477..3192514	*CG32270*

3L:3182553..3194879	*gry*

3L:3194642..3202336	*CG14967*

3L:3202656..3204460	*CG12034*

3L:3204442..3206490	*RpL28*

3L:3206882..3208236	*CG17737*

3L3

3L:5156870..5158798	*CG10677*

3L4

3L:16304258..16310507	*CG4998*

3L:16311582..16312879	*CG33257*

3L:16313356..16314035	*mRpS34*

3L:16315176..16315248	*CR32357*

3L:16314004..16317157	*CG4925*

3L:16317299..16318549	*CG4933*

3L:16318486..16319556	*Pdh*

3L:16320004..16324684	*CG4893*

3L:16327862..16328939	*CG4818*

3L:16330256..16330909	*CG12255*

3L:16331150..16332585	*CG4784*

3L:16333666..16334777	*CG4842*

3L:16335135..16336102	*CG18814*

3L:16338312..16343909	*CG16807*

3L:16344229..16346829	*CG4753*

3L:16347030..16351216	*CG4729*

3L:16351599..16356083	*CG4877*

3L:16356381..16356940	*CG13036*

3L:16359888..16361601	*CG4680*

3L:16361821..16365316	*CG13035*
