# Supplementary material for: Population Genomics: Whole-Genome Analysis of Polymorphism and Divergence in Drosophila simulans
Source: PLoS Biol. 2007 Nov 6;5(11):e310. doi: 10.1371/journal.pbio.0050310 (PMC2062478; doi:10.1371/journal.pbio.0050310)
Supplement: Supplementary file 17 [file pbio.0050310.st011.doc]

Table S11. Gene Ontology terms overrepresented in windows from Out-of-Afr./Mad. analysis. MF and BP = molecular function and biological process, respectively.

| GO ID | Num Genes | GO Term | perm pval | GO |
| --- | --- | --- | --- | --- |
| GO:0003824 | 80 | catalytic activity | 0.019 | MF |
| GO:0004263 | 13 | chymotrypsin activity | 0.036 | MF |
| GO:0008233 | 16 | peptidase activity | 0.044 | MF |
| GO:0004252 | 10 | serine-type endopeptidase activity | 0.026 | MF |
| GO:0004295 | 13 | trypsin activity | 0.04 | MF |
| GO:0009056 | 28 | catabolism | 0.002 | BP |
| GO:0007154 | 37 | cell communication | 0.016 | BP |
| GO:0007267 | 13 | cell-cell signaling | 0.039 | BP |
| GO:0030154 | 10 | cell differentiation | 0.013 | BP |
| GO:0006091 | 5 | generation of precursor metabolites and energy | 0.032 | BP |
| GO:0008152 | 101 | metabolism | 0.002 | BP |
| GO:0019538 | 44 | protein metabolism | 0.005 | BP |
| GO:0006464 | 18 | protein modification | 0.05 | BP |
| GO:0006508 | 24 | proteolysis | 0.033 | BP |
| GO:0019226 | 9 | transmission of nerve impulse | 0.002 | BP |
