# Supplementary material for: Population Genomics: Whole-Genome Analysis of Polymorphism and Divergence in Drosophila simulans
Source: PLoS Biol. 2007 Nov 6;5(11):e310. doi: 10.1371/journal.pbio.0050310 (PMC2062478; doi:10.1371/journal.pbio.0050310)
Supplement: Supplementary file 18 [file pbio.0050310.st012.doc]

Table S12. Gene Ontology terms associated with the top 20 genes with the smallest unpolarized MK test p-value.

| Gene | CG | ARM | pval | Cellular Component | | Molecular Function | | Biological Process | |
| --- | --- | --- | --- | --- | --- | --- | --- | --- | --- |
| Pkd2 | CG6504-PA | 2L | 8.40E-11 | membrane | GO:0016020 | cation channel activity,  calcium ion binding | GO:0005261  GO:0005509 | cation transport,  cell homeostasis,  development,  ion transport,  larval feeding behavior,  smooth muscle contraction,  sperm competition,  sperm motility | GO:0006812  GO:0019725  GO:0007275  GO:0006811  GO:0030536  GO:0006939  GO:0046692  GO:0030317 |
| trol | ***CG33950-PF*** | X | 1.45E-08 | basement membrane,  extracellular matrix | GO:0005604  GO:0005578 | DNA binding,  structural molecule activity | GO:0003677 GO:0005198 | DNA methylation,  cell-cell adhesion,  cell-matrix adhesion,  neuroblast division,  signal transduction | GO:0006306 GO:0016337 GO:0007160 GO:0045034 GO:0007165 |
|  | *CG31731-PA* | 2L | 4.35E-08 | ATP-binding cassette transporter complex | GO:0043190 | ATP binding,  transporter activity,  ATPase activity coupled to transmembrane movement of substances,  nucleotide binding | GO:0005524 GO:0005215  GO:0042626  GO:0000166 | coenzyme metabolism,  prosthetic group metabolism,  transport | GO:0006732 GO:0051189 GO:0006810 |
| Nc | *CG8091-PA* | 3L | 4.66E-08 | NOT mitochondrion,  cytoplasm | GO:0005739 GO:0005737 | protein binding,  caspase activity | GO:0005515 GO:0030693 | apoptosis,  apoptotic program,  autophagic cell death,  embryonic development,  induction of programmed cell death by hormones,  metamorphosis,  proteolysis,  regulation of apoptosis,  regulation of retinal programmed cell death,  salivary gland cell autophagic cell death,  sperm individualization | GO:0006915 GO:0008632 GO:0048102 GO:0001700 GO:0035081  GO:0046698 GO:0006508  GO:0042981  GO:0046668  GO:0035071  GO:0007291 |
|  | *CG15287-PA* | 2L | 7.42E-08 | unknown |  | unknown |  | unknown |  |
| ACXC | *CG5983-PA* | 2L | 8.35E-08 |  |  | adenylate cyclase activity,  guanylate cyclase activity | GO:0004016; GO:0004383 | G-protein coupled receptor protein signaling pathway,  cyclic nucleotide metabolism,  intracellular signaling cascade | GO:0007186  GO:0009187  GO:0007242 |
| Smg1 | ***CG32743-PA*** | X | 8.71E-08 |  |  | ATP binding,  receptor signaling protein serine/threonine kinase activity,  protein kinase activity | GO:0005524 GO:0004702  GO:0004672 | intracellular signaling cascade,  mRNA catabolism  nonsense-mediated decay,  phosphorylation,  protein amino acid phosphorylation | GO:0007242 GO:0000184  GO:0016310  GO:0006468 |
|  | ***CG33287-PA*** | 3L | 1.04E-07 | unknown |  | unknown |  | unknown |  |
| shtd | ***CG9198-PA*** | X | 1.36E-07 | anaphase-promoting complex | GO:0005680 | ligase activity | GO:0016874 | cell cycle,  development,  meiosis,  regulation of progression through cell cycle | GO:0007049 GO:0007275 GO:0007126 GO:0000074 |

Table S12 Unpolarized cont.

| Gene | CG | ARM | pval | Cellular Component | | Molecular Function | | Biological Process | |
| --- | --- | --- | --- | --- | --- | --- | --- | --- | --- |
| Dhc16F | CG7092-PA | X | 1.88E-07 | axonemal dynein complex, microtubule associated complex | GO:0005858  GO:0005875 | DNA binding,  structural constituent of cytoskeleton, ATPase activity (coupled),  motor activity,  microtubule motor activity,  calcium ion binding  nucleotide binding | GO:0003677 GO:0005200  GO:0042623 GO:0003774 GO:0003777  GO:0005509 GO:0000166 | cell motility,  microtubule-based movement, regulation of transcription (DNA-dependent),  two-component signal transduction system (phosphorelay) | GO:0006928 GO:0007018  GO:0006355  GO:0000160 |
|  | ***CG31320-PA*** | 3R | 4.33E-07 | unknown |  | unknown |  | unknown |  |
|  | *CG14005-PA* | 2L | 7.23E-07 | unknown |  | unknown |  | unknown |  |
| lectin24A | *CG3410-PA* | 2L | 8.48E-07 |  |  | galactose binding | GO:0005534 | spermatogenesis | GO:0007283 |
|  | *CG18600-PA* | 3R | 9.69E-07 | unknown |  | unknown |  | unknown |  |
|  | *CG30020-PA* | 2R | 1.55E-06 | nucleus | GO:0005634 | nucleic acid binding,  zinc ion binding | GO:0003676 GO:0008270 |  |  |
|  | ***CG5096-PA*** | 2L | 1.74E-06 |  |  | receptor activity | GO:0004872 | cell adhesion,  development,  transmission of nerve impulse | GO:0007155 GO:0007275 GO:0019226 |
|  | ***CG6525-PA*** | 3R | 1.77E-06 | nucleus | GO:0005634 | transcription factor activity | GO:0003700 | regulation of transcription,  regulation of transcription (DNA-dependent) | GO:0045449  GO:0006355 |
|  | *CG31091-PA* | 3R | 1.84E-06 |  |  | triacylglycerol lipase activity | GO:0004806 | lipid metabolism | GO:0006629 |
|  | ***CG10805-PA*** | 2L | 1.95E-06 | unknown |  | unknown |  | unknown |  |
|  | *CG33791-PB* | 3L | 3.64E-06 | oxoglutarate dehydrogenase complex | GO:0009353 | oxoglutarate dehydrogenase (succinyl-transferring) activity | GO:0004591 | tricarboxylic acid cycle | GO:0006099 |
