# Supplementary material for: Population Genomics: Whole-Genome Analysis of Polymorphism and Divergence in Drosophila simulans
Source: PLoS Biol. 2007 Nov 6;5(11):e310. doi: 10.1371/journal.pbio.0050310 (PMC2062478; doi:10.1371/journal.pbio.0050310)
Supplement: Supplementary file 19 [file pbio.0050310.st013.doc]

Table S13. Genes showing excess protein polymorphism (p ≤ 0.01) in polarized MK tests

| Gene | Name | NS P | NS F | S P | S F | FET | Cellular Component | Molecular Function | Biological Process |
| --- | --- | --- | --- | --- | --- | --- | --- | --- | --- |
| CG31414 |  | 5 | 1 | 1 | 16 | 0.0010 | lysosome; GO:0005764  membrane; GO:0016020 | transporter activity; GO:0005215  hydrolase activity, hydrolyzing N-glycosyl compounds; GO:0016799  glucosidase activity; GO:0015926  glucosylceramidase activity; GO:0004348 | lipid metabolism; GO:0006629  lysosome organization and biogenesis; GO:0007040  sphingolipid metabolism; GO:0006665  glucosidase activity; GO:0015926 |
| CG9720 | *PH4αNE2* | 14 | 1 | 14 | 13 | 0.0071 | procollagen-proline, 2-oxoglutarate-4-dioxygenase complex; GO:0016222 | procollagen-proline 4-dioxygenase activity; GO:0004656 | peptidyl-proline hydroxylation to 4-hydroxy-L-proline; GO:0018401 |
| CG32602 |  | 10 | 5 | 4 | 18 | 0.0052 |  | receptor activity; GO:0004872 | cell adhesion; GO:0007155  defense response; GO:0006952 |
| CG7158 |  | 12 | 6 | 14 | 49 | 0.0010 |  |  |  |
| CG8403 | *SP2353* | 22 | 3 | 29 | 21 | 0.0093 |  | structural molecule activity; GO:0005198  receptor activity; GO:0004872 | cell adhesion; GO:0007155  cell communication; GO:0007154  cell-cell adhesion; GO:0016337  cell-matrix adhesion; GO:0007160  signal transduction; GO:0007165 |

NS P, NS F, S P, S F, FET are nonsynonymous polymorphisms, nonsynonymous fixations, synonymous polymorphisms, synonymous fixations and Fisher’s Exact test p value.
