# Supplementary material for: Population Genomics: Whole-Genome Analysis of Polymorphism and Divergence in Drosophila simulans
Source: PLoS Biol. 2007 Nov 6;5(11):e310. doi: 10.1371/journal.pbio.0050310 (PMC2062478; doi:10.1371/journal.pbio.0050310)
Supplement: Supplementary file 20 [file pbio.0050310.st014.doc]

Table S14. Gene Ontology terms associated with the top 20 genes with the smallest polarized MK test p-values.

| Gene | CG | ARM | pval | Cellular Component | | Molecular Function | | Biological Process | |
| --- | --- | --- | --- | --- | --- | --- | --- | --- | --- |
|  | CG10805-PA | 2L | 7.69E-07 | unknown |  | unknown |  | unknown |  |
| **trol** | ***CG33950-PF*** | X | 1.25E-06 | basement membrane,  extracellular matrix | GO:0005604  GO:0005578 | DNA binding,  structural molecule activity | GO:0003677 GO:0005198 | DNA methylation,  cell-cell adhesion,  cell-matrix adhesion,  neuroblast division,  signal transduction | GO:0006306 GO:0016337 GO:0007160 GO:0045034 GO:0007165 |
| **shtd** | ***CG9198-PA*** | X | 2.31E-06 | anaphase-promoting complex | GO:0005680 | ligase activity | GO:0016874 | cell cycle,  development,  meiosis,  regulation of progression through cell cycle | GO:0007049 GO:0007275 GO:0007126 GO:0000074 |
| **Pdk2** | ***CG6504-PA*** | 2L | 2.55E-06 | membrane | GO:0016020 | cation channel activity,  calcium ion binding | GO:0005261  GO:0005509 | cation transport,  cell homeostasis,  development,  ion transport,  larval feeding behavior,  smooth muscle contraction,  sperm competition,  sperm motility | GO:0006812  GO:0019725  GO:0007275  GO:0006811  GO:0030536  GO:0006939  GO:0046692  GO:0030317 |
| **Smg1** | ***CG32743-PA*** | X | 2.58E-06 |  |  | ATP binding,  receptor signaling protein serine/threonine kinase activity,  protein kinase activity | GO:0005524 GO:0004702  GO:0004672 | intracellular signaling cascade,  mRNA catabolism  nonsense-mediated decay,  phosphorylation,  protein amino acid phosphorylation | GO:0007242 GO:0000184  GO:0016310  GO:0006468 |
| Gl | *CG9206-PA* | 3L | 4.71E-06 | Cytoplasm, dynactin complex | GO:0005737  GO:0005869 | structural constituent of cytoskeleton, microtubule motor activity,  dynein binding; | GO:0005200  GO:0003777 GO:0045502 | cell fate determination,  eye photoreceptor cell differentiation,  eye photoreceptor development, intracellular protein transport, microtubule-based movement, oogenesis,  photoreceptor cell differentiation,  retrograde axon cargo transport,  rhabdomere development, second mitotic wave,  vesicle-mediated transport | GO:0001709  GO:0001754  GO:0042051  GO:0006886  GO:0007018  GO:0009993 GO:0007467  GO:0008090  GO:0042052 GO:0016330 GO:0016192 |
|  | ***CG31320-PA*** | 3R | 5.11E-06 | unknown |  | unknown |  | unknown |  |
|  | ***CG6525-PA*** | 3R | 9.25E-06 | nucleus | GO:0005634 | transcription factor activity | GO:0003700 | regulation of transcription,  regulation of transcription (DNA-dependent) | GO:0045449  GO:0006355 |
| Mes-4 | *CG4976-PA* | 3R | 1.36E-05 | nuclear chromosome, ubiquitin ligase complex | GO:0000228 GO:0000151 | ubiquitin-protein ligase activity,  transcription cofactor activity,  histone methyltransferase activity  zinc ion binding,  DNA binding,  transcription factor binding | GO:0004842 GO:0003712 GO:0042054 GO:0008270 GO:0003677 GO:0008134 | gene silencing,  germ-line sex determination,  protein ubiquitination, regulation of transcription from RNA pol II promoter; | GO:0016458 GO:0018992 GO:0016567 GO:0006357 |
| Upf2 | *CG2253-PA* | X | 2.07E-05 |  |  | mRNA binding | GO:0003729 | protein biosynthesis | GO:0006412 |

| Gene | CG | ARM | pval | Cellular Component | | Molecular Function | | Biological Process | |
| --- | --- | --- | --- | --- | --- | --- | --- | --- | --- |
|  | CG1571-PA | X | 2.81E-05 | cytoplasmic dynein complex, dynein complex,  microtubule assoc. complex | GO:0005868  GO:0030286  GO:0005875 | structural constituent of cytoskeleton, ATPase activity (coupled),  motor activity | GO:0005200  GO:0042623 GO:0003774 | microtubule-based movement | GO:0007018 |
|  | ***CG33287-PA*** | 3L | 2.90E-05 | unknown |  | unknown |  | unknown |  |
|  | ***CG5096-PA*** | 2L | 3.06E-05 |  |  | receptor activity | GO:0004872 | cell adhesion, development, transmission of nerve impulse | GO:0007155 GO:0007275 GO:0019226 |
| Gap1 | *CG6721-PA* | 3L | 3.36E-05 | plasma membrane | GO:0005886 | Ras GTPase activator activity | GO:0005099 | MAPKKK cascade,  Ras protein signal transduction,  determination of anterior/posterior axis (embryo),  female meiosis chromosome segregation,  mitosis,  negative regulation of Ras protein signal transduction,  sister chromatid cohesion,  torso signaling pathway | GO:0000165 GO:0007265  GO:0008595  GO:0016321  GO:0007067 GO:0046580  GO:0007062  GO:0008293 |
|  | *CG9346-PA* | 2R | 7.50E-05 |  |  | mRNA binding | GO:0003729 | RNA processing | GO:0006396 |
|  | *CG8443-PA* | 2R | 8.43E-05 | eukaryotic translation initiation factor 3 complex | GO:0005852 | translation initiation factor activity | GO:0003743 | regulation of translation,  translational initiation; | GO:0006445 GO:0006413 |
|  | *CG14339-PA* | 2L | 8.85E-05 | unknown |  | unknown |  | unknown |  |
|  | *CG8092-PA* | 2R | 1.00E-04 | nucleus | GO:0005634 | DNA binding,  nucleic acid binding,  zinc ion binding | GO:0003677 GO:0003676 GO:0008270 | regulation of transcription (DNA-dependent) | GO:0006355 |
| Pvr | *CG8222-PA* | 2L | 1.04E-04 | plasma membrane | GO:0005886 | ATP binding,  transmembrane receptor protein tyrosine kinase activity,  vascular endothelial growth factor receptor activity,  protein-tyrosine kinase activity,  protein binding,  platelet-derived growth factor receptor activity | GO:0005524 GO:0004714  GO:0005021  GO:0004713 GO:0005515 GO:0005017 | actin cytoskeleton organization and biogenesis,  actin filament organization,  border follicle cell migration,  cell projection biogenesis,  cell proliferation,  ectoderm development,  hemocyte differentiation,  hemocyte migration,  hemocyte proliferation,  hemopoiesis,  protein amino acid phosphorylation,  regulation of cell shape,  regulation of hemocyte differentiation, transmembrane receptor protein tyrosine kinase signaling pathway,  ventral cord development | GO:0030036  GO:0007015 GO:0007298 GO:0030031 GO:0008283 GO:0007398 GO:0042386 GO:0035099 GO:0035172 GO:0030097 GO:0006468  GO:0008360 GO:0045610  GO:0007169  GO:0007419 |
|  | *CG9121-PA* | 2L | 1.09E-04 |  |  | structural constituent of cytoskeleton,  nucleotidyltransferase activity | GO:0005200  GO:0016779 | DNA replication,  cytoskeletal anchoring,  protein amino acid ADP-ribosylation,  transcription (RNA-dependent) | GO:0006260 GO:0007016 GO:0006471  GO:0006410 |
