# Supplementary material for: Population Genomics: Whole-Genome Analysis of Polymorphism and Divergence in Drosophila simulans
Source: PLoS Biol. 2007 Nov 6;5(11):e310. doi: 10.1371/journal.pbio.0050310 (PMC2062478; doi:10.1371/journal.pbio.0050310)
Supplement: Supplementary file 21 [file pbio.0050310.st015.doc]

Table S15. Gene Ontology categories enriched among genes with significant (p ≤ 0.05) unpolarized MK tests.

| GO ID | Number of Genes | | Proportion Significant tests | GO Term | Permutation p-value |
| --- | --- | --- | --- | --- | --- |
| Cellular Components | | | |  |  |
| GO:0005694 | 67 | 0.40 | | chromosome | 0.0003 |
| GO:0005856 | 103 | 0.31 | | cytoskeleton | 0.0157 |
| GO:0005871 | 20 | 0.45 | | kinesin complex | 0.0063 |
| GO:0005875 | 32 | 0.37 | | microtubule associated complex | 0.0146 |
| GO:0005635 | 36 | 0.50 | | nuclear envelope | 0.0001 |
| GO:0005643 | 24 | 0.58 | | nuclear pore | 0.0001 |
| GO:0005654 | 68 | 0.34 | | nucleoplasm | 0.0100 |
| Molecular Functions | | | |  |  |
| GO:0042623 | 27 | 0.37 | | ATPase activity, coupled | 0.0246 |
| GO:0003682 | 51 | 0.39 | | chromatin binding | 0.0018 |
| GO:0004197 | 36 | 0.39 | | cystein-type endopeptidase activity | 0.0070 |
| GO:0004386 | 34 | 0.35 | | helicase activity | 0.0271 |
| GO:0003777 | 28 | 0.46 | | microtubule motor activity | 0.0015 |
| GO:0003774 | 40 | 0.42 | | motor activity | 0.0012 |
| GO:0005200 | 114 | 0.38 | | structural constituent of cytoskeleton | <0.0001 |
| GO:0005198 | 262 | 0.28 | | structural molecule activity | 0.0129 |
| GO:0005351 | 21 | 0.52 | | sugar porter activity | 0.0006 |
| GO:0003712 | 21 | 0.43 | | transcription cofactor activity | 0.0093 |
| Biological Processes | | | |  |  |
| GO:0006865 | 22 | 0.36 | | amino acid transport | 0.0401 |
| GO:0008643 | 27 | 0.41 | | carbohydrate transport | 0.0069 |
| GO:0016043 | 377 | 0.27 | | cell organization & biogenesis | 0.0272 |
| GO:0006333 | 31 | 0.35 | | chromatin assembly or disassembly | 0.0296 |
| GO:0007254 | 20 | 0.45 | | JNK cascade | 0.0055 |
| GO:0007018 | 39 | 0.38 | | microtubule-based movement | 0.0068 |
| GO:0006605 | 56 | 0.36 | | protein targeting | 0.0073 |
| GO:0015031 | 208 | 0.27 | | protein transport | 0.0384 |
| GO:0040029 | 25 | 0.52 | | regulation of gene expression - epigenetic | 0.0002 |
| GO:0000003 | 216 | 0.30 | | reproduction | 0.0051 |
| GO:0007283 | 37 | 0.41 | | spermatogenesis | 0.0040 |
| GO:0006511 | 32 | 0.34 | | ubiquitin-dependent protein catabolism | 0.0389 |
