# Supplementary material for: Population Genomics: Whole-Genome Analysis of Polymorphism and Divergence in Drosophila simulans
Source: PLoS Biol. 2007 Nov 6;5(11):e310. doi: 10.1371/journal.pbio.0050310 (PMC2062478; doi:10.1371/journal.pbio.0050310)
Supplement: Supplementary file 22 [file pbio.0050310.st016.doc]

Table S16. Gene Ontology categories enriched among genes with significant (p ≤ 0.05) polarized MK tests.

| GO ID | Number of Genes | Prop. Sig. tests | | GO Term | Permutation p-value |
| --- | --- | --- | --- | --- | --- |
| Cellular Components | | | |  |  |
| GO:0000775 | 6 | 0.33 | | chromosome, pericentric region | 0.0500 |
| GO:0005856 | 47 | 0.26 | | cytoskeleton | 0.0233 |
| GO:0000792 | 5 | 0.40 | | heterochromatin | 0.0253 |
| GO:0005622 | 485 | 0.18 | | intracellular | 0.0379 |
| GO:0005635 | 23 | 0.30 | | nuclear envelope | 0.0186 |
| GO:0005643 | 18 | 0.39 | | nuclear pore | 0.0027 |
| GO:0016469 | 8 | 0.38 | | proton-transporting two-sector ATPase complex | 0.0238 |
| GO:0000151 | 28 | 0.29 | | ubiquitin ligase complex | 0.0191 |
| Molecular Functions | | |  | |  |
| GO:0003779 | 21 | 0.33 | | actin binding | 0.0097 |
| GO:0004016 | 5 | 0.60 | | adenylate cyclase activity | 0.0018 |
| GO:0008324 | 12 | 0.33 | | cation transporter activity | 0.0254 |
| GO:0008092 | 46 | 0.28 | | cytoskeletal protein binding | 0.0087 |
| GO:0003684 | 9 | 0.33 | | damaged DNA binding | 0.0354 |
| GO:0005355 | 6 | 0.33 | | glucose transporter activity | 0.0500 |
| GO:0004383 | 9 | 0.33 | | guanylate cyclase activity | 0.0354 |
| GO:0046933 | 9 | 0.33 | | hydrogen-transporting ATP synthase activity, rotational mechanism | 0.0354 |
| GO:0046961 | 9 | 0.33 | | hydrogen-transporting ATPase activity, rotational mechanism | 0.0354 |
| GO:0004194 | 5 | 0.40 | | pepsin A activity | 0.0253 |
| GO:0005200 | 53 | 0.30 | | structural constituent of cytoskeleton | 0.0015 |
| GO:0005198 | 98 | 0.21 | | structural molecule activity | 0.0400 |
| GO:0016757 | 5 | 0.40 | | transferase activity, transferring glycosyl groups | 0.0253 |
| GO:0003743 | 11 | 0.36 | | translation initiation factor activity | 0.0191 |
| GO:0005215 | 181 | 0.21 | | transporter activity | 0.0163 |
| GO:0004842 | 38 | 0.26 | | ubiquitin-protein ligase activity | 0.0240 |
| GO:0004843 | 8 | 0.38 | | ubiquitin-specific protease activity | 0.0238 |
| Biological Processes | | | |  |  |
| GO:0008652 | 8 | 0.38 | | amino acid biosynthesis | 0.0238 |
| GO:0006820 | 8 | 0.50 | | anion transport | 0.0031 |
| GO:0015986 | 9 | 0.33 | | ATP synthesis coupled proton transport | 0.0354 |
| GO:0008219 | 47 | 0.23 | | cell death | 0.0495 |
| GO:0016049 | 6 | 0.50 | | cell growth | 0.0066 |
| GO:0006928 | 9 | 0.44 | | cell motility | 0.0060 |
| GO:0016043 | 185 | 0.22 | | cell organization and biogenesis | 0.0045 |
| GO:0008283 | 214 | 0.20 | | cell proliferation | 0.0220 |
| GO:0008151 | 522 | 0.18 | | cellular physiological process | 0.0235 |
| GO:0009187 | 7 | 0.43 | | cyclic nucleotide metabolism | 0.0127 |
| GO:0007282 | 7 | 0.43 | | cystoblast division | 0.0127 |
| GO:0007028 | 122 | 0.21 | | cytoplasm organization and biogenesis | 0.0279 |
|  |  |  | |  |  |
| Table S16 cont. |  |  | |  |  |
| GO ID | Number of Genes | Prop. Sig. tests | | GO Term | Permutation p-value |
| GO:0007010 | 104 | 0.23 | | cytoskeleton organization and biogenesis | 0.0138 |
| GO:0008595 | 6 | 0.33 | | determination of anterior/posterior axis, embryo | 0.0500 |
| GO:0007275 | 347 | 0.20 | | development | 0.0045 |
| GO:0006325 | 6 | 0.33 | | establishment and/or maintenance of chromatin architecture | 0.0500 |
| GO:0042051 | 5 | 0.60 | | eye photoreceptor development (sensu Endopterygota) | 0.0018 |
| GO:0016321 | 7 | 0.43 | | female meiosis chromosome segregation | 0.0127 |
| GO:0016458 | 5 | 0.40 | | gene silencing | 0.0253 |
| GO:0007281 | 5 | 0.40 | | germ cell development | 0.0253 |
| GO:0008354 | 6 | 0.33 | | germ cell migration | 0.0500 |
| GO:0006096 | 5 | 0.40 | | glycolysis | 0.0253 |
| GO:0040007 | 13 | 0.31 | | growth | 0.0361 |
| GO:0007444 | 9 | 0.33 | | imaginal disc development | 0.0354 |
| GO:0002168 | 5 | 0.40 | | larval development (sensu Insecta) | 0.0253 |
| GO:0006869 | 8 | 0.38 | | lipid transport | 0.0238 |
| GO:0007018 | 17 | 0.35 | | microtubule-based movement | 0.0085 |
| GO:0006298 | 5 | 0.40 | | mismatch repair | 0.0253 |
| GO:0006397 | 5 | 0.40 | | mRNA processing | 0.0253 |
| GO:0009299 | 5 | 0.40 | | mRNA transcription | 0.0253 |
| GO:0016481 | 6 | 0.33 | | negative regulation of transcription | 0.0500 |
| GO:0006996 | 117 | 0.22 | | organelle organization and biogenesis | 0.0178 |
| GO:0006817 | 6 | 0.33 | | phosphate transport | 0.0500 |
| GO:0016310 | 8 | 0.50 | | phosphorylation | 0.0031 |
| GO:0007582 | 1211 | 0.17 | | physiological process | 0.0037 |
| GO:0040018 | 5 | 0.60 | | positive regulation of body size | 0.0018 |
| GO:0030307 | 5 | 0.40 | | positive regulation of cell growth | 0.0253 |
| GO:0045793 | 5 | 0.60 | | positive regulation of cell size | 0.0018 |
| GO:0016579 | 7 | 0.43 | | protein deubiquitination | 0.0127 |
| GO:0000059 | 6 | 0.33 | | protein import into nucleus, docking | 0.0500 |
| GO:0016567 | 28 | 0.29 | | protein ubiquitination | 0.0191 |
| GO:0008361 | 5 | 0.40 | | regulation of cell size | 0.0253 |
| GO:0040029 | 14 | 0.36 | | regulation of gene expression, epigenetic | 0.0149 |
| GO:0007088 | 9 | 0.33 | | regulation of mitosis | 0.0354 |
| GO:0046620 | 6 | 0.33 | | regulation of organ size | 0.0500 |
| GO:0006445 | 14 | 0.29 | | regulation of translation | 0.0530 |
| GO:0000003 | 104 | 0.22 | | reproduction | 0.0280 |
| GO:0006413 | 9 | 0.44 | | translational initiation | 0.0060 |
| GO:0006099 | 5 | 0.40 | | tricarboxylic acid cycle | 0.0253 |
| GO:0006511 | 12 | 0.33 | | ubiquitin-dependent protein catabolism | 0.0254 |
