# Supplementary material for: Population Genomics: Whole-Genome Analysis of Polymorphism and Divergence in Drosophila simulans
Source: PLoS Biol. 2007 Nov 6;5(11):e310. doi: 10.1371/journal.pbio.0050310 (PMC2062478; doi:10.1371/journal.pbio.0050310)
Supplement: Supplementary file 23 [file pbio.0050310.st017.doc]

# Table S17. Tissue-specific or developmental stage-specific expression patterns enriched with significant (p ≤ 0.05) MK tests

|  | Polarized MK tests | | |  | Unpolarized MK tests | | |
| --- | --- | --- | --- | --- | --- | --- | --- |
|  | #genes | Prop. Sig. | p-value |  | #genes | Prop. Sig. | p-value |
| Male-biased | 114 | 0.1842 | 0.0905 |  | 265 | 0.2415 | 0.0103 |
| Testes-expressed | 347 | 0.1671 | 0.0871 |  | 807 | 0.2491 | <0.0001 |
| Female-biased | 84 | 0.1786 | 0.1460 |  | 162 | 0.1605 | 0.8005 |
| Female mating responsive | 89 | 0.1236 | 0.6506 |  | 214 | 0.1636 | 0.8141 |
| Spermatheca-expressed | 75 | 0.1600 | 0.2764 |  | 179 | 0.1732 | 0.6722 |
| Ovary-expressed | 132 | 0.1439 | 0.4528 |  | 248 | 0.1653 | 0.8137 |
| Embryo-biased | 136 | 0.1471 | 0.4114 |  | 280 | 0.2643 | 0.0002 |
| Larvae-biased | 46 | 0.1739 | 0.2152 |  | 132 | 0.2879 | 0.0021 |
| Pupae-biased | 38 | 0.0789 | 0.8164 |  | 95 | 0.1158 | 0.9653 |
| Head-expressed | 433 | 0.1247 | 0.8861 |  | 1245 | 0.1671 | 0.9862 |

See Methods for definitions of expression categories.
