# Supplementary material for: Population Genomics: Whole-Genome Analysis of Polymorphism and Divergence in Drosophila simulans
Source: PLoS Biol. 2007 Nov 6;5(11):e310. doi: 10.1371/journal.pbio.0050310 (PMC2062478; doi:10.1371/journal.pbio.0050310)
Supplement: Supplementary file 24 [file pbio.0050310.st018.doc]

Table S18. Genes having the greatest relative rate test χ^2^ test statistics for dN in the *D. simulans* lineage

Gene	χ^2^	sim dN	mel dN	Name	Cellular Component	Molecular Function	Biological Process

*CG3312-PA*	188.8	0.0918	0.0281	*Rnp4F*	intracellular; GO:0005622	mRNA binding; GO:0003729	RNA processing; GO:0006396

*CG2791-PA*	100.8	0.0515	0.0065

*CG17523-PA*	81.9	0.0764	0.0175	*GstE2*	glutathione transferase activity; GO:0004364	defense response; GO:0006952

response to toxin; GO:0009636

*CG3491-PA*	81.9	0.05	0.0176	membrane; GO:0016020	DNA binding; GO:0003677	regulation of transcription, DNA-dependent; GO:0006355

nucleus; GO:0005634	nucleic acid binding; GO:0003676	transport; GO:0006810

ATP binding; GO:0005524

helicase activity; GO:0004386

transporter activity; GO:0005215

*CG12717-PA*	64.8	0.1051	0.0407

*CG1826-PA*	56.4	0.0405	0.0104	actin binding; GO:0003779

structural constituent of cytoskeleton; GO:0005200

*CG31219-PA*	52.9	0.1274	0.0567	trypsin activity; GO:0004295

chymotrypsin activity; GO:0004263

monophenol monooxygenase activator activity; GO:0008439	proteolysis; GO:0006508

*CG6352-PA*	52.7	0.1175	0.0334	*OdsH*	nucleus; GO:0005634	transcription factor activity; GO:0003700	regulation of transcription; GO:0045449

regulation of transcription from RNA polymerase II promoter; GO:0006357

*CG8834-PA*	52.1	0.0224	0.002	ligase activity; GO:0016874	metabolism; GO:0008152

4-coumarate-CoA ligase activity

*CG15523-PB*	51.8	0.0125	0.0064

*CG8870-PA*	51.5	0.1022	0.0327	nucleus; GO:0005634	DNA binding; GO:0003677	proteolysis; GO:0006508

trypsin activity; GO:0004295	regulation of transcription, DNA-dependent; GO:0006355

chymotrypsin activity; GO:0004263

monophenol monooxygenase activator activity; GO:0008439

transcription factor activity; GO:0003700

*CG13838-PA*	48.8	0.1169	0.0062

*CG4104-PA*	47.0	0.0077	0.0006	*Tps1*	alpha,alpha-trehalose-phosphate synthase (UDP-forming)	disaccharide metabolism; GO:0005984

activity; GO:0003825	trehalose biosynthesis; GO:0005992

trehalose-phosphatase activity; GO:0004805

*CG3730-PA*	46.1	0.0302	0.0111	*csul*	cytoplasm; GO:0005737	protein methyltransferase activity; GO:0008276	MAPKKK cascade; GO:0000165

receptor binding; GO:0005102	mitosis; GO:0007067

pole plasm assembly; GO:0007315

*CG12317-PA*	44.9	0.0226	0.0028	*JhI-21*	membrane; GO:0016020	amino acid transporter activity; GO:0015171	amino acid metabolism; GO:0006520

L-amino acid transporter activity; GO:0015179	amino acid transport; GO:0006865

amino acid transporter activity; GO:0015171

amino acid-polyamine transporter activity; GO:0005279

*CG12598-PA*	42.9	0.0127	0	*Adar*	intracellular; GO:0005622	double-stranded RNA binding; GO:0003725	RNA editing; GO:0016547

double-stranded RNA adenosine deaminase activity	adenosine to inosine editing; GO:0006382

adult behavior; GO:0030534

adult locomotory behavior; GO:0008344

mRNA editing; GO:0006381

mRNA processing; GO:0006397

regulation of membrane potential; GO:0042391

response to heat; GO:0009408

response to hypoxia; GO:0001666

*CG32188*	39.7	0.1271	0.0253

*CG10533*	38.9	0.1525	0.0891	*Lcp65Af*	structural constituent of larval cuticle (sensu

Insecta); GO:0008010

*CG31775*	35.4	0.0958	0.018

*CG12590-PA*	33.2	0.0851	0.0198

*CG32743-PA*	32.0	0.0252	0.0186	*Smg1*	ATP binding; GO:0005524	intracellular signaling cascade; GO:0007242

receptor signaling protein serine/threonine	mRNA catabolism, nonsense-mediated decay; GO:0000184

kinase activity; GO:0004702	phosphorylation; GO:0016310

protein kinase activity; GO:0004672	protein amino acid phosphorylation; GO:0006468

*CG6819*	31.3	0.0239	0.0096	*mbo*	nuclear membrane; GO:0005635	transporter activity; GO:0005215	antimicrobial humoral response (sensu Protostomia); GO:0006960

nuclear pore; GO:0005643	protein binding; GO:0005515	immune response; GO:0006955

negative regulation of protein export from nucleus; GO:0046826

protein import into nucleus; GO:0006606

protein targeting; GO:0006605

tracheal branch fusion; GO:0035147

tracheal system development (sensu Insecta); GO:0007424

*CG10478-PA*	30.7	0.0611	0.0198	nuclear pore; GO:0005643	protein carrier activity; GO:0008320	protein import into nucleus; GO:0006606

*CG7046*	30.4	0.1072	0.0373	chromatin; GO:0000785	DNA binding; GO:0003677	regulation of transcription, DNA-dependent; GO:0006355

nucleus; GO:0005634

*CG5999*	29.3	0.0296	0.0074	glucuronosyltransferase activity; GO:0015020	defense response; GO:0006952
