# Supplementary material for: Population Genomics: Whole-Genome Analysis of Polymorphism and Divergence in Drosophila simulans
Source: PLoS Biol. 2007 Nov 6;5(11):e310. doi: 10.1371/journal.pbio.0050310 (PMC2062478; doi:10.1371/journal.pbio.0050310)
Supplement: Supplementary file 25 [file pbio.0050310.st019.doc]

# Table S19. Genes having the greatest relative rate test χ^2^ test statistics for dN in the *D. melanogaster* lineage

Gene	χ^2^	mel dN	sim dN	Name	Cellular Component	Molecular Function	Biological Process

*CG3415*	41.4	0.0148	0.0014	estradiol 17-beta-dehydrogenase activity; GO:0004303	fatty acid biosynthesis; GO:0006633

cysteine-type endopeptidase activity; GO:0004197	proteolysis; GO:0006508

steroid metabolism; GO:0008202

*CG18096*	39.2	0.0482	0.0323	*Tep1*	endopeptidase inhibitor activity; GO:0004866	antibacterial humoral response (sensu Protostomia); GO:0006961

*CG33523*	37.6	0.0277	0.0078

*CG5270*	34.5	0.0119	0.0051

*CG7526*	34.2	0.0291	0.0119	structural molecule activity; GO:0005198	cell communication; GO:0007154

receptor activity; GO:0004872	cell surface receptor linked signal transduction GO:0007166

calcium ion binding; GO:0005509	protein amino acid phosphorylation; GO:0006468

transmembrane receptor protein tyrosine kinase activity; GO:0004714	protein metabolism; GO:0019538

signal transduction; GO:0007165

protein modification; GO:0006464

transmembrane receptor protein tyrosine kinase

signaling pathway; GO:0007169

*CG3508*	33.9	0.0198	0.0034

*CG3086*	33.6	0.0234	0.0051	*MAPk-Ak2*	ATP binding; GO:0005524	MAPKKK cascade; GO:0000165

receptor signaling protein serine/threonine kinase	cell adhesion; GO:0007155

activity; GO:0004702	defense response; GO:0006952

protein serine/threonine kinase activity; GO:0004674	protein amino acid phosphorylation; GO:0006468

regulation of cell shape; GO:0008360

response to stress; GO:0006950

*CG13316*	33.2	0.0295	0.0118	*Mnt*	nucleus; GO:0005634	DNA binding; GO:0003677	regulation of transcription; GO:0045449

RNA polymerase II transcription factor activity;

GO:0003702

transcription factor activity; GO:0003700

*CG2977*	32.0	0.0177	0.0017	*inx7*	gap junction; GO:0005921	innexin channel activity; GO:0015286	signal transduction; GO:0007165

integral to membrane; GO:0016021	gap-junction forming channel activity; GO:0005243

*CG2254*	30.9	0.0247	0.0084	oxidoreductase activity; GO:0016491	metabolism; GO:0008152

visual perception; GO:0007601

*CG1747*	29.8	0.0200	0.0022	*Sk1*	diacylglycerol kinase activity; GO:0004143	anti-apoptosis; GO:0006916

D-erythro-sphingosine kinase activity; GO:0017050	phosphorylation; GO:0016310

carbohydrate kinase activity; GO:0019200	protein kinase C activation; GO:0007205

signal transduction; GO:0007165

sphingolipid metabolism; GO:0006665

sphingosine metabolism; GO:0006670

*CG12139*	29.5	0.0034	0.0004	membrane; GO:0016020	low-density lipoprotein receptor activity; GO:0005041	cholesterol metabolism; GO:0008203

calcium ion binding; GO:0005509	intracellular protein transport; GO:0006886

receptor mediated endocytosis; GO:0006898

*CG32223*	25.8	0.0262	0.0159

*CG12016*	24.2	0.0173	0.0014

*CG3540*	24.0	0.0221	0.0077	*Cyp4d14*	membrane; GO:0016020	electron transporter activity; GO:0005489	electron transport; GO:0006118

microsome; GO:0005792	oxidoreductase activity; GO:0016491

monooxygenase activity; GO:0004497

*CG3842*	23.3	0.0232	0.0051	oxidoreductase activity, acting on CH-OH group of	metabolism; GO:0008152

donors; GO:0016614

*CG9630*	23.0	0.0086	0.0007	nucleic acid binding; GO:0003676	nucleobase, nucleoside, nucleotide and nucleic acid

ATP binding; GO:0005524	metabolism; GO:0006139

nucleic acid binding; GO:0003676

ATP-dependent RNA helicase activity; GO:0004004

*CG5866*	22.9	0.0673	0.0046

*CG6833*	22.2	0.0375	0.0251	intracellular; GO:0005622	nucleic acid binding; GO:0003676	RNA catabolism; GO:0006401

nuclease activity; GO:0004518	cell cycle; GO:0007049

exoribonuclease activity; GO:0004532	mitosis; GO:0007067

nucleobase, nucleoside, nucleotide and nucleic acid

metabolism; GO:0006139

*CG8869*	22.2	0.0243	0.0033	*Jon25Bii*	serine-type endopeptidase activity; GO:0004252	proteolysis; GO:0006508

chymotrypsin activity; GO:0004263

*CG12206*	21.3	0.0210	0.0071

*CG7619*	21.3	0.0099	0.0000	*Pros54*	endopeptidase activity; GO:0004175	chromosome segregation; GO:0007059

protein metabolism; GO:0019538

proteolysis; GO:0006508

ubiquitin-dependent protein catabolism; GO:0006511

*CG14517*	20.0	0.0151	0.0016	*B4GalNAcTB*	acetylgalactosaminyltransferase activity; GO:0008376	N-acetylglucosamine metabolism; GO:0006044

polysaccharide metabolism; GO:0005976

protein amino acid glycosylation; GO:0006486

*CG5059*	20.0	0.0240	0.0025

*CG4594*	19.9	0.0273	0.0071	hydro-lyase activity; GO:0016836	fatty acid beta-oxidation; GO:0006635
