# Supplementary material for: Population Genomics: Whole-Genome Analysis of Polymorphism and Divergence in Drosophila simulans
Source: PLoS Biol. 2007 Nov 6;5(11):e310. doi: 10.1371/journal.pbio.0050310 (PMC2062478; doi:10.1371/journal.pbio.0050310)
Supplement: Supplementary file 26 [file pbio.0050310.st020.doc]

Table S20. Gene Ontology categories enriched among proteins showing accelerated protein evolution (χ^2^ test p value ≤ 0.01) in the *D. simulans* lineage.

| GO ID | Number of Genes | Proportion p ≤ 0.01 | GO Term | Permutation p-value |
| --- | --- | --- | --- | --- |
| Cellular Components | | |  |  |
| GO:0045179 | 9 | 0.11 | apical cortex | 0.0145 |
| GO:0016324 | 12 | 0.08 | apical plasma membrane | 0.0266 |
| GO:0005604 | 6 | 0.17 | basement membrane | 0.0066 |
| GO:0008372 | 507 | 0.03 | cellular component unknown | 0.0372 |
| GO:0030125 | 6 | 0.17 | clathrin vesicle coat | 0.0066 |
| GO:0030135 | 8 | 0.13 | coated vesicle | 0.0112 |
| GO:0000177 | 6 | 0.17 | cytoplasmic exosome (RNase complex) | 0.0066 |
| GO:0005869 | 8 | 0.13 | dynactin complex | 0.0112 |
| GO:0005789 | 13 | 0.08 | endoplasmic reticulum membrane | 0.0316 |
| GO:0016281 | 8 | 0.25 | eukaryotic translation initiation factor 4F complex | 0.0004 |
| GO:0045169 | 12 | 0.08 | fusome | 0.0266 |
| GO:0000792 | 7 | 0.14 | heterochromatin | 0.0093 |
| GO:0005815 | 27 | 0.07 | microtubule organizing center | 0.0208 |
| GO:0031523 | 10 | 0.10 | Myb complex | 0.0193 |
| GO:0005635 | 40 | 0.15 | nuclear envelope | <0.0001 |
| GO:0000176 | 9 | 0.11 | nuclear exosome (RNase complex) | 0.0145 |
| GO:0005643 | 30 | 0.13 | nuclear pore | 0.0005 |
| GO:0005701 | 5 | 0.20 | polytene chromosome chromocenter | 0.0038 |
| GO:0005918 | 8 | 0.13 | septate junction | 0.0112 |
| GO:0000922 | 5 | 0.20 | spindle pole | 0.0038 |
| GO:0035003 | 6 | 0.17 | subapical complex | 0.0066 |
| Molecular Functions | | |  |  |
| GO:0015171 | 8 | 0.25 | amino acid transporter activity | 0.0004 |
| GO:0005279 | 18 | 0.17 | amino acid-polyamine transporter activity | 0.0009 |
| GO:0004812 | 12 | 0.08 | aminoacyl-tRNA ligase activity | 0.0266 |
| GO:0004177 | 5 | 0.20 | aminopeptidase activity | 0.0038 |
| GO:0008026 | 17 | 0.12 | ATP-dependent helicase activity | 0.0058 |
| GO:0004004 | 27 | 0.07 | ATP-dependent RNA helicase activity | 0.0208 |
| GO:0004089 | 9 | 0.11 | carbonate dehydratase activity | 0.0145 |
| GO:0004681 | 7 | 0.14 | casein kinase I activity | 0.0093 |
| GO:0008061 | 33 | 0.06 | chitin binding | 0.0396 |
| GO:0004785 | 5 | 0.20 | copper, zinc superoxide dismutase activity | 0.0038 |
| GO:0004197 | 40 | 0.10 | cysteine-type endopeptidase activity | 0.0018 |
| GO:0003725 | 9 | 0.22 | double-stranded RNA binding | 0.0008 |
| GO:0042708 | 9 | 0.11 | elastase activity | 0.0145 |
| GO:0005355 | 13 | 0.08 | glucose transporter activity | 0.0316 |
| GO:0015926 | 10 | 0.10 | glucosidase activity | 0.0193 |
| GO:0004386 | 32 | 0.09 | helicase activity | 0.0052 |

| GO ID | Number of Genes | Proportion accelerated | GO Term | Permutation p-value |
| --- | --- | --- | --- | --- |
| Molecular Functions Cont. | | |  |  |
| GO:0005316 | 13 | 0.08 | high affinity inorganic phosphate:sodium symporter activity | 0.0316 |
| GO:0042054 | 7 | 0.14 | histone methyltransferase activity | 0.0093 |
| GO:0004178 | 8 | 0.13 | leucyl aminopeptidase activity | 0.0112 |
| GO:0016874 | 44 | 0.07 | ligase activity | 0.0176 |
| GO:0016298 | 15 | 0.07 | lipase activity | 0.0440 |
| GO:0030145 | 6 | 0.17 | manganese ion binding | 0.0066 |
| GO:0046872 | 37 | 0.08 | metal ion binding | 0.0095 |
| GO:0003777 | 31 | 0.06 | microtubule motor activity | 0.0329 |
| GO:0005554 | 473 | 0.04 | molecular function unknown | 0.0228 |
| GO:0015355 | 5 | 0.20 | monocarboxylate porter activity | 0.0038 |
| GO:0008439 | 5 | 0.20 | monophenol monooxygenase activator activity | 0.0038 |
| GO:0003730 | 7 | 0.14 | mRNA 3'-UTR binding | 0.0093 |
| GO:0008080 | 14 | 0.07 | N-acetyltransferase activity | 0.0365 |
| GO:0016779 | 12 | 0.08 | nucleotidyltransferase activity | 0.0266 |
| GO:0008320 | 14 | 0.14 | protein carrier activity | 0.0035 |
| GO:0005097 | 5 | 0.20 | Rab GTPase activator activity | 0.0038 |
| GO:0008536 | 6 | 0.17 | Ran GTPase binding | 0.0066 |
| GO:0000339 | 8 | 0.25 | RNA cap binding | 0.0004 |
| GO:0003724 | 24 | 0.08 | RNA helicase activity | 0.0162 |
| GO:0003697 | 11 | 0.09 | single-stranded DNA binding | 0.0221 |
| GO:0008010 | 31 | 0.06 | structural constituent of larval cuticle (sensu Insecta) | 0.0329 |
| GO:0016490 | 17 | 0.12 | structural constituent of peritrophic membrane (sensu Insecta) | 0.0058 |
| GO:0003714 | 11 | 0.09 | transcription corepressor activity | 0.0221 |
| GO:0008134 | 13 | 0.08 | transcription factor binding | 0.0316 |
| GO:0008135 | 63 | 0.06 | translation factor activity, nucleic acid binding | 0.0147 |
| GO:0003743 | 44 | 0.09 | translation initiation factor activity | 0.0028 |
| GO:0045182 | 66 | 0.06 | translation regulator activity | 0.0156 |
| GO:0004221 | 19 | 0.11 | ubiquitin thiolesterase activity | 0.0070 |
| GO:0004843 | 13 | 0.23 | ubiquitin-specific protease activity | <0.0001 |
| GO:0000175 | 13 | 0.08 | 3'-5'-exoribonuclease activity | 0.0316 |
| Biological Processes | | |  |  |
| GO:0051017 | 5 | 0.20 | actin filament bundle formation | 0.0038 |
| GO:0030037 | 5 | 0.20 | actin filament reorganization during cell cycle | 0.0038 |
| GO:0030534 | 5 | 0.20 | adult behavior | 0.0038 |
| GO:0006865 | 31 | 0.10 | amino acid transport | 0.0053 |
| GO:0006960 | 13 | 0.08 | antimicrobial humoral response (sensu Protostomia) | 0.0316 |
| GO:0045176 | 6 | 0.17 | apical protein localization | 0.0066 |
| GO ID | Number of Genes | Proportion accelerated | GO Term | Permutation p-value |
| Biological Processes Cont. | | |  |  |
| GO:0008356 | 13 | 0.08 | asymmetric cell division | 0.0316 |
| GO:0048102 | 49 | 0.08 | autophagic cell death | 0.0051 |
| GO:0006584 | 8 | 0.13 | catecholamine metabolism | 0.0112 |
| GO:0006030 | 32 | 0.06 | chitin metabolism | 0.0332 |
| GO:0008203 | 13 | 0.08 | cholesterol metabolism | 0.0316 |
| GO:0030261 | 12 | 0.08 | chromosome condensation | 0.0266 |
| GO:0007059 | 35 | 0.06 | chromosome segregation | 0.0446 |
| GO:0007619 | 15 | 0.07 | courtship behavior | 0.0440 |
| GO:0007282 | 9 | 0.11 | cystoblast division | 0.0145 |
| GO:0007016 | 11 | 0.09 | cytoskeletal anchoring | 0.0221 |
| GO:0005984 | 5 | 0.20 | disaccharide metabolism | 0.0038 |
| GO:0042023 | 8 | 0.13 | DNA endoreduplication | 0.0112 |
| GO:0006897 | 32 | 0.06 | endocytosis | 0.0332 |
| GO:0006888 | 15 | 0.07 | ER to Golgi vesicle-mediated transport | 0.0440 |
| GO:0016332 | 5 | 0.20 | establishment and/or maintenance of polarity of embryonic epithelium | 0.0038 |
| GO:0042051 | 13 | 0.08 | eye photoreceptor development (sensu Endopterygota) | 0.0316 |
| GO:0016458 | 12 | 0.08 | gene silencing | 0.0266 |
| GO:0007281 | 13 | 0.08 | germ cell development | 0.0316 |
| GO:0008406 | 10 | 0.10 | gonad development | 0.0193 |
| GO:0006398 | 9 | 0.11 | histone mRNA 3'-end processing | 0.0145 |
| GO:0008298 | 12 | 0.08 | intracellular mRNA localization | 0.0266 |
| GO:0045475 | 14 | 0.07 | locomotor rhythm | 0.0365 |
| GO:0008049 | 6 | 0.17 | male courtship behavior | 0.0066 |
| GO:0007141 | 5 | 0.20 | male meiosis I | 0.0038 |
| GO:0007126 | 16 | 0.13 | meiosis | 0.0055 |
| GO:0030001 | 12 | 0.08 | metal ion transport | 0.0266 |
| GO:0000226 | 9 | 0.11 | microtubule cytoskeleton organization and biogenesis | 0.0145 |
| GO:0007017 | 26 | 0.08 | microtubule-based process | 0.0226 |
| GO:0002009 | 13 | 0.08 | morphogenesis of an epithelium | 0.0316 |
| GO:0016333 | 5 | 0.20 | morphogenesis of follicular epithelium | 0.0038 |
| GO:0000184 | 5 | 0.40 | mRNA catabolism, nonsense-mediated decay | <0.0001 |
| GO:0006379 | 11 | 0.09 | mRNA cleavage | 0.0221 |
| GO:0006397 | 19 | 0.11 | mRNA processing | 0.0070 |
| GO:0006044 | 6 | 0.17 | N-acetylglucosamine metabolism | 0.0066 |
| GO:0007300 | 7 | 0.14 | nurse cell to oocyte transport (sensu Insecta) | 0.0093 |
| GO:0042048 | 12 | 0.08 | olfactory behavior | 0.0266 |
| GO:0006730 | 12 | 0.08 | one-carbon compound metabolism | 0.0266 |
| GO:0006098 | 10 | 0.20 | pentose-phosphate shunt | 0.0008 |

| GO ID | Number of Genes | Proportion accelerated | GO Term | Permutation p-value |
| --- | --- | --- | --- | --- |
| Biological Processes Cont. | | |  |  |
| GO:0007467 | 9 | 0.11 | photoreceptor cell differentiation (sensu Endopterygota) | 0.0145 |
| GO:0048066 | 12 | 0.17 | pigmentation during development | 0.0021 |
| GO:0007280 | 13 | 0.08 | pole cell migration | 0.0316 |
| GO:0007315 | 9 | 0.22 | pole plasm assembly | 0.0008 |
| GO:0045451 | 13 | 0.08 | pole plasm oskar mRNA localization | 0.0316 |
| GO:0040018 | 10 | 0.10 | positive regulation of body size | 0.0193 |
| GO:0030307 | 8 | 0.13 | positive regulation of cell growth | 0.0112 |
| GO:0006471 | 9 | 0.11 | protein amino acid ADP-ribosylation | 0.0145 |
| GO:0016579 | 14 | 0.21 | protein deubiquitination | 0.0005 |
| GO:0006606 | 19 | 0.16 | protein import into nucleus | 0.0001 |
| GO:0000059 | 11 | 0.18 | protein import into nucleus, docking | 0.0018 |
| GO:0008361 | 9 | 0.11 | regulation of cell size | 0.0145 |
| GO:0045187 | 5 | 0.20 | regulation of circadian sleep/wake cycle, sleep | 0.0038 |
| GO:0046620 | 12 | 0.08 | regulation of organ size | 0.0266 |
| GO:0006446 | 7 | 0.14 | regulation of translational initiation | 0.0093 |
| GO:0042052 | 10 | 0.10 | rhabdomere development | 0.0193 |
| GO:0006403 | 20 | 0.10 | RNA localization | 0.0084 |
| GO:0035071 | 52 | 0.08 | salivary gland cell autophagic cell death | 0.0076 |
| GO:0016330 | 6 | 0.17 | second mitotic wave (sensu Endopterygota) | 0.0066 |
| GO:0030431 | 5 | 0.20 | sleep | 0.0038 |
| GO:0007283 | 47 | 0.06 | spermatogenesis | 0.0209 |
| GO:0006614 | 8 | 0.13 | SRP-dependent cotranslational protein targeting to membrane | 0.0112 |
| GO:0006801 | 6 | 0.17 | superoxide metabolism | 0.0066 |
| GO:0016183 | 8 | 0.13 | synaptic vesicle coating | 0.0112 |
| GO:0048489 | 8 | 0.13 | synaptic vesicle transport | 0.0112 |
| GO:0007416 | 14 | 0.07 | synaptogenesis | 0.0365 |
| GO:0035147 | 6 | 0.17 | tracheal branch fusion | 0.0066 |
| GO:0006410 | 6 | 0.17 | transcription, RNA-dependent | 0.0066 |
| GO:0006413 | 42 | 0.10 | translational initiation | 0.0030 |
| GO:0008033 | 8 | 0.13 | tRNA processing | 0.0112 |
| GO:0006511 | 47 | 0.06 | ubiquitin-dependent protein catabolism | 0.0209 |
| GO:0006901 | 5 | 0.20 | vesicle coating | 0.0038 |
| GO:0045186 | 7 | 0.14 | zonula adherens assembly | 0.0093 |
