# Supplementary material for: Population Genomics: Whole-Genome Analysis of Polymorphism and Divergence in Drosophila simulans
Source: PLoS Biol. 2007 Nov 6;5(11):e310. doi: 10.1371/journal.pbio.0050310 (PMC2062478; doi:10.1371/journal.pbio.0050310)
Supplement: Supplementary file 27 [file pbio.0050310.st021.doc]

Table S21. Gene Ontology categories enriched among proteins showing accelerated protein evolution (χ^2^ test p value ≤ 0.01) in the *D. melanogaster* lineage.

| GO ID | Number of Genes | Proportion accelerated | GO Term | Permutation p-value |
| --- | --- | --- | --- | --- |
| Cellular Components | | |  |  |
| GO:0016327 | 8 | 0.13 | apicolateral plasma membrane | 0.0133 |
| GO:0005852 | 13 | 0.08 | eukaryotic translation initiation factor 3 complex | 0.0360 |
| GO:0030176 | 5 | 0.20 | integral to endoplasmic reticulum membrane | 0.0059 |
| GO:0005792 | 55 | 0.05 | microsome | 0.0400 |
| GO:0005664 | 6 | 0.17 | nuclear origin of replication recognition complex | 0.0092 |
| GO:0005778 | 6 | 0.17 | peroxisomal membrane | 0.0072 |
| GO:0005777 | 30 | 0.07 | peroxisome | 0.0323 |
| GO:0000502 | 17 | 0.12 | proteasome complex (sensu Eukaryota) | 0.0075 |
| GO:0008540 | 7 | 0.14 | proteasome regulatory particle, base subcomplex (sensu Eukaryota) | 0.0103 |
| GO:0005747 | 33 | 0.06 | respiratory chain complex I (sensu Eukaryota) | 0.0448 |
| GO:0000151 | 73 | 0.05 | ubiquitin ligase complex | 0.0274 |
| GO:0008076 | 10 | 0.10 | voltage-gated potassium channel complex | 0.0232 |
| Molecular Functions | | |  |  |
| GO:0003993 | 7 | 0.14 | acid phosphatase activity | 0.0117 |
| GO:0004022 | 6 | 0.17 | alcohol dehydrogenase activity | 0.0063 |
| GO:0004556 | 13 | 0.08 | alpha-amylase activity | 0.0334 |
| GO:0004067 | 5 | 0.20 | asparaginase activity | 0.0055 |
| GO:0004004 | 27 | 0.07 | ATP-dependent RNA helicase activity | 0.0256 |
| GO:0005544 | 7 | 0.14 | calcium-dependent phospholipid binding | 0.0106 |
| GO:0019200 | 13 | 0.08 | carbohydrate kinase activity | 0.0388 |
| GO:0015144 | 22 | 0.09 | carbohydrate transporter activity | 0.0135 |
| GO:0004568 | 13 | 0.08 | chitinase activity | 0.0388 |
| GO:0008812 | 7 | 0.43 | choline dehydrogenase activity | 0.0000 |
| GO:0004263 | 131 | 0.05 | chymotrypsin activity | 0.0135 |
| GO:0004143 | 5 | 0.20 | diacylglycerol kinase activity | 0.0055 |
| GO:0003688 | 13 | 0.08 | DNA replication origin binding | 0.0347 |
| GO:0004165 | 5 | 0.20 | dodecenoyl-CoA delta-isomerase activity | 0.0054 |
| GO:0004519 | 7 | 0.14 | endonuclease activity | 0.0112 |
| GO:0004866 | 6 | 0.33 | endopeptidase inhibitor activity | 0.0003 |
| GO:0050660 | 16 | 0.19 | FAD binding | 0.0008 |
| GO:0005355 | 13 | 0.08 | glucose transporter activity | 0.0382 |
| GO:0004930 | 55 | 0.05 | G-protein coupled receptor activity | 0.0405 |
| GO:0016836 | 11 | 0.09 | hydro-lyase activity | 0.0237 |
| GO:0004467 | 6 | 0.17 | long-chain-fatty-acid-CoA ligase activity | 0.0079 |
| GO:0005041 | 5 | 0.20 | low-density lipoprotein receptor activity | 0.0051 |
| GO:0003796 | 10 | 0.20 | lysozyme activity | 0.0017 |
| GO:0008168 | 9 | 0.11 | methyltransferase activity | 0.0188 |
| GO:0008017 | 48 | 0.06 | microtubule binding | 0.0263 |
| GO:0004497 | 57 | 0.05 | monooxygenase activity | 0.0459 |
| GO:0003954 | 31 | 0.06 | NADH dehydrogenase activity | 0.0362 |
| GO:0008188 | 31 | 0.10 | neuropeptide receptor activity | 0.0057 |
| GO:0004715 | 11 | 0.09 | non-membrane spanning protein tyrosine kinase activity | 0.0269 |
| GO:0016779 | 12 | 0.08 | nucleotidyltransferase activity | 0.0321 |
| GO:0045735 | 6 | 0.17 | nutrient reservoir activity | 0.0081 |
| GO:0016491 | 171 | 0.05 | oxidoreductase activity | 0.0196 |
| GO:0016616 | 9 | 0.22 | oxidoreductase activity, acting on the CH-OH group of donors, NAD or NADP as acceptor | 0.0007 |
| GO:0030414 | 5 | 0.20 | protease inhibitor activity | 0.0057 |
| GO:0042803 | 11 | 0.09 | protein homodimerization activity | 0.0264 |
| GO:0016500 | 5 | 0.20 | protein-hormone receptor activity | 0.0044 |
| GO:0008536 | 6 | 0.17 | Ran GTPase binding | 0.0079 |
| GO:0004252 | 107 | 0.07 | serine-type endopeptidase activity | 0.0006 |
| GO:0005272 | 10 | 0.10 | sodium channel activity | 0.0203 |
| GO:0016566 | 11 | 0.09 | specific transcriptional repressor activity | 0.0252 |
| GO:0005351 | 30 | 0.07 | sugar porter activity | 0.0337 |
| GO:0015520 | 5 | 0.20 | tetracycline:hydrogen antiporter activity | 0.0059 |
| GO:0003712 | 29 | 0.07 | transcription cofactor activity | 0.0314 |
| GO:0019199 | 5 | 0.20 | transmembrane receptor protein kinase activity | 0.0057 |
| GO:0004714 | 11 | 0.09 | transmembrane receptor protein tyrosine kinase activity | 0.0264 |
| GO:0004295 | 139 | 0.05 | trypsin activity | 0.0183 |
| GO:0005459 | 5 | 0.20 | UDP-galactose transporter activity | 0.0059 |
| Biological Processes | | |  |  |
| GO:0006637 | 9 | 0.11 | acyl-CoA metabolism | 0.0166 |
| GO:0006961 | 17 | 0.12 | antibacterial humoral response (sensu Protostomia) | 0.0210 |
| GO:0006960 | 13 | 0.23 | antimicrobial humoral response (sensu Protostomia) | 0.0066 |
| GO:0006725 | 5 | 0.20 | aromatic compound metabolism | 0.0000 |
| GO:0008407 | 14 | 0.07 | bristle morphogenesis | 0.0048 |
| GO:0008643 | 39 | 0.08 | carbohydrate transport | 0.0410 |
| GO:0009056 | 527 | 0.03 | catabolism | 0.0146 |
| GO:0016998 | 6 | 0.17 | cell wall catabolism | 0.0410 |
| GO:0008203 | 13 | 0.08 | cholesterol metabolism | 0.0064 |
| GO:0006732 | 49 | 0.06 | coenzyme metabolism | 0.0391 |
| GO:0006825 | 5 | 0.20 | copper ion transport | 0.0288 |
| GO:0006270 | 13 | 0.08 | DNA replication initiation | 0.0058 |
| GO:0046843 | 14 | 0.07 | dorsal appendage formation | 0.0369 |
| GO:0007549 | 6 | 0.50 | dosage compensation | 0.0419 |
| GO:0006118 | 262 | 0.04 | electron transport | 0.0000 |
| GO:0048598 | 7 | 0.14 | embryonic morphogenesis | 0.0214 |
| GO:0009880 | 7 | 0.14 | embryonic pattern specification | 0.0118 |
| GO:0006635 | 21 | 0.14 | fatty acid beta-oxidation | 0.0114 |
| GO:0008347 | 9 | 0.11 | glial cell migration | 0.0012 |
| GO ID | Number of Genes | Proportion accelerated | GO Term | Permutation p-value |
| Biological Processes Cont. | | |  |  |
| GO:0005977 | 7 | 0.14 | glycogen metabolism | 0.0171 |
| GO:0007447 | 7 | 0.14 | imaginal disc pattern formation | 0.0108 |
| GO:0007306 | 11 | 0.09 | insect chorion formation | 0.0103 |
| GO:0008345 | 8 | 0.13 | larval locomotory behavior | 0.0256 |
| GO:0048542 | 9 | 0.11 | lymph gland development (sensu Arthropoda) | 0.0156 |
| GO:0007041 | 10 | 0.10 | lysosomal transport | 0.0190 |
| GO:0030001 | 12 | 0.08 | metal ion transport | 0.0233 |

| GO:0006120 | 32 | 0.06 | mitochondrial electron transport, NADH to ubiquinone | 0.0384 |
| --- | --- | --- | --- | --- |
| GO:0001738 | 5 | 0.20 | morphogenesis of a polarized epithelium | 0.0059 |
| GO:0006406 | 9 | 0.11 | mRNA export from nucleus | 0.0183 |
| GO:0009299 | 13 | 0.08 | mRNA transcription | 0.0341 |
| GO:0006044 | 6 | 0.17 | N-acetylglucosamine metabolism | 0.0064 |
| GO:0008285 | 7 | 0.14 | negative regulation of cell proliferation | 0.0129 |
| GO:0008156 | 7 | 0.14 | negative regulation of DNA replication | 0.0105 |
| GO:0042059 | 6 | 0.17 | negative regulation of epidermal growth factor receptor signaling pathway | 0.0061 |
| GO:0007263 | 10 | 0.10 | nitric oxide mediated signal transduction | 0.0219 |
| GO:0042048 | 12 | 0.08 | olfactory behavior | 0.0288 |
| GO:0007389 | 7 | 0.14 | pattern specification | 0.0117 |
| GO:0006098 | 10 | 0.10 | pentose-phosphate shunt | 0.0228 |
| GO:0007031 | 12 | 0.08 | peroxisome organization and biogenesis | 0.0335 |
| GO:0006796 | 29 | 0.07 | phosphate metabolism | 0.0314 |
| GO:0009401 | 15 | 0.07 | phosphoenolpyruvate-dependent sugar phosphotransferase system | 0.0516 |
| GO:0035289 | 9 | 0.11 | posterior head segmentation | 0.0178 |
| GO:0006471 | 9 | 0.11 | protein amino acid ADP-ribosylation | 0.0189 |
| GO:0006497 | 9 | 0.11 | protein amino acid lipidation | 0.0164 |
| GO:0030163 | 6 | 0.33 | protein catabolism | 0.0001 |
| GO:0016579 | 14 | 0.07 | protein deubiquitination | 0.0391 |
| GO:0000059 | 11 | 0.09 | protein import into nucleus, docking | 0.0266 |
| GO:0007205 | 5 | 0.20 | protein kinase C activation | 0.0049 |
| GO:0006625 | 10 | 0.10 | protein targeting to peroxisome | 0.0221 |
| GO:0016567 | 81 | 0.05 | protein ubiquitination | 0.0429 |
| GO:0006508 | 460 | 0.04 | proteolysis | 0.0220 |
| GO:0007594 | 8 | 0.13 | puparial adhesion | 0.0166 |
| GO:0007265 | 14 | 0.07 | Ras protein signal transduction | 0.0441 |
| GO:0006898 | 20 | 0.10 | receptor mediated endocytosis | 0.0117 |
| GO:0042127 | 11 | 0.09 | regulation of cell proliferation | 0.0266 |
| GO:0040029 | 34 | 0.09 | regulation of gene expression, epigenetic | 0.0074 |
| GO:0046620 | 12 | 0.08 | regulation of organ size | 0.0312 |
| GO ID | Number of Genes | Proportion accelerated | GO Term | Permutation p-value |
| Biological Processes Cont. | | |  |  |
| GO:0009613 | 9 | 0.11 | response to pest, pathogen or parasite | 0.0185 |
| GO:0035271 | 5 | 0.20 | ring gland development | 0.0055 |
| GO:0006401 | 11 | 0.09 | RNA catabolism | 0.0277 |
| GO:0006368 | 15 | 0.07 | RNA elongation from RNA polymerase II promoter | 0.0472 |
| GO:0006364 | 12 | 0.08 | rRNA processing | 0.0329 |
| GO:0007379 | 14 | 0.07 | segment specification | 0.0431 |
| GO:0045498 | 6 | 0.17 | sex comb development | 0.0089 |
| GO:0008202 | 72 | 0.07 | steroid metabolism | 0.0078 |
| GO:0048488 | 12 | 0.08 | synaptic vesicle endocytosis | 0.0320 |
| GO:0007416 | 14 | 0.07 | synaptogenesis | 0.0424 |
| GO:0015904 | 5 | 0.20 | tetracycline transport | 0.0052 |
| GO:0006410 | 6 | 0.17 | transcription, RNA-dependent | 0.0072 |
| GO:0006511 | 47 | 0.06 | ubiquitin-dependent protein catabolism | 0.0291 |
| GO:0006904 | 6 | 0.17 | vesicle docking during exocytosis | 0.0075 |
| GO:0007601 | 35 | 0.06 | visual perception | 0.0467 |
| GO:0009110 | 8 | 0.13 | vitamin biosynthesis | 0.0165 |
| GO:0045186 | 7 | 0.14 | zonula adherens assembly | 0.0111 |
| GO:0007354 | 7 | 0.14 | zygotic determination of anterior/posterior axis, embryo | 0.0119 |
