# Supplementary material for: Population Genomics: Whole-Genome Analysis of Polymorphism and Divergence in Drosophila simulans
Source: PLoS Biol. 2007 Nov 6;5(11):e310. doi: 10.1371/journal.pbio.0050310 (PMC2062478; doi:10.1371/journal.pbio.0050310)
Supplement: Supplementary file 28 [file pbio.0050310.st022.doc]

Table S22. Genes associated with the most-significant 5’UTR polarized MK tests (average coverage per site ≥ 2)

UTR	Synonymous

_________	_________

Gene	Name	Poly	Fix	Poly	Fix	FET	Cell. Compo.	Mol. Function	Biol. Process

________	_____	___	___	___	___	_____	__________________________	_____________________________________________	______________________________________

*CG9279*	8	38	50	21	1.8e-8	dynactin complex GO:0005869	structural constituent of cytoskeleton GO:0005200	intracellular protein transport GO:0006886

microtubule motor activity GO:0003777	microtubule based movement GO:0007018

Vesicle-mediated transport GO:0016192

*CG5518*	*sda*	7	11	64	11	0.00014	zinc ion binding; GO:0008270	mechanosensory behavior; GO:0007638

membrane alanyl aminopeptidase activity; GO:0004179	proteolysis; GO:0006508

response to mechanical stimulus; GO:0009612

*CG13396*	*fy*	0	8	29	12	0.00028	establishment and/or maintenance of cell polarity

GO:0007163

establishment of planar polarity; GO:0001736

establishment of wing hair orientation; GO:0001737

*CG8590*	*Klp3A*	2	31	17	22	0.00035	aster; GO:0005818	microtubule binding; GO:0008017	chromosome segregation; GO:0007059

*mei-352*	central plaque of spindle pole body;	ATP binding; GO:0005524	cytokinesis; GO:0000910

GO:0005823	structural constituent of cytoskeleton; GO:0005200	fertilization (sensu Metazoa); GO:0007338

cytoplasm; GO:0005737	microtubule motor activity; GO:0003777	male meiosis; GO:0007140

intermediate layer of spindle	plus-end-directed microtubule motor activity; GO:0008574	microtubule-based movement; GO:0007018	pole body; GO:0005821	mitosis; GO:0007067

kinesin complex; GO:0005871	mitotic spindle organization and biogenesis; GO:0007052

microtubule associated complex;	pronuclear fusion; GO:0007344

GO:0005875	protein targeting; GO:0006605

midbody; GO:0030496	regulation of mitosis; GO:0007088

nucleus; GO:0005634	spindle elongation; GO:0051231

spindle; GO:0005819	spindle pole body separation; GO:0000073

*CG18608*	*prod*	1	7	16	2	0.00040	chromosome; GO:0005694	chromatin binding; GO:0003682	lymph gland development (sensu Arthropoda);

chromosome, pericentric region;	GO:0048542

GO:0000775

heterochromatin; GO:0000792

*CG10062*	4	6	59	5	0.00040

*CG6005*	2	7	20	2	0.00042

*CG18522*	2	6	70	11	0.00047	oxidoreductase activity; GO:0016491	defense response; GO:0006952

electron transporter activity; GO:0005489	electron transport; GO:0006118

nucleobase, nucleoside, nucleotide and nucleic acid metabolism; GO:0006139

oxygen and reactive oxygen species metabolism;

GO:0006800

*CG7035*	*Cbp80*	0	14	17	16	0.00058	nucleus; GO:0005634	RNA cap binding; GO:0000339	nuclear mRNA splicing, via spliceosome;	snRNA cap binding complex;	GO:0000398

GO:0005846	protein biosynthesis; GO:0006412

spliceosome complex; GO:0005681

*CG4272*	3	13	28	12	0.00080
