# Supplementary material for: Population Genomics: Whole-Genome Analysis of Polymorphism and Divergence in Drosophila simulans
Source: PLoS Biol. 2007 Nov 6;5(11):e310. doi: 10.1371/journal.pbio.0050310 (PMC2062478; doi:10.1371/journal.pbio.0050310)
Supplement: Supplementary file 29 [file pbio.0050310.st023.doc]

Table S23. Genes associated with the most-significant 3’UTR polarized MK tests (average coverage per site > 2)

UTR	Synonymous

_________	_________

Gene	Name	Poly	Fix	Poly	Fix	FET p-value	Cell. Compo.	Mol. Function	Biol. Process

________	_____	____	___	___	___	__________	____________________________	____________________________________________	_______________________________________

*CG4107*	*Pcaf*	7	17	30	6	0.000035	SAGA complex; GO:0000124	histone acetyltransferase activity; GO:0004402	regulation of transcription from RNA polymerase

polytene chromosome; GO:0005700	II promoter; GO:0006357

*CG6181*	3	12	58	19	0.00008

*CG32099*	*eap*	1	6	25	2	0.00014	magnesium ion binding; GO:0000287	fatty acid biosynthesis; GO:0006633

phosphopantetheinyltransferase activity; GO:0008897	protein amino acid phosphopantetheinylation;

GO:0018215

*CG14005*	5	13	17	2	0.00019

*CG12800*	*Cyp6d4*	6	10	33	4	0.00023	membrane; GO:0016020	electron transporter activity; GO:0005489	electron transport; GO:0006118

microsome; GO:0005792	oxidoreductase activity; GO:0016491	steroid metabolism; GO:0008202

monooxygenase activity; GO:0004497

*CG6123*	7	23	13	3	0.00038

*CG7347*	*mus304*	4	11	38	11	0.00054	cytoplasm; GO:0005737	ATP-dependent DNA helicase activity; GO:0004003	DNA damage checkpoint; GO:0000077

DNA repair; GO:0006281

cell cycle checkpoint; GO:0000075

female meiosis; GO:0007143

female meiosis chromosome segregation; GO:0016321

imaginal disc development; GO:0007444

meiotic recombination; GO:0007131

mitotic checkpoint; GO:0007093

regulation of progression through syncytial blastoderm mitotic cell cycle; GO:0007348

response to DNA damage stimulus; GO:0006974

response to radiation; GO:0009314

*CG3766*	*scat*	7	15	29	8	0.00074	Golgi apparatus; GO:0005794	nucleic acid binding; GO:0003676	Golgi to vacuole transport; GO:0006896

mitochondrion; GO:0005739	carrier activity; GO:0005386	proteolysis; GO:0006508

nucleus; GO:0005634	zinc ion binding; GO:0008270	retrograde transport, endosome to Golgi;

GO:0042147

sperm individualization; GO:0007291

spermatid development; GO:0007286

*CG7869*	*SuUR*	10	26	34	19	0.0011	centric heterochromatin;	chromatin binding; GO:0003682	DNA endoreduplication; GO:0042023

GO:0005721	ATP binding; GO:0005524	DNA replication; GO:0006260

chromosome, pericentric region;	chromatin assembly or disassembly; GO:0006333

GO:0000775	negative regulation of DNA replication;

heterochromatin; GO:0000792	GO:0008156

intercalary heterochromatin; GO:0005725	negative regulation of DNA replication;

nuclear telomeric heterochromatin; GO:0005724	GO:0008156

polytene chromosome; GO:0005700

*CG7704*	*Taf5*	6	6	44	3	0.0013	SAGA complex; GO:0000124	general RNA polymerase II transcription factor activity;	regulation of transcription, DNA-dependent;	nucleus; GO:0005634	GO:0016251	GO:0006355

transcription initiation from RNA polymerase II

promoter; GO:0006367
