# Supplementary material for: Population Genomics: Whole-Genome Analysis of Polymorphism and Divergence in Drosophila simulans
Source: PLoS Biol. 2007 Nov 6;5(11):e310. doi: 10.1371/journal.pbio.0050310 (PMC2062478; doi:10.1371/journal.pbio.0050310)
Supplement: Supplementary file 30 [file pbio.0050310.st024.doc]

Table S24. Genes associated with the most-significant intron MK tests (average coverage per site ≥ 2)

Intron	Synonymous

_________	_________

Gene	Name	Poly	Fix	Poly	Fix	FET p-value	Cell. Compo.	Mol. Function	Biol. Process

_______	______	___	___	___	___	__________	___________________________	______________________________________________	_______________________________________

*CG8834*	5	11	81	7	4.3e-7	ligase activity; GO:0016874	metabolism; GO:0008152

4-coumarate-CoA ligase activity; GO:0016207

ligase activity; GO:0016874

*CG3327*	*E23*	196	172	39	4	2.5e-6	ATP-binding cassette (ABC)	ATP binding; GO:0005524	anion transport; GO:0006820

transporter complex; GO:0043190	transporter activity; GO:0005215	lipid metabolism; GO:0006629

anion channel activity; GO:0005253	lipid transport; GO:0006869

ATPase activity, coupled to transmembrane movement of

substances; GO:0042626

nucleotide binding; GO:0000166

*CG9007*	13	24	116	35	2.6e-6	DNA binding; GO:0003677	regulation of transcription, DNA-dependent;

GO:0006355

*CG9623*	*if*	286	360	49	18	5.7e-6	contractile fiber; GO:0043292	protein binding; GO:0005515	apposition of dorsal and ventral wing surfaces;

integrin complex; GO:0008305	receptor activity; GO:0004872	GO:0007475

cell adhesion molecule binding; GO:0050839	axon guidance; GO:0007411

protein binding; GO:0005515	cell adhesion; GO:0007155

cell migration; GO:0016477

cell-cell adhesion; GO:0016337

cell-matrix adhesion; GO:0007160

heterophilic cell adhesion; GO:0007157

maintenance of protein localization; GO:0045185

midgut development; GO:0007494

muscle attachment; GO:0016203

myofibril assembly; GO:0030239

regulation of cell shape; GO:0008360

salivary gland development; GO:0007431

tracheal epithelial cell migration (sensu Insecta);

GO:0007427

tracheal outgrowth (sensu Insecta); GO:0007426

wing morphogenesis; GO:0007476

*CG4314*	*st*	19	21	41	4	1.1e-5	ATP-binding cassette (ABC)	ATP binding; GO:0005524	eye pigment biosynthesis; GO:0006726

transporter complex; GO:0043190	eye pigment precursor transporter activity; GO:0005395	eye pigment precursor transport; GO:0006856

integral to plasma membrane;	ATPase activity, coupled to transmembrane movement of	ommochrome biosynthesis; GO:0006727

GO:0005887	substances; GO:0042626

nucleotide binding; GO:0000166

permease activity; GO:0015646

nucleotide binding; GO:0000166

*CG33957*	*cp309*	241	92	149	18	1.9e-5	centrosome; GO0005813	structural constituent of cytoskeleton;GO000520	cytoskeleton organization and

biogenesis;GO0007010

microtubule nucleation;GO0007020

*CG10363*	*TepIV*	38	68	41	16	1.5e-5	sugar porter activity; GO:0005351	antibacterial humoral response (sensu

protease inhibitor activity; GO:0030414	Protostomia); GO:0006961

endopeptidase inhibitor activity; GO:0004866	phosphoenolpyruvate-dependent sugar

phosphotransferase system; GO:0009401

*CG4832*	*cnn*	21	34	47	13	1.3e-5	centrosome; GO:0005813	microtubule binding; GO:0008017	central nervous system development; GO:0007417

cytoplasm; GO:0005737	ATPase activity, coupled; GO:0042623	centrosome cycle; GO:0007098

microtubule associated complex;	female meiosis chromosome segregation;

GO:0005875	GO:0016321

microtubule cytoskeleton organization and biogenesis; GO:0000226

microtubule-based process; GO:0007017

midgut development; GO:0007494

mitotic spindle organization and biogenesis; GO:0007052

nuclear division; GO:0000280

peripheral nervous system development; GO:0007422

*CG32592*	*hiw*	304	394	120	78	2.6e-5	cytoplasmic membrane-bound	ubiquitin-protein ligase activity; GO:0004842	BMP signaling pathway; GO:0030509

vesicle; GO:0016023	protein binding; GO:0005515	adult walking behavior; GO:0007628

plasma membrane; GO:0005886	zinc ion binding; GO:0008270	locomotion; GO:0040011

ubiquitin ligase complex;	negative regulation of BMP signaling pathway;	GO:0000151	GO:0030514

negative regulation of synaptic growth at neuromuscular junction; GO:0045886

protein ubiquitination; GO:0016567

regulation of synaptic growth at neuromuscular junction; GO:0008582

*CG8112*	8	19	25	4	2.7e-5	sterol O-acyltransferase activity; GO:0004772	cholesterol metabolism; GO:0008203
