# Supplementary material for: Population Genomics: Whole-Genome Analysis of Polymorphism and Divergence in Drosophila simulans
Source: PLoS Biol. 2007 Nov 6;5(11):e310. doi: 10.1371/journal.pbio.0050310 (PMC2062478; doi:10.1371/journal.pbio.0050310)
Supplement: Supplementary file 31 [file pbio.0050310.st025.doc]

Table S25. Number (frequency) of non-synonymous and non-coding polymorphisms (sites with coverage of n = 5 or n = 6 *D. simulans* alleles) for different frequency classes.

Coverage class 5

Nonsynonymous	Intron + intergenic	X chromosome

1 313 (0.65)	17249 (0.58)

2	79 (0.17)	5640 (0.19)

3	48 (0.10)	3777 (0.13)

4	38 (0.08)	3221 (0.11)

χ^2^ = 12.3 (df = 3), p = 0.0064

Nonsynonymous	Intron + intergenic	Autosomes

1 4338 (0.66)	191539 (0.61)

2 1194 (0.18)	59840 (0.19)

3	590 (0.09)	32659 (0.10)

4	462 (0.07)	27461 (0.09)

χ^2^ = 62.2 (df = 3), p = 0.0000

Coverage class 6

Nonsynonymous	Intron + intergenic	X chromosome

1 65 (0.61)	4789 (0.55)

2 21 (0.20)	1478 (0.17)

3	3 (0.03)	954 (0.11)

4	8 (0.08)	719 (0.08)

5	9 (0.08)	731 (0.08)

χ^2^ = 7.66 (df = 4), p = 0.11

Nonsynonymous	Intron + intergenic	Autosomes

1 1466 (0.62)	70325 (0.58)

2	414 (0.18)	21824 (0.18)

3	214 (0.09)	11569 (0.10)

4	147 (0.06)	8202 (0.07)

5	122 (0.05)	8433 (0.07)

χ^2^ = 18.89 (df = 4), p = 0.0008
