# Supplementary material for: Population Genomics: Whole-Genome Analysis of Polymorphism and Divergence in Drosophila simulans
Source: PLoS Biol. 2007 Nov 6;5(11):e310. doi: 10.1371/journal.pbio.0050310 (PMC2062478; doi:10.1371/journal.pbio.0050310)
Supplement: Supplementary file 32 [file pbio.0050310.st026.doc]

Table S26. Counts and substitution rates per site of preferred and unpreferred variants “fixed” along the *D. simulans* and *D. melanogaster* lineages (inferred by parsimony). Substitution rates were determined by dividing the number of preferred/unpreferred fixations by the number of unpreferred/preferred ancestral bases.

|  | *D. melanogaster* | | |  | *D. simulans* | | |
| --- | --- | --- | --- | --- | --- | --- | --- |
|  | U→P |  | P→U |  | U→P |  | P→U |
| 2L | 7175 |  | 19974 |  | 6199 |  | 7529 |
| 2R | 7751 |  | 22280 |  | 6539 |  | 8475 |
| 3L | 7525 |  | 20696 |  | 6468 |  | 8004 |
| 3R | 9504 |  | 27230 |  | 8183 |  | 10394 |
| A | 31955 |  | 90180 |  | 27389 |  | 34402 |
| X | 5939 |  | 20556 |  | 6805 |  | 8321 |
| Total | 37894 |  | 110736 |  | 34194 |  | 42723 |

|  | *D. melanogaster* | | |  | *D. simulans* | | |
| --- | --- | --- | --- | --- | --- | --- | --- |
|  | U→P |  | P→U |  | U→P |  | P→U |
| 2L | 0.0161 |  | 0.0480 |  | 0.0139 |  | 0.0181 |
| 2R | 0.0162 |  | 0.0470 |  | 0.0136 |  | 0.0179 |
| 3L | 0.0156 |  | 0.0459 |  | 0.0134 |  | 0.0177 |
| 3R | 0.0161 |  | 0.0475 |  | 0.0139 |  | 0.0181 |
| A | 0.0160 |  | 0.0471 |  | 0.0137 |  | 0.0180 |
| X | 0.0161 |  | 0.0532 |  | 0.0184 |  | 0.0215 |
| Genomic Mean | 0.0160 |  | 0.0481 |  | 0.0145 |  | 0.0186 |

*D. simulans* fixations were determined using a *D. simulans* consensus sequence so that inferences for both species were made without polymorphism data. The consensus was the *w501* allele when not ‘N’. If w501 was “N”, the consensus base was picked from the remaining genomes with the following priority (high to low): *sim4/6*, *MD199S*, *C167.4*, and *MD106TS*. Only codons with a single base substitution are included in this analysis.
