# Supplementary material for: Population Genomics: Whole-Genome Analysis of Polymorphism and Divergence in Drosophila simulans
Source: PLoS Biol. 2007 Nov 6;5(11):e310. doi: 10.1371/journal.pbio.0050310 (PMC2062478; doi:10.1371/journal.pbio.0050310)
Supplement: Supplementary file 33 [file pbio.0050310.st027.doc]

Table S27. X and A, polymorphic and fixed, preferred and unpreferred variants for sites with coverage four, five or six.

Coverage four

X	χ^2^

Poly	Fixed

Pref	944	1385

Unpref	2228	1843	119.4

A

Poly	Fixed

Pref	8546	6535

Unpref	24379	9068	1253.7

Coverage five

X

Poly	Fixed

Pref	582	710

Unpref	1434	894	92.2

A

Poly	Fixed

Pref	8007	5062

Unpref	22967	6947	1086.7

Coverage six

X

Poly	Fixed

Pref	137	143

Unpref	369	201	19.5

A

Poly	Fixed

Pref	2872	1574

Unpref	8376	2203	353.5

All 2 x 2 tables are p < 0.0001
