# Supplementary material for: Population Genomics: Whole-Genome Analysis of Polymorphism and Divergence in Drosophila simulans
Source: PLoS Biol. 2007 Nov 6;5(11):e310. doi: 10.1371/journal.pbio.0050310 (PMC2062478; doi:10.1371/journal.pbio.0050310)
Supplement: Supplementary file 34 [file pbio.0050310.st028.doc]

Table S28. Unpreferred polymorphisms (coverage five sites) occur at lower frequency than preferred polymorphisms

X

Unpref	Pref

1	978 (0.68)	295 (0.51)

2	224 (0.16)	124 (0.21)

3	132 (0.09)	75 (0.13)

4	100 (0.07)	88 (0.15)

χ^2^ = 62.8, df = 3, p < 0.0001

A

1	14684 (0.64)	4205 (0.53)

2	4469 (0.19)	1711 (0.21)

3	2312 (0.10)	1126 (0.14)

4	1502 (0.07)	965 (0.12)

χ^2^ = 449.7, df = 3, p < 0.0001
