# Supplementary material for: Population Genomics: Whole-Genome Analysis of Polymorphism and Divergence in Drosophila simulans
Source: PLoS Biol. 2007 Nov 6;5(11):e310. doi: 10.1371/journal.pbio.0050310 (PMC2062478; doi:10.1371/journal.pbio.0050310)
Supplement: Supplementary file 35 [file pbio.0050310.st029.doc]

Table S29. Genes with significant polarized MK tests have a higher proportion of preferred fixations than genes with non-significant MK tests.

Preferred	Unpreferred

Significant	1793	2153

Non-significant	9074	12152

χ^2^ = 9.81, p = 0.0017
