# Supplementary material for: Population Genomics: Whole-Genome Analysis of Polymorphism and Divergence in Drosophila simulans
Source: PLoS Biol. 2007 Nov 6;5(11):e310. doi: 10.1371/journal.pbio.0050310 (PMC2062478; doi:10.1371/journal.pbio.0050310)
Supplement: Supplementary file 36 [file pbio.0050310.st030.doc]

Table S30. Preferred, unpreferred, and noncoding GC/AT fixed variants across the genome (coverage classes 3-6).

Intron	Intergenic

Preferred	Unpreferred	GC	AT	GC	AT

21733	29965	59276	44524	191845	139614
