# Supplementary material for: Population Genomics: Whole-Genome Analysis of Polymorphism and Divergence in Drosophila simulans
Source: PLoS Biol. 2007 Nov 6;5(11):e310. doi: 10.1371/journal.pbio.0050310 (PMC2062478; doi:10.1371/journal.pbio.0050310)
Supplement: Supplementary file 37 [file pbio.0050310.st031.doc]

Table S31. Polymorphic GC variants occur at higher frequency than polymorphic AT variants. *X*-linked polymorphic GC variants occur at higher frequency than autosomal polymorphic GC variants (coverage-six polymorphisms from intergenic and intron DNA)

*X* chromosome	Autosomes

GC	AT	GC/AT ratio	GC	AT	GC/AT ratio

1	1778	1678	1.06	23499	26512	0.89

2	556	509	1.09	7877	7666	1.03

3	388	307	1.26	4458	3927	1.14

4	294	225	1.31	3273	2650	1.24

5	312	232	1.34	3423	2709	1.26

Poly	3048	2951	1.03	42530	43464	0.98

Fixed	2446	1925	1.27	18138	15306	1.19
