# Supplementary material for: Population Genomics: Whole-Genome Analysis of Polymorphism and Divergence in Drosophila simulans
Source: PLoS Biol. 2007 Nov 6;5(11):e310. doi: 10.1371/journal.pbio.0050310 (PMC2062478; doi:10.1371/journal.pbio.0050310)
Supplement: Supplementary file 38 [file pbio.0050310.st032.doc]

Table S32. *D. yakuba* genome input and assembly statistics.

| Insert  Size (kb) | Vector | Reads  (millions) | Assembled Reads | Paired Reads | Coverage | Coverage >Q20 | Physical coverage |
| --- | --- | --- | --- | --- | --- | --- | --- |
| 4 | plasmid | 2.24 | 1.86 | 1.67 | 11.26 | 7.76 | 19.1 |
| 40 | fosmid | 0.15 | 0.11 | 0.08 | 0.70 | 0.38 | 9.1 |
| Total | - | 2.39 | 1.97 | 1.75 | 12.00 | 8.14 | 28.2 |

|  | >1kb | N50 length (kb) | N50 number | N70 length (kb) | N70 number | Largest (kb) |
| --- | --- | --- | --- | --- | --- | --- |
| Contigs | 20,969 | 42 | 817 | 15 | 2200 | 553 |
| Supercontigs | 11,926 | 1,024 | 29 | 156 | 122 | 7021 |

Statistics presented are for the whole genome assembly before anchored using alignments to *D*. *melanogaster*. “Contigs” are contiguous sequences not interrupted by gaps, and “supercontigs” are ordered and oriented “contigs” including estimated gap sizes. The N50 statistic is defined as the largest length L such that 50% of all nucleotides are contained in contigs of size at least L. The total contig size was 167Mb with 97% of the consensus basepairs have quality scores of at least 40 (Q40) (expected error rate of less than or equal to 10^-4^) and 98% are at least Q20.
