# Supplementary material for: Population Genomics: Whole-Genome Analysis of Polymorphism and Divergence in Drosophila simulans
Source: PLoS Biol. 2007 Nov 6;5(11):e310. doi: 10.1371/journal.pbio.0050310 (PMC2062478; doi:10.1371/journal.pbio.0050310)
Supplement: Supplementary file 39 [file pbio.0050310.st033.doc]

Table S33. Read and trim statistics for *D. simulans* syntenic assemblies.

| Line | Reads | Total Trimmed Length |
| --- | --- | --- |
| W501 | 481,632 | 354,848,599 |
| W501+fosmids | 580,276 | 419,041,009 |
| C167.4 | 273,460 | 171,870,034 |
| MD106TS | 266,561 | 184,083,646 |
| MD199S | 282,268 | 193,921,963 |
| NC48S | 269,264 | 197,296,557 |
| Sim4 | 269,770 | 187,906,486 |
| Sim6 | 318,062 | 235,344,950 |
