# Supplementary material for: Population Genomics: Whole-Genome Analysis of Polymorphism and Divergence in Drosophila simulans
Source: PLoS Biol. 2007 Nov 6;5(11):e310. doi: 10.1371/journal.pbio.0050310 (PMC2062478; doi:10.1371/journal.pbio.0050310)
Supplement: Supplementary file 40 [file pbio.0050310.st034.doc]

**Table S34. Correlation (Kendall’s t) between copy numbers of TE families in “trimmed” euchromatic regions of *D. simulans* and *melanogaster*.**

The *simulans* TEs are the “clustered” TEs. The *melanogaster* TEs are those annotated in release 4.0.

________________________________________________________________

TE class	t	p-value	n	z

LTR	0.224	0.015	64	5.59

non-LTR	0.506	4x10^-5^	40	4.16

IR	0.558	6x10^-4^	24	3.44

FB	N/A	N/A	2	N/A

________________________________________________________________

Pooled	0.359	3x10^-8^	130	5.59

________________________________________________________________
