# Supplementary material for: Population Genomics: Whole-Genome Analysis of Polymorphism and Divergence in Drosophila simulans
Source: PLoS Biol. 2007 Nov 6;5(11):e310. doi: 10.1371/journal.pbio.0050310 (PMC2062478; doi:10.1371/journal.pbio.0050310)
Supplement: Supplementary file 41 [file pbio.0050310.st035.doc]

**Table S35. Tests of the homogeneity of the proportions of each family across six *D. simulans* lines, homogeneity of classes across lines and homogeneity of families within classes across lines.** __________________________________________________________________________

Number	*χ*^2^	Prob. <

All families	126	675.7	0.07

All classes	6	50.6	0.0015

Families within LTR	64	299.1	0.74

Families within non-LTR	39	184.8	0.60

Families within IR	19	122.8	0.01

Families within FB	2	9.8	0.06

___________________________________________________________________________
