# Supplementary material for: Population Genomics: Whole-Genome Analysis of Polymorphism and Divergence in Drosophila simulans
Source: PLoS Biol. 2007 Nov 6;5(11):e310. doi: 10.1371/journal.pbio.0050310 (PMC2062478; doi:10.1371/journal.pbio.0050310)
Supplement: Supplementary file 42 [file pbio.0050310.st036.doc]

**Table S36. Test of the homogeneity of relative family copy numbers across the five chromosome arms (pooled across lines) for all TEs and within the four classes.**

___________________________________________________________________________

Number	*χ*^2^	Prob. <

All families	126	671.8	0.0001

All classes	6	33.5	0.03

Families within LTR	64	274.8	0.14

Families within non-LTR	39	248.3	0.0001

Families within IR	19	113.9	0.0005

Families within FB	2	6.2	0.15

___________________________________________________________________________
