# Supplementary material for: Population Genomics: Whole-Genome Analysis of Polymorphism and Divergence in Drosophila simulans
Source: PLoS Biol. 2007 Nov 6;5(11):e310. doi: 10.1371/journal.pbio.0050310 (PMC2062478; doi:10.1371/journal.pbio.0050310)
Supplement: Supplementary file 43 [file pbio.0050310.st037.doc]

**Table S37. Test of the homogeneity of relative family copy numbers on the *X* chromosome vs. the autosomes (pooled across lines) for all TEs and within the four classes.**

___________________________________________________________________________

Number	*χ*^2^	Prob. <

All families	126	148.0	0.08

All classes	6	3.6	0.6

Families within LTR	64	71.7	0.2

Families within non-LTR	39	50.7	0.1

Families within IR	19	23.2	0.2

Families within FB	2	0.1	1

___________________________________________________________________________
