# Supplementary material for: Population Genomics: Whole-Genome Analysis of Polymorphism and Divergence in Drosophila simulans
Source: PLoS Biol. 2007 Nov 6;5(11):e310. doi: 10.1371/journal.pbio.0050310 (PMC2062478; doi:10.1371/journal.pbio.0050310)
Supplement: Supplementary file 44 [file pbio.0050310.st038.doc]

**Table S38. Heterogeneity of “cloned” TEs numbers in various gene annotation elements.**

Feature	Expected	Observed	*χ*^2^	df	Prob. <

Exons	108.7	17	77.4

Introns	108.8	118	0.9

Intergenic	217.2	299	30.8

Total	434	434	109.1	2	2x10^-24^
