# Supplementary material for: Population Genomics: Whole-Genome Analysis of Polymorphism and Divergence in Drosophila simulans
Source: PLoS Biol. 2007 Nov 6;5(11):e310. doi: 10.1371/journal.pbio.0050310 (PMC2062478; doi:10.1371/journal.pbio.0050310)
Supplement: Supplementary file 45 [file pbio.0050310.st039.doc]

**Table S39. Comparison of expected *D. simulans* nucleotide heterozygosity and	divergence for 30kb windows centered on the estimated position of “clustered” TEs (+) compared to windows without clustered TEs (-).** The difference between the distributions (TEs: +/-) was tested with the Mann-Whitney U test; the p-value is in the upper position in the last column (Prob. < / ratio). The ratio of the means is also shown (lower in last column).	P-value

Arm	π|div	TEs	number	Mean	Median	Variance	ratio

X	π	+	739	0.0134	0.0137	2.73e-05	0.5

X	π	-	1440	0.0135	0.0138	2.58e-05	0.9933

X	div	+	739	0.0282	0.0281	3.91e-05	0.03

X	div	-	1440	0.0276	0.0273	3.43e-05	1.0214

2L	π	+	759	0.0192	0.0192	2.58e-05	0.0038

2L	π	-	1537	0.0186	0.0186	2.42e-05	1.0290

2L	div	+	757	0.0258	0.0255	1.71e-05	0.0001

2L	div	-	1537	0.0250	0.0248	1.67e-05	1.0316

2R	π	+	560	0.0178	0.0173	2.17e-05	0.0005

2R	π	-	1464	0.0170	0.0167	2.26e-05	1.0465

2R	div	+	560	0.0248	0.0250	2.10e-05	0.0001

2R	div	-	1464	0.0236	0.0234	2.05e-05	1.0501

3L	π	+	886	0.0185	0.0185	4.09e-05	0.009

3L	π	-	1659	0.0193	0.0191	3.65e-05	0.9605

3L	div	+	886	0.0254	0.0250	2.96e-05	0.08

3L	div	-	1659	0.0249	0.0246	2.65e-05	1.0184

3R	π	+	913	0.0171	0.0167	2.23e-05	0.6

3R	π	-	2032	0.0170	0.0167	2.07e-05	1.0088

3R	div	+	913	0.0245	0.0241	2.57e-05	0.0001

3R	div	-	2029	0.0233	0.0231	2.21e-05	1.0519
